# A comprehensive analysis of the efficacy and effectiveness of COVID-19 vaccines

**DOI:** 10.3389/fimmu.2022.945930

**Published:** 2022-08-26

**Authors:** Xiaofeng He, Jiao Su, Yu’nan Ma, Wenping Zhang, Shixing Tang

**Affiliations:** ^1^ Department of Epidemiology, School of Public Health, Southern Medical University, Guangzhou, China; ^2^ Institute of Evidence-Based Medicine, Heping Hospital Affiliated to Changzhi Medical College, Changzhi, China; ^3^ Department of biochemistry, Changzhi Medical College, Changzhi, China; ^4^ Department of Cardiothoracic Surgery, Heping Hospital Affiliated to Changzhi Medical College, Changzhi, China; ^5^ Department of Infectious Diseases, Nanfang Hospital, Southern Medical University, Guangzhou, China

**Keywords:** COVID-19, vaccine, efficacy, effectiveness, breakthrough infection

## Abstract

It is urgently needed to update the comprehensive analysis about the efficacy or effectiveness of COVID-19 vaccines especially during the COVID-19 pandemic caused by SARS-CoV-2 Delta and Omicron variants. In general, the current COVID-19 vaccines showed a cumulative efficacy of 66.4%, 79.7%, and 93.6% to prevent SARS-CoV-2 infection, symptomatic COVID-19, and severe COVID-19, respectively, but could not prevent the asymptomatic infection of SARS-CoV-2. Furthermore, the current COVID-19 vaccines could effectively prevent COVID-19 caused by the Delta variant although the incidence of breakthrough infection of the SARS-CoV-2 Delta variant increased when the intervals post full vaccination extended, suggesting the waning effectiveness of COVID-19 vaccines. In addition, one-dose booster immunization showed an effectiveness of 74.5% to prevent COVID-19 caused by the Delta variant. However, current COVID-19 vaccines could not prevent the infection of Omicron sub-lineage BA.1.1.529 and had about 50% effectiveness to prevent COVID-19 caused by Omicron sub-lineage BA.1.1.529. Furthermore, the effectiveness was 87.6% and 90.1% to prevent severe COVID-19 and COVID-19-related death caused by Omicron sub-lineage BA.2, respectively, while one-dose booster immunization could enhance the effectiveness of COVID-19 vaccines to prevent the infection and COVID-19 caused by Omicron sub-lineage BA.1.1.529 and sub-lineage BA.2. Two-dose booster immunization showed an increased effectiveness of 81.8% against severe COVID-19 caused by the Omicron sub-lineage BA.1.1.529 variant compared with one-dose booster immunization. The effectiveness of the booster immunization with RNA-based vaccine BNT162b2 or mRNA-1273 was over 75% against severe COVID-19 more than 17 weeks after booster immunization whereas the heterogenous booster immunization showed better effectiveness than homologous booster immunization. In summary, the current COVID-19 vaccines could effectively protect COVID-19 caused by Delta and Omicron variants but was less effective against Omicron variant infection. One-dose booster immunization could enhance protection capability, and two-dose booster immunization could provide additional protection against severe COVID-19.

## Introduction

In December 2019, a novel severe acute respiratory syndrome coronavirus 2 (SARS-CoV-2) was identified to cause a new severe acute respiratory disease, coronavirus disease 2019 (COVID-19) (1). Development and administration of COVID-19 vaccines are one of the most important strategies to prevent and control the COVID-19 pandemic. At present, different types of COVID-19 vaccine have been developed. Most frequently used vaccines are mRNA vaccine, viral vector-based vaccine, inactivated virus vaccine, and viral protein subunit vaccine. Till now, 194 candidate vaccines have been assessed in the preclinical development stage whereas 132 vaccines are in clinical development stage (2). As of April 23, 2022, the confirmed cumulative COVID-19 cases and deaths reached 505,817,953 and 6,213,876, respectively, while 58.75% of people have been fully vaccinated worldwide and the vaccination coverage rate of over 70% was reported in more than 60 countries according to the data of the World Health Organization (WHO) (3).

SARS-CoV-2 is continuously evolving to produce various variants, which have been classified by WHO into variants of concern (VOCs, including Alpha, Beta, Gamma, Delta, and recently identified Omicron), variants of interest (VOIs, including Lambda and Mu), or variants under monitoring (VUMs, including Kappa, Iota, and Eta) (4). The ongoing pandemic of COVID-19 caused by the Omicron variant of SARS-CoV-2 is a great challenge for the prevention and control of the COVID-19 pandemic. The SARS-CoV-2 variants can compromise the protective efficacy of COVID-19 vaccines due to the mutations in its spike protein especially the newly emerged variant Omicron which has many mutations in the S protein, and has been proved to be associated with increased transmissibility and immune evasion against the acquired immunity (5–7).

Several systematic reviews and meta-analyses have been conducted to evaluate the cumulative efficacy or effectiveness of COVID-19 vaccines (8–17), but the results need to be updated when more studies about COVID-19 vaccines to prevent the infection of Delta and Omicron variants are available. More and more studies indicated the waning immunity against SARS-CoV-2 infection and the decreased efficacy of COVID-19 vaccines to prevent the infection of Omicron variants (18–22). Therefore, booster immunization of COVID-19 vaccines has been proposed and evaluated in both clinical trials and observational studies (22–27). However, the role and effect of booster vaccination, especially two-dose booster immunization, remained to be comprehensively analyzed. Furthermore, SARS-CoV-2 infection among fully vaccinated subjects, so-called breakthrough infection, has been documented (28). It is necessary to evaluate the risk and incidence of the breakthrough infection of SARS-COV-2.

In the current study, we systematically assessed and updated the results about the efficacy and effectiveness of COVID-19 vaccines to prevent the infection of Delta and Omicron variants based on phase III randomized clinical trials (RCTs) and observational studies. We also systematically evaluated the effect of booster vaccination and the risk and incidence of breakthrough infection of the Delta variant among vaccinated people.

## Methods

This study was performed according to the statement on Preferred Reporting Items for Systematic Reviews and Meta-Analyses (**S1 PRISMA Checklist**) (29).

### Inclusion and exclusion criteria

Studies were eligible on the COVID-19 vaccine efficacy or effectiveness for inclusion if they (1) were clinical trial studies, case–control, and cohort studies (2); evaluated the efficacy or effectiveness of COVID-19 vaccines (3); reported the efficacy or effectiveness of COVID-19 vaccines and booster immunization to prevent infection of SARS-CoV-2 Delta and Omicron variants (4); compared the efficacy or effectiveness of COVID-19 vaccines between vaccinated and placebo, vaccinated and unvaccinated, or booster and non-booster individuals; and (5) reported adjusted effectiveness in the real-world studies and if (6) all outcomes must be laboratory confirmed. For the studies to utilize the same data sources for their investigations, we only included the studies based on the latest data. Moreover, breakthrough infection was selected among vaccinated subjects at discrete time points after full vaccination during the pandemic of Delta or Omicron variants.

### Search strategy

Publications were identified by searching PubMed, Embase, Web of Science, the Cochrane Library, BIOSIS Previews, WHO, and medRxiv databases to apply the following search strategies: (SARS-Cov-2 or 2019-nCOv or COVID-19 or coronavirus) AND (vaccination or vaccine) and (“2020/12/1”[Date - Publication]: “2022/3/30”[Date - Publication]). In addition, the references of the identified meta-analyses and reviews were checked if there were other relevant studies.

### Data extraction

Two authors reviewed the titles and abstracts and removed the studies that did not meet the inclusion criteria. Disagreements were resolved by discussion with the corresponding author. By using predesigned Microsoft Excel 2016 (Microsoft Corp, Redmond, WA, www.microsoft.com) Tables, authors XFH, JS, and WPZ independently extracted data. The characteristics of included studies for the COVID-19 vaccine information are shown in **Supplementary Table 1**. Adjusted results of vaccine efficacy or effectiveness were extracted for each outcome according to type of COVID-19 vaccines as well as age and gender of vaccinees (**Supplementary Tables 2** **–** **7**). We extracted vaccine efficacy or effectiveness estimates for SARS-CoV-2 infection, symptomatic COVID-19, severe COVID-19, COVID-19-related death, and asymptomatic SARS-CoV-2 infection. We used the authors’ definitions for the outcomes in a person with SARS-CoV-2 infections. In general, SARS-CoV-2 infection is defined as laboratory-confirmed SARS-CoV-2 infection with or without COVID-19 symptoms whereas asymptomatic infection refers to SARS-CoV-2 infection without clinical symptoms during at least 7 days after testing although some studies defined asymptomatic infection as absence of self-reported symptoms of COVID-19 (30, 31). Symptomatic COVID-19 refers to COVID-19 cases that are diagnosed according to the criteria with modifications from the Diagnosis and Treatment Scheme for COVID-19 released by the National Health Commission of China, the WHO, and the US Centers for Disease Control and Prevention (CDC) whereas severe COVID-19 is defined as confirmed COVID-19 cases with at least one clinical sign of severe systemic illness, such as clinical signs that are indicative of severe systemic illness; respiratory failure; evidence of shock; significant acute renal, hepatic, or neurologic dysfunction; admission to an intensive care unit; or death (32, 33).

**Figure 1:**
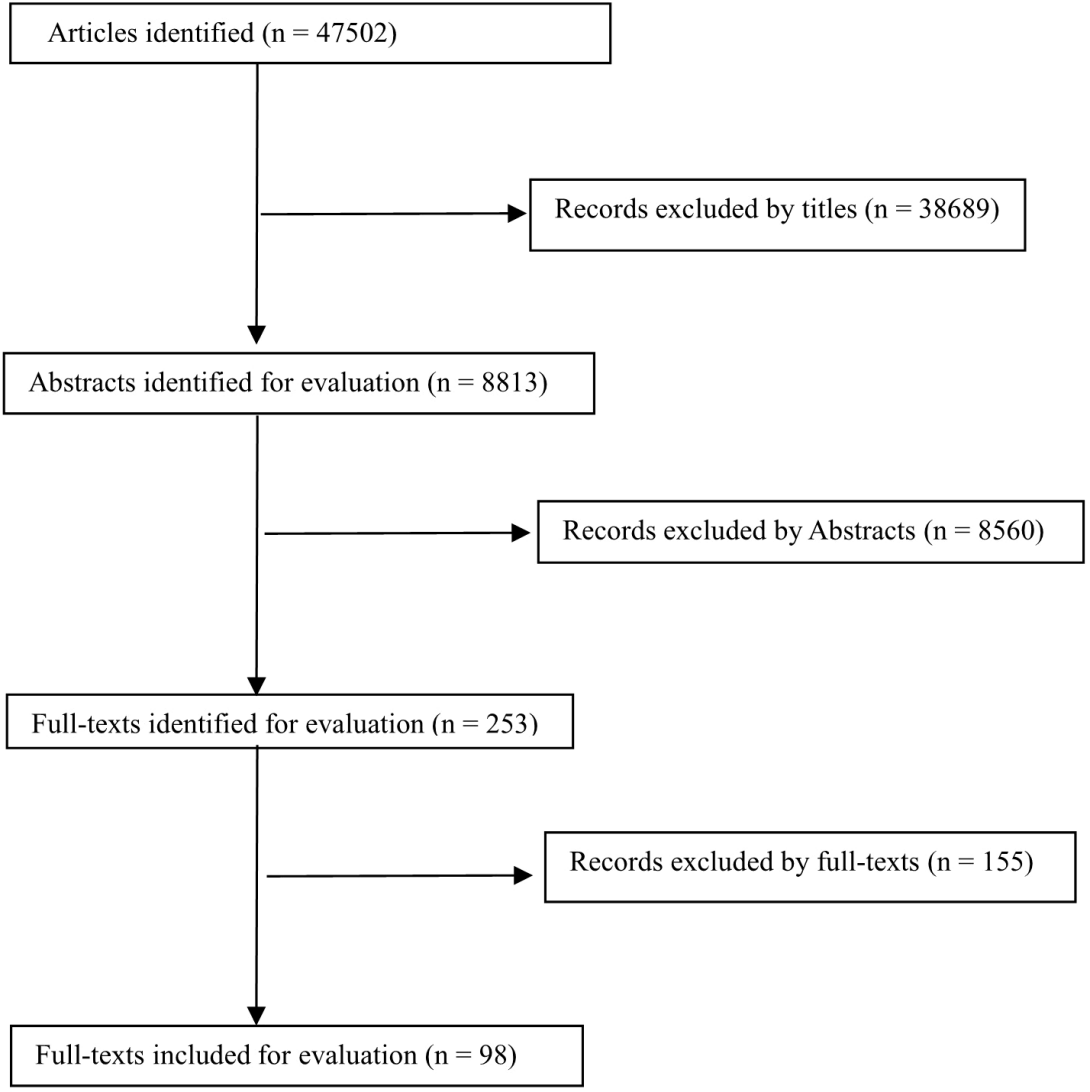
The Flow chart of literature search in the present study.

In the RCTs and observational studies, we defined full vaccination as ≥7 days after the second-dose vaccination for COVID-19 vaccines BNT162b2 and NVX-CoV2373; ≥14 days for COVID-19 vaccines BNT162b2, mRNA-1273, ChAdOx1 nCoV-19, CoronaVac, BBV152, CVnCoV, WIV04, Sputnik V, HB02, and SCB-2019; or after the single-dose vaccination for COVID-19 vaccines Ad26.COV2.S; and ≥21 days after the first-dose vaccination for COVID-19 vaccine Sputnik V (**Supplementary Tables 2**, **3**). We defined one-dose booster vaccination as ≥7, 12, or 14 days after the third shot of COVID-19 vaccines BNT162b2, mRNA-1273, ChAdOx1 nCoV-19, and CoronaVac, respectively (**Supplementary Table 4**). In addition, we included the studies that provided risk ratios, rate ratios, and odds ratios or original data for breakthrough infection of SARS-CoV-2. We specifically selected the studies that included COVID-19 cases caused by the Delta variant (**Supplementary Table 5**). A total of 47,502 publications were initially screened, of which 47,249 were irrelevant according to the title and abstract. In addition, 155 studies were excluded after reading the full text. Eventually, 98 publications met the inclusion criteria (**Figure 1**; **Supplementary Tables 2**-**7** 18–27, 32–118). Of note, a total of 27 publications including 28 RCTs (32–50, 52, 53, 55–57, 59, 108, 109) reported the symptomatic COVID-19 (**Supplementary Table 2**). Among them, seven studies (41, 44, 46, 50, 59, 108, 109) were excluded from the overall analysis because their samples overlapped with another four studies (33, 34, 43, 56). Moreover, one RNA-based vaccine candidate CVnCoV (48) was not analyzed in the present study due to their lower efficacy than the goal of 50% efficacy set by WHO. One protein subunit candidate SCB-2019 vaccine (56) was not further analyzed in the present study because it was not listed as a vaccine of emergency use by WHO.

### Risk of bias within individual studies

Two authors independently evaluated the risk of bias for eligible studies and checked the results. A ROBINS-I risk-of-bias tool was used to assess the case–control and cohort studies (119) (**Supplementary Table 9**). Moreover, a revised tool for assessing the risk of bias in randomized trials (ROBINS-2) was used (120) (**Supplementary Table 8**). The overall risk of bias was categorized as low, moderate, high, or serious in the current study.

### Statistical analysis

According to the WHO recommendation, the lowest vaccine efficacy or effectiveness and lower 95% confidence interval (CI) were set at 50% and 30%, respectively (121). The probability of type 1 error and statistical power were set at one-sided 0.025 and 0.90, respectively. The minimum sample size should be 44,488 when the incidence rate was 850 per 100,000 person-years (122). A random-effect model was applied to calculate the pooled ratio risk (RR) or odd ratio (OR) for the occurrence of COVID-19 and its 95% CI when three or more studies reported the same type of effect measure. The heterogeneity was evaluated by the Cochran *Q* test and the *I* ^2^ value (123). For RCTs, subgroup analysis was conducted according to the vaccine type as well as age and gender of vaccinees. The 95% CIs were not included if the lower bound was up to 0% and the upper bound was 100%. For the analysis of breakthrough infection, we calculated incidence rate or risk by dividing the numerator (number of cases) over the denominator (total number of persons or number of person-years). Rate ratio or RR was calculated by dividing the risk of a vaccinated group over a reference group while its 95% CI was calculated using the Byar and Taylor series methods (124, 125). Publication bias was analyzed by using Begg’s funnel plot (126) and Egger’s test (127) when the number of studies was >10. The vaccine efficacy or effectiveness was calculated as (1-RR) × 100% or (1-OR) × 100%. When a meta-analysis was not feasible, we directly described the results reported in the studies. For all the analyses, *P* < 0.05 was considered statistically significant. All statistical analyses were performed using STATA version 12.0 (STATA Corporation, College Station, TX).

## Results

### The updated efficacy of COVID-19 vaccines before the Omicron pandemic

**Figure 2:**
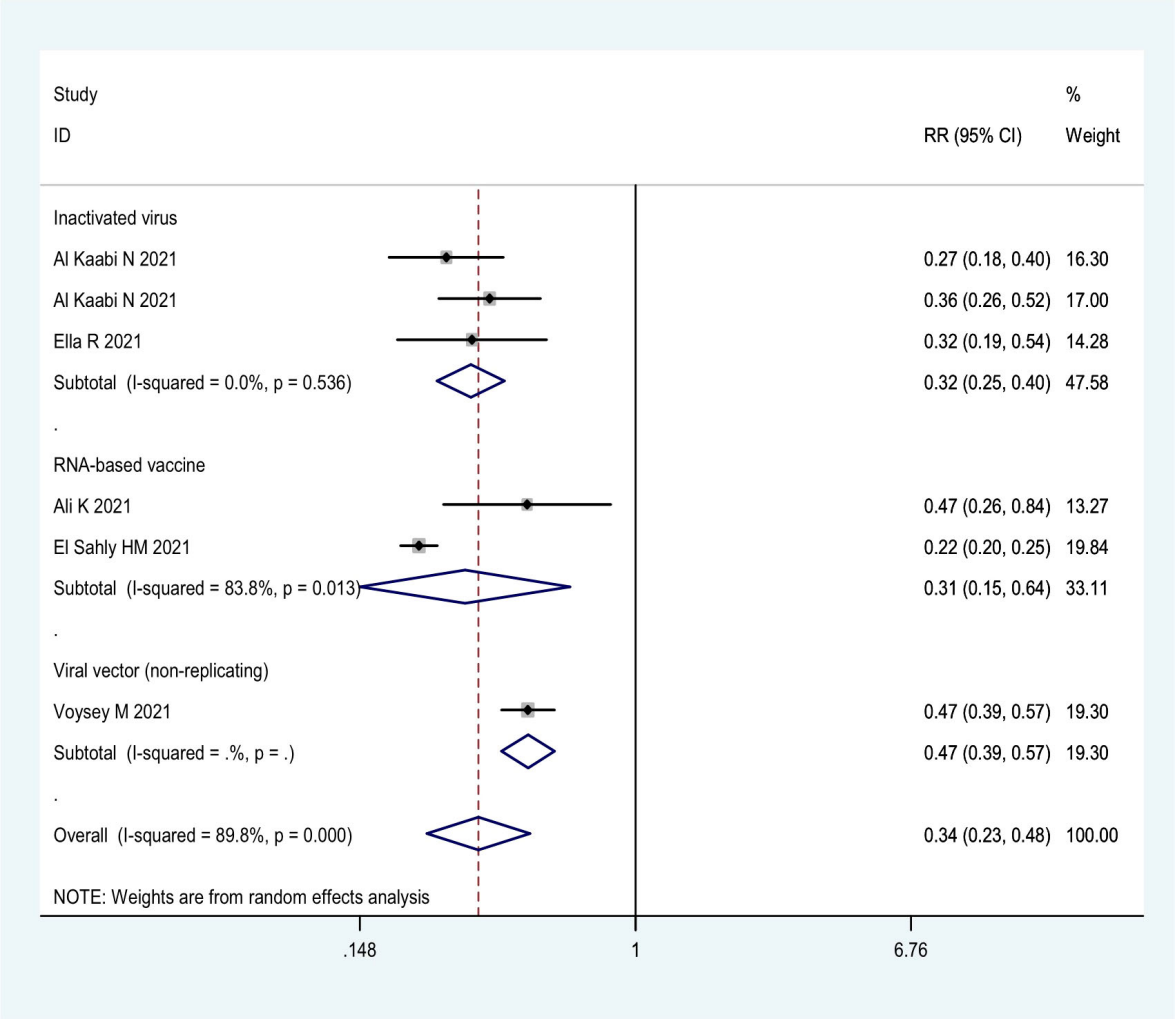
Forest plot of the efficacy of COVID-19 vaccines against SARS-CoV-2 infection in overall and different vaccine types analyses.

**Figure 3:**
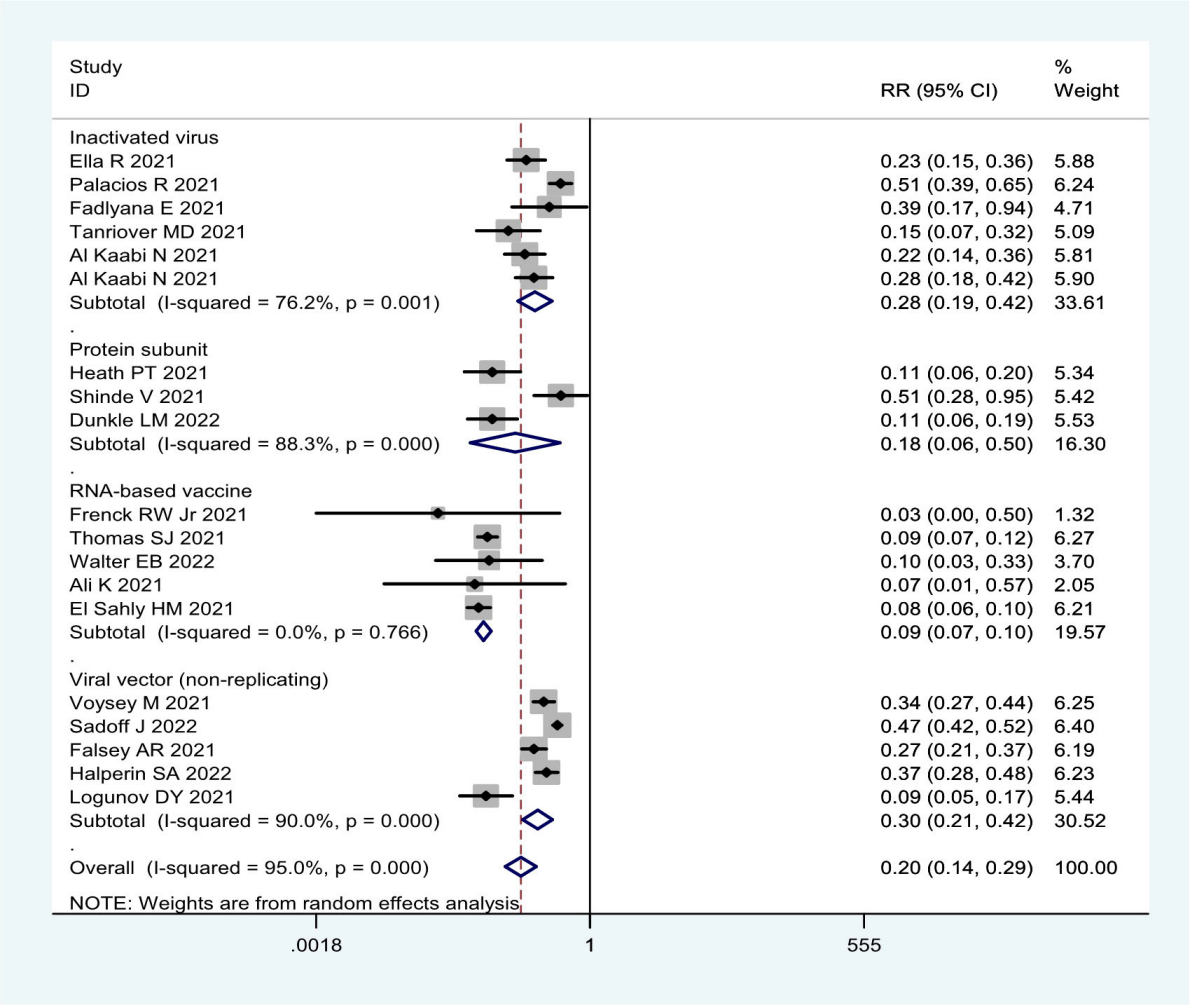
Forest plot of the efficacy of COVID-19 vaccines against symptomatic COVID-19 disease in overall and different vaccine types analyses.

**Figure 4:**
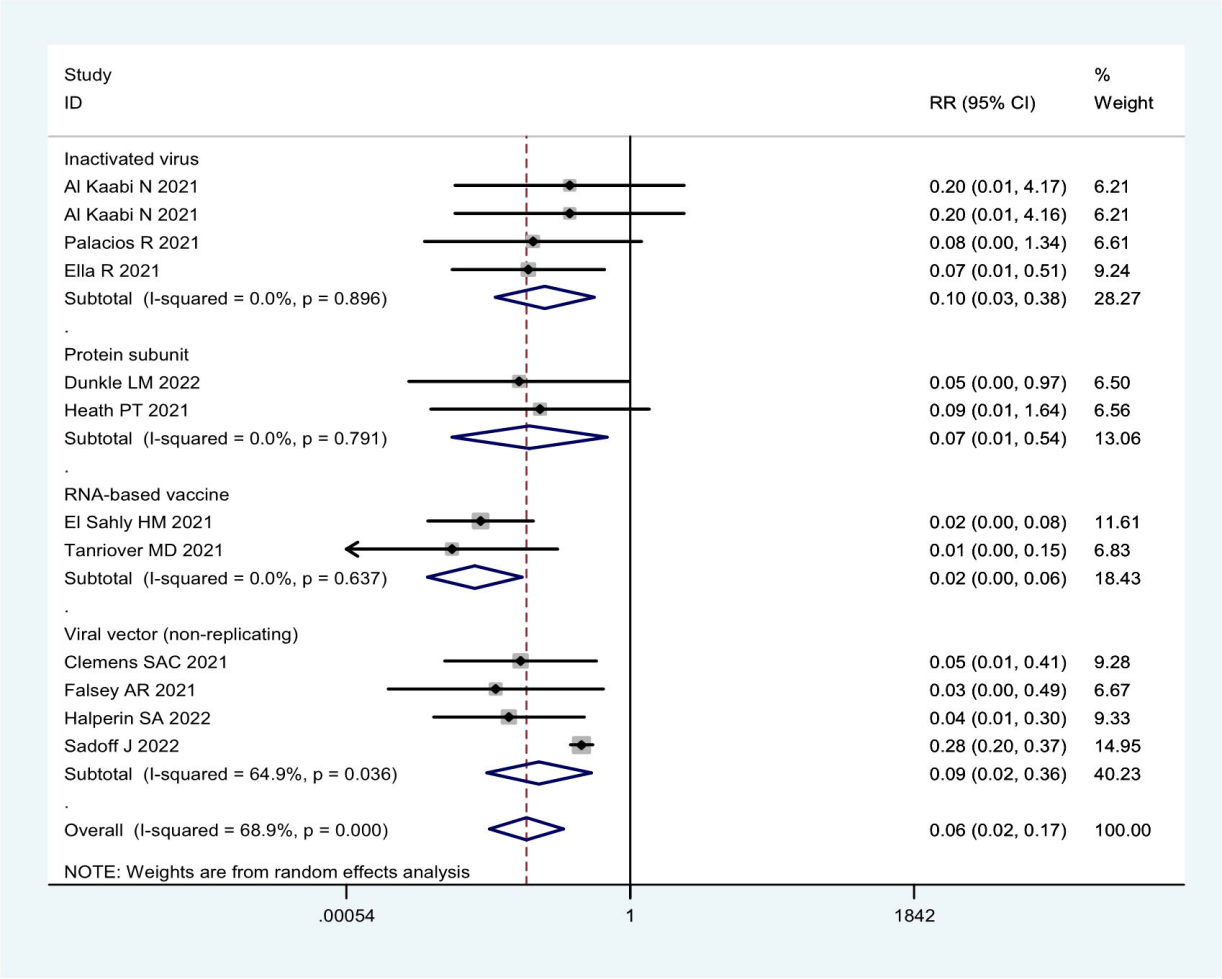
Forest plot of the efficacy of COVID-19 vaccines against severe COVID-19 disease in overall and different vaccine types analyses.

**Figure 5:**
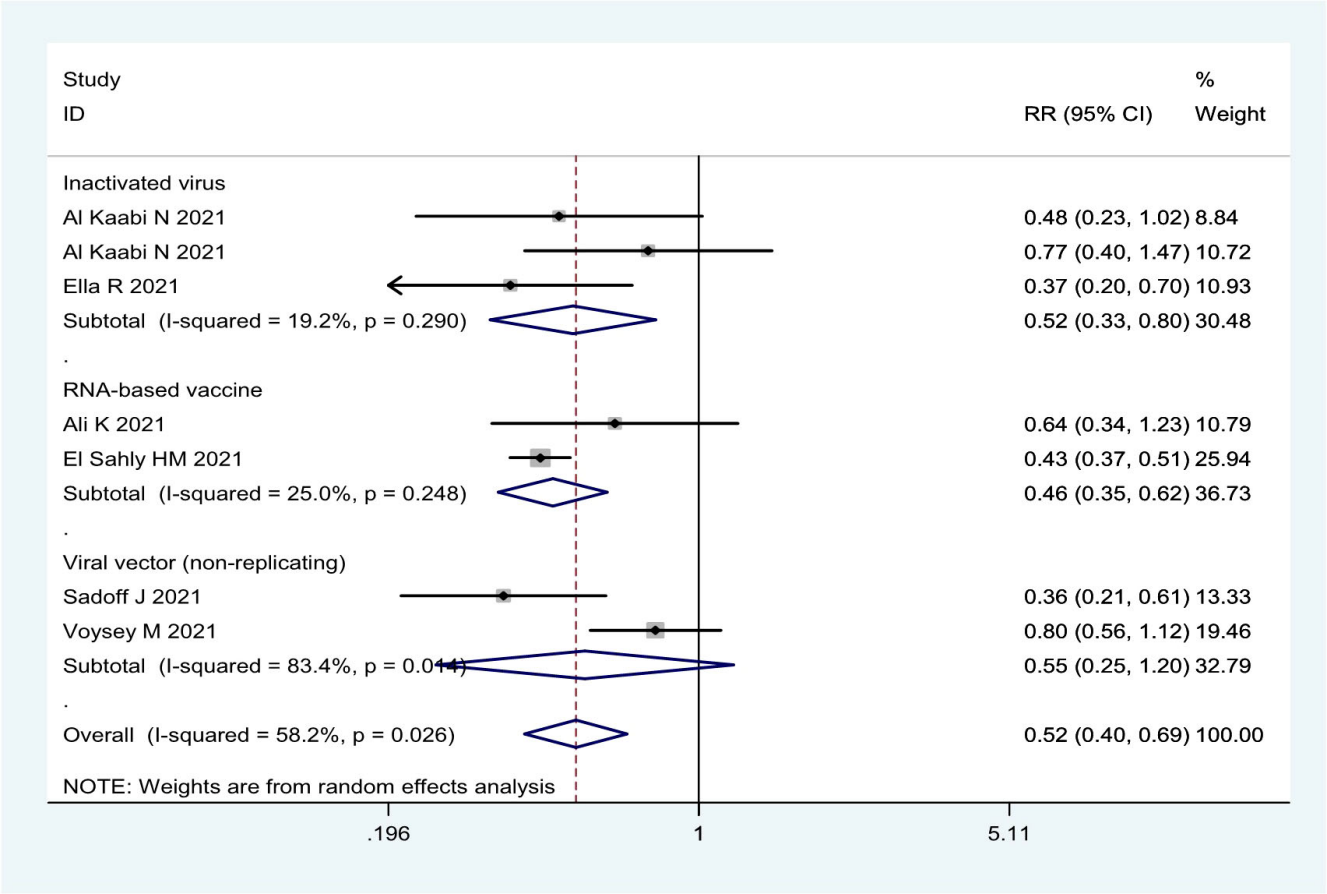
Forest plot of the efficacy of COVID-19 vaccines against asymptomatic SARS-CoV-2 infection in overall and vaccine types analyses.

We updated the efficacy analysis of COVID-19 vaccines based on the RCT studies before the pandemic of Omicron variant infection (**Supplementary Table 2**). Compared with the placebo group, the overall efficacy was 66.4% (95% CI: 51.9%, 76.5%) in preventing symptomatic infection and 47.6% (95% CI: 31.4%, 60.0%) in preventing asymptomatic infections for all COVID-19 vaccines analyzed (**Table 1** and **Figures 2**, **5**), respectively. The efficacy to prevent COVID-19 and severe COVID-19 was 79.7% (95% CI: 31.4%, 60.0%) and 93.6% (95% CI: 82.9%, 97.6%), respectively (**Table 1** and **Figures 3**, **4**), indicating that the current COVID-19 vaccines were much better in preventing severe and symptomatic COVID-19 than asymptomatic infection. There was one study to analyze the mRNA-1273 vaccine to prevent COVID-19-related death, and the efficacy was 100% (33).

**Table 1:** The efficacy of COVID-19 vaccines based on RCT studies before the Omicron pandemic.

Types of vaccine	No. of studies	RR (95% CI)	*P*_h_/*I* ^2^ (%)^&^	VE (%) (95% CI)^#^	*P*	Vaccine name
**To prevent SARS-CoV-2 infection**						
Overall	6	0.336 (0.235, 0.481)	<0.001/89.8	66.4 (51.9, 76.5)	<0.001	
RNA-based vaccine	2	0.306 (0.148, 0.635)	0.013/83.8	69.4 (36.5, 85.8)	0.001	mRNA-1273
Inactivated virus	3	0.319 (0.252, 0.403)	0.536/0.0	68.1 (59.7, 74.8)	<0.001	HB02; WIV04; BBV152
Viral vector (non-replicating)	1	0.474 (0.394, 0.570)	NA	52.6 (43.0, 61.6)	<0.001	ChAdOx1 nCoV-19
**To prevent symptomatic COVID-19**						
Overall	19	0.203 (0.141, 0.292)	<0.001/95.0	79.7 (70.8, 85.9)	<0.001	
RNA-based vaccine	5	0.086 (0.072, 0.102)	0.766/0.0	91.4 (89.8, 92.8)	<0.001	BNT162b2; mRNA-1273
Protein subunit	3	0.178 (0.063, 0.503)	< 0.001/88.3	82.2 (49.7, 93.7)	0.001	NVX-CoV2373
Inactivated virus	6	0.281 (0.189, 0.419)	0.001/76.2	71.9 (58.1, 81.1)	<0.001	BBV152; CoronaVac; HB02; WIV04
Viral vector (non-replicating)	5	0.299 (0.212, 0.423)	<0.001/90	70.1 (57.7, 78.8)	<0.001	ChAdOx1 nCoV-19; Ad26.COV2.S; Ad5-nCoV; Sputnik V
Male	9	0.203 (0.138, 0.298)	<0.001/88.6	79.7 (71.2, 86.2)	<0.001	HB02; WIV04; SCB-2019; NVX-CoV2373; mRNA-1273; BNT162b2; ChAdOx1 nCoV-19; Ad5-nCoV; Ad26.COV2.S
Female	9	0.206 (0.117, 0.364)	<0.001/92.5	79.4 (63.6, 88.3)	<0.001	
5-17 years old	4	0.083 (0.069, 0.100)	0.857/0.0	91.7 (90.0, 93.1)	<0.001	BNT162b2 and mRNA-1273
18-64 years old	2	0.085 (0.067, 0.108)	0.238/28.2	91.5 (89.2, 93.7)	<0.001	
≥65 years old	2	0.075 (0.045, 0.124)	0.388/0.0	92.5 (87.6, 95.5)	<0.001	
**To prevent severe COVID-19**						
Overall	12	0.064 (0.024, 0.171)	<0.001/68.9	93.6 (82.9, 97.6)	<0.001	
RNA-based vaccine	2	0.016 (0.005, 0.057)	0.637/0.0	98.4 (94.3, 99.5)	<0.001	BNT162b2; mRNA-1273
Protein subunit	2	0.069 (0.009, 0.540)	0.791/0.0	93.1 (46.0, 99.1)	0.011	NVX-CoV2373
Inactivated virus	4	0.103 (0.028, 0.383)	0.896/0.0	89.7 (61.7, 97.2)	0.001	HB02; WIV04; CoronaVac; BBV152
Viral vector (non-replicating)	4	0.089 (0.022, 0.356)	0.036/64.9	91.1 (64.4, 97.8)	0.001	ChAdOx1 nCoV-19; Ad5-nCoV; Ad26.COV2.S
**To prevent asymptomatic SARS-CoV-2 infection**						
Overall	7	0.524 (0.400, 0.686)	0.026/58.2	47.6 (31.4, 60.0)	<0.001	
RNA-based vaccine	2	0.464 (0.348, 0.618)	0.248/25.0	53.6 (38.2, 65.2)	<0.001	mRNA-1273
Inactivated virus	3	0.516 (0.334, 0.797)	0.290/19.2	48.4 (20.3, 66.6)	0.003	HB02; WIV04; BBV152
Viral vector (non-replicating)	2	0.549 (0.251, 1.201)	0.014/83.4	45.1 (-20.1, 74.9)	0.133	Ad26.COV2.S; ChAdOx1 nCoV-19

^#^Vaccine efficacy = 100*(1–RR) %.

^&^NA, not available.

More detailed analysis indicated that for preventing SARS-CoV-2 infection, six RCTs showed that the efficacy was 69.4% (95% CI: 36.5%, 85.8%), 68.1% (95% CI: 59.7%, 74.8%), and 52.6% (95% CI: 43.0%, 61.6%) for RNA-based vaccine mRNA-1273; three inactivated virus vaccines HB02, WIV04, and BBV152; and one trial of viral vector (non-replicating) vaccine ChAdOx1 nCoV-19, respectively (**Table 1**; **Figure 2**). For preventing COVID-19, 21 RCTs showed the efficacy of 91.4% (95% CI: 89.8%, 92.8%) for two RNA-based vaccines BNT162b2 and mRNA-1273 and 82.2% (95% CI: 49.7%, 93.7%) for protein subunit vaccine NVX-CoV2373 (**Table 1**; **Figure 3**). The efficacy of 71.9% (95% CI: 58.1%, 81.1%) and 70.1% (95% CI: 57.7%, 78.8%) was observed for four inactivated vaccines CoronaVac, WIV04, HB02, and BBV152 and four viral vector (non-replicating) vaccines Ad26.COV2.S, Ad5-nCoV, AZD1222, and Sputnik V, respectively (**Table 1**; **Figure 3**). Further subgroup analyses indicated similar efficacy for male (79.7% [95% CI: 71.2%, 86.2%]) and female (79.4% [95% CI: 63.6%, 88.3%]) subjects and for those aged 5–17 years old (91.7% [90.0%, 93.1%]),18–64 years old (91.5% [89.2%, 93.7%]), and ≥65 years old (92.5% [87.6%, 95.5%]), respectively (**Table 1**). These results indicate that age and gender may not affect the efficacy of COVID-19 vaccines to prevent COVID-19 disease.

Moreover, all COVID-19 vaccines analyzed were significantly effective in preventing severe COVID-19 in 13 RCTs (**Table 1**; **Figure 4**). Among them, two RNA-based vaccines showed the CE of 98.4% (95% CI: 94.3%, 99.5%) while the efficacy of 91.1% (95% CI: 64.4%, 97.8%) and 89.7% (95% CI: 61.7%, 97.2%) was found for four viral vector (non-replicating) vaccines and four inactivated vaccines, respectively. Furthermore, the efficacy of 93.1% (95% CI: 46.0%, 99.1%) was observed in protein subunit vaccine NVX-CoV2373 (**Table 1**; **Figure 4**).

In general, the current COVID-19 vaccines were not effective in preventing asymptomatic infection based on the seven RCTs (**Table 1**; **Figure 5**). The efficacy was only 53.6% (95% CI: 38.2%, 65.2%) for the mRNA-1273 vaccine, 48.4% (95% CI: 20.3%, 66.6%) for three inactivated vaccines (HB02, WIV04, and BBV152), and 45.1% (95% CI: -20.1%, 74.9%) for two viral vector (non-replicating) vaccines (**Table 1**; **Figure 5**).

### The effectiveness of COVID-19 vaccines to prevent SARS-CoV-2 infection of Delta variant

The case–control studies showed that the overall effectiveness was 79.5% (95% CI: 73.9%, 83.8%), 80.2% (95% CI: 74.1%, 84.9%), 95.1% (95% CI: 93.1%, 96.5%), and 92.4% (95% CI: 88.6, 94.9) to prevent Delta variant infection, COVID-19, severe COVID-19, and COVID-19-related death, respectively (**Table 2**). Although mRNA-based vaccines were better than viral-vector and inactivated vaccines in preventing Delta variant infection and COVID-19, all the COVID-19 vaccines analyzed were highly effective in preventing severe COVID-19 and COVID-19-related death caused by the Delta variant (**Table 2**).

**Table 2:** The effectiveness of COVID-19 vaccines to prevent SARS-CoV-2 Delta variant infection.

Variant	No. of studies	Adjust OR (95% CI)	*P*_h_/*I* ^2^ (%) ^&^	VE (%) (95% CI)^#^	*P*	Vaccine name
**Case–control studies**						
**To prevent SARS-CoV-2 infection**						
Overall	25	0.205 (0.161, 0.262)	<0.001/99.1	79.5 (73.9, 83.8)	<0.001	
RNA-based vaccine	14	0.179 (0.119, 0.269)	<0.001/99.4	82.1 (73.1, 88.1)	<0.001	BNT162b2; mRNA-1273
Viral vector (non-replicating)	6	0.316 (0.274, 0.366)	0.002/73.2	68.4 (63.4, 72.6)	<0.001	ChAdOx1 nCoV-19
Inactivated virus	1	0.401 (0.312, 0.513)	NA	59.9 (48.7, 68.8)	<0.001	CoronaVac
**To prevent asymptomatic SARS-CoV-2 infection**						
Overall	3	0.527 (0.457, 0.608)	0.735/0.0	47.3 (39.2, 54.3)	<0.001	BNT162b2; mRNA-1273
**To prevent symptomatic COVID-19**						
Overall	19	0.198 (0.151, 0.259)	<0.001/99.0	80.2 (74.1, 84.9)	<0.001	
RNA-based vaccine	13	0.159 (0.112, 0.226)	<0.001/98.5	84.1 (77.4, 88.8)	<0.001	BNT162b2; mRNA-1273
Viral vector (non-replicating)	5	0.342 (0.269, 0.431)	<0.001/91.5	65.8 (56.9, 73.1)	<0.001	ChAdOx1 nCoV-19
Inactivated virus	1	0.412 (0.195, 0.862)	NA	58.8 (13.8, 81.5)	0.019	CoronaVac; HB02; WIV04
12 to 17 years old	2	0.086 (0.048, 0.151)	0.104/62.1	91.4 (84.9, 95.2)	<0.001	BNT162b2
≥ 65 years old	1	0.332 (0.297, 0.366)	NA	66.8 (63.4, 70.3)	<0.001	BNT162b2
**To prevent severe COVID-19**						
Overall	25	0.049 (0.035, 0.069)	<0.001/95.8	95.1 (93.1, 96.5)	<0.001	
RNA-based vaccine	18	0.046 (0.031, 0.070)	<0.001/96.7	95.4 (93.0, 96.9)	<0.001	BNT162b2; mRNA-1273
Viral vector (non-replicating)	5	0.097 (0.071, 0.132)	0.223/29.8	90.3 (86.8, 92.9)	<0.001	ChAdOx1 nCoV-19
12 to 18 years old	1	0.077 (0.054, 0.119)	NA	92.3 (88.1, 94.6)	<0.001	BNT162b2
≥ 65 years old	2	0.108 (0.035, 0.334)	<0.001/97.0	89.2 (66.6, 96.5)	<0.001	BNT162b2, mRNA-1273
**To prevent COVID-19-related death**						
Overall	3	0.076 (0.051, 0.114)	0.198/38.2	92.4 (88.6, 94.9)	<0.001	
RNA-based vaccine	2	0.062 (0.043, 0.089)	0.737/0.0	93.8 (91.1, 95.7)	<0.001	BNT162b2; mRNA-1273
Viral vector (non-replicating)	1	0.11 (0.07, 0.20)	NA	89.0 (80.0, 93.0)	<0.001	ChAdOx1 nCoV-19
**Cohort studies**						
**To prevent SARS-CoV-2 infection**						
Overall	25	0.286 (0.252, 0.324)	<0.001/96.9	71.4 (67.6, 74.8)	<0.001	
RNA-based vaccine	16	0.254 (0.211, 0.307)	<0.001/97.5	74.6 (69.3, 78.9)	<0.001	BNT162b2; mRNA-1273
Viral vector (non-replicating)	3	0.497 (0.396, 0.624)	0.410/0.0	50.3 (37.6, 60.4)	<0.001	ChAdOx1 nCoV-19; Ad26.COV2.S
Inactivated virus	1	0.482 (0.163, 0.786)	NA	51.8 (19.4, 83.7)	0.0391	HB02; WIV04; CoronaVac
12-18 years old	4	0.128 (0.092, 0.177)	0.012/72.7	87.2 (82.3, 90.8)	<0.001	BNT162b2
60-79 years old	1	0.441 (0.393, 0.492)	NA	55.9 (50.8, 60.7)	<0.001	BNT162b2; mRNA-1273
≥ 80 years old	1	0.652 (0.514, 0.828)	NA	34.8 (17.2, 48.6)	0.001	BNT162b2; mRNA-1273
**To prevent symptomatic COVID-19**						
Overall	3	0.162 (0.056, 0.458)	0.011/77.8	83.8 (54.2, 94.4)	0.001	
RNA-based vaccine	2	0.098 (0.044, 0.216)	0.182/43.9	90.2 (78.4, 95.6)	<0.001	BNT162b2; mRNA-1273
Inactivated virus	1	0.403 (0.112, 0.684)	NA	59.7 (31.6, 88.8)	0.035	CoronaVac; HB02; WIV04
**To prevent severe COVID-19**						
Overall	6	0.103 (0.066, 0.163)	<0.001/98.8	89.7 (83.7, 93.4)	<0.001	
RNA-based vaccine	4	0.085 (0.054, 0.134)	<0.001/96.0	90.5 (86.6, 94.6)	<0.001	BNT162b2; mRNA-1273
≥ 80 years old	1	0.228 (0.176, 0.314)	NA	77.2 (68.6, 82.4)	<0.001	BNT162b2; mRNA-1273
**To prevent COVID-19-related death**						
Overall	3	0.167 (0.075, 0.370)	<0.001/97.5	83.3 (63.0, 92.5)	<0.001	BNT162b2, mRNA-1273, Ad26.COV2.S et al.

^#^Vaccine effectiveness = 100*(1–RR/OR) %.

^&^NA, not available.

Similar results were also obtained in the cohort studies about Delta variant infection (**Table 2**). In addition, mRNA-based vaccines were more effective in preventing Delta variant infection in the subjects 12–18 years old (87.2% [95% CI: 82.3%, 90.8%]) than those 60–79 years old (55.9% [95% CI: 50.8%, 60.7%]). Moreover, mRNA-based vaccines are more effective against severe COVID-19 (77.2% [95% CI: 68.6%, 82.4%]) than SARS-CoV-2 infection (34.8% [95% CI: 17.2%, 48.6%]) in the subjects ≥80 years old (**Table 2**). Similar results were also obtained in the case–control studies (**Table 2**). However, the case–control studies showed that the BNT162b2 and mRNA-1273 vaccines (47.3% [95% CI: 39.2, 54.3]) was not effective in preventing asymptomatic Delta infection (**Table 2**).

### The effectiveness of one-dose COVID-19 vaccine booster immunization against the Delta variant

Compared with unvaccinated subjects, the cohort studies showed that the overall effectiveness of one-dose booster immunization was 85.9% (95% CI: 76.2%, 91.6%) for all COVID-19 vaccines analyzed and 85.5% (95% CI: 68.7%, 93.3%) for two RNA-based vaccines (BNT162b2 and mRNA-1273) to prevent the infection caused by the Delta variant (**Table 3**). Booster immunization with two RNA-based vaccines obtained an effectiveness of 93.9% (95% CI: 91.7%, 95.4%) and 96.0% (95% CI: 93.0, 98.0) to prevent severe COVID-19 and COVID-19-related death caused by the Delta variant, respectively, while the effectiveness to prevent COVID-19 was only 78.9% (95% CI: 69.3%, 85.6%, **Table 3**). Similar results were observed in the case–control studies in which for two RNA-based vaccines, the effectiveness of booster immunization was 94.8% (95% CI: 90.7%, 97.3%) to prevent SARS-CoV-2 infection, 94.8% (95% CI: 93.6%, 95.8%) to prevent COVID-19, 97.9% (95% CI: 95.7%, 99.1%) to prevent severe COVID-19, and 96.0% (95% CI: 88.0, 99.0) to prevent COVID-19-related death caused by the Delta variant (**Table 3**).

**Table 3:** The effectiveness of booster immunization of the COVID-19 vaccine to prevent SARS-CoV-2 infection and COVID-19 caused by the Delta variant*.

Type of disease	Variant	No. of studies	RR/OR (95% CI)	*P*_h_/*I* ^2^ (%)^&^	VE (%) (95% CI)^#^	*P*	Vaccine name
**Comparison between one dose booster vaccinees and unvaccinated group**							
**Cohort studies**							
SARS-CoV-2 infection	Overall	9	0.141 (0.084, 0.238)	<0.001/98.9	85.9 (76.2, 91.6)	<0.001	
	RNA-based vaccine	6	0.145 (0.067, 0.313)	<0.001/99.3	85.5 (68.7, 93.3)	<0.001	BNT16b2; mRNA-1273
Symptomatic COVID-19	Overall	5	0.252 (0.162, 0.392)	<0.001/97.4	74.8 (60.8, 83.8)	<0.001	
	RNA-based vaccine	4	0.211 (0.144, 0.307)	<0.001/95.2	78.9 (69.3, 85.6)	<0.001	BNT16b2; mRNA-1273
Severe COVID-19	Overall	5	0.056 (0.043, 0.071)	<0.001/92.9	94.4 (92.9, 95.7)	<0.001	
	RNA-based vaccine	4	0.061 (0.046, 0.083)	<0.001/88.9	93.9 (91.7, 95.4)	<0.001	BNT16b2; mRNA-1273
COVID-19-related death	Overall	2	0.040 (0.036, 0.045)	1.000/0.0	96.0 (95.5, 96.7)	<0.001	
	RNA-based vaccine	1	0.040 (0.020, 0.070)	NA	96.0 (93.0, 98.0)	<0.001	BNT16b2; mRNA-1273
**Case–control studies**							
SARS-CoV-2 infection	Overall	5	0.062 (0.036, 0.105)	<0.001/85.8	93.8 (89.5, 96.4)	<0.001	
	RNA-based vaccine	4	0.052 (0.027, 0.093)	<0.001/87.9	94.8 (90.7, 97.3)	<0.001	BNT16b2; mRNA-1273
Symptomatic COVID-19	Overall	8	0.052 (0.042, 0.064)	<0.001/89.8	94.8 (93.6, 95.8)	<0.001	BNT16b2; mRNA-1273
Severe COVID-19	Overall	6	0.021 (0.009, 0.043)	<0.001/96.4	97.9 (95.7, 99.1)	<0.001	BNT16b2; mRNA-1273
COVID-19-related death	Overall	1	0.040 (0.010, 0.120)	NA	96.0 (88.0, 99.0)	<0.001	BNT16b2; mRNA-1273
**Comparison between one-dose booster vaccinees and non-booster vaccinees groups**							
**Cohort studies**							
SARS-CoV-2 infection	Overall	10	0.179 (0.124, 0.259)	<0.001/96.4	82.1 (74.1, 87.6)	<0.001	BNT162b2; mRNA-1273
Symptomatic COVID-19	Overall	2	0.092 (0.077, 0.105)	0.699/0.0	90.8 (89.5, 92.3)	<0.001	BNT162b2
Severe COVID-19	Overall	3	0.074 (0.038, 0.142)	0.097/57.2	92.6 (85.8, 96.2)	<0.001	BNT162b2, mRNA-1273
**Case–control studies**							
Symptomatic COVID-19	Overall	3	0.155 (0.136, 0.176)	0.012/77.3	84.5 (82.4, 86.4)	<0.001	BNT162b2; mRNA-1273

*Comparison between booster vaccinees and unvaccinated group; ^#^Vaccine effectiveness = 100*(1–RR) % or 100* (1-OR) %; ^&^NA, not available.

Compared with non-booster subjects, the cohort studies showed that the overall effectiveness of one-dose booster immunization was 82.1% (95% CI: 74.1%, 87.6%) to prevent infection of the Delta variant for two mRNA-based vaccines and 90.8% (95% CI: 89.5%, 92.3%) to prevent COVID-19 for the BNT162b2 vaccine (**Table 3**). Moreover, the overall effectiveness of one-dose booster immunization to prevent COVID-19 was 84.5% (95% CI: 82.4%, 86.4%) for BNT162b2 and mRNA-1273 vaccines based on the case–control studies (**Table 3**). Furthermore, three cohort studies showed that the effectiveness of one-dose booster immunization in preventing severe COVID-19 caused by the Delta variant was 92.6% (95% CI: 85.8%, 96.2%) for two mRNA vaccines (**Table 3**). Moreover, one cohort study (89) showed that the effectiveness of one-dose booster immunization was 84.0% (95% CI: 67.0%, 93.0%) to prevent COVID-19-related death for the BNT162b2 vaccine.

### Incidence and risk of breakthrough infection of the SARS-CoV-2 Delta variant stratified by the intervals post-vaccination and age of vaccinees

For all the COVID-19 vaccines analyzed, the incidence of breakthrough infection of the SARS-CoV-2 Delta variant increased when the intervals post-vaccination extended and was 1.1% (95% CI: 0.2%, 2.0%) within 2–10 weeks post-vaccination, 4.1% (95% CI: 0.2%, 8.1%) within 10–18 weeks post-vaccination, 6.1% (1.7%, 10.5%) within 18–26 weeks post-vaccination, 9.3% (3.4%, 15.3%) within 26–33 weeks post-vaccination, and 12.3% (4.1%, 20.5%) more than 33 weeks post-vaccination, respectively (**Table 4**). Similar results were also observed for the two mRNA-based vaccines BNT162b2 and mRNA-1273 (**Table 4**). Furthermore, the incidence of breakthrough infection of the SARS-CoV-2 Delta variant decreased from 6.0% (1.4%, 10.5%) for those 18–49 years old, 4.3% (1.2%, 7.5%) for those 50–64 years old, to 1.0% (0.1, 2.0) for those over 65 years old within 2–26 weeks post-vaccination (**Table 4**). The decreased incidence of breakthrough infection of SARS-CoV-2 Delta variant risk among old people was also found when the interval post-vaccination was more than 26 weeks (**Table 4**).

**Table 4:** Breakthrough infection and symptomatic COVID-19 of the SARS-CoV-2 Delta variant.

Type of disease	Age (year)	Weeks post final vaccination	No. of studies	*P*_h_/*I* ^2^ (%)	Incidence per 100 person years (95% CI)	RR (95% CI)	*P*	Vaccine name
RCT studies: symptomatic COVID-19	≥18	26-34	2	0.512/0.0	5.0 (4.1, 6.0)	Ref	Ref	mRNA-1273
		34-56	3	0.507/0.0	7.4 (6.6, 8.2)	1.41 (1.15, 1.73)	<0.001	
	18-64	26-34	1	NA	5.3 (4.1, 6.5)	Ref	Ref	mRNA-1273
	≥65				3.9 (2.3, 5.6)	0.76 (0.47, 1.23)	NA	
	18-64	34-56	1	NA	8.7 (7.3, 10.1)	Ref	Ref	mRNA-1273
	≥65				4.8 (3.0, 6.6)	0.57 (0.38, 0.86)	<0.001	
Cohort studies: symptomatic COVID-19	≥18	2-21	9	<0.001/99.8	0.6 (0.5, 0.8)	Ref	Ref	mRNA-1273; BNT162b2; Ad26.COV2.S
		4-26	9	<0.001/99.2	0.8 (0.6, 1.0)	1.06 (1.03, 1.08)	<0.001	
		9-34	6	<0.001/99.5	0.8 (0.6, 1.0)	NA	NA	mRNA-1273; BNT162b2
	18-49	2-21	3	<0.001/99.6	0.9 (0.6, 1.2)	Ref	Ref	mRNA-1273; BNT162b2; Ad26.COV2.S
	50-64			<0.001/99.2	0.6 (0.3, 0.8)	0.57 (0.55, 0.59)	<0.001	
	≥65			<0.001/99.2	0.4 (0.3, 0.6)	0.49 (0.47, 0.51)	<0.001	
	18-49	4-26	3	<0.001/99.2	1.2 (0.8, 1.6)	Ref	Ref	
	50-64			<0.001/97.8	0.7 (0.5, 1.0)	0.66 (0.63, 0.70)	<0.001	
	≥65			<0.001/97.8	0.5 (0.3, 0.7)	0.43 (0.41, 0.45)	<0.001	
	18-49	9-34	2	<0.001/99.5	1.1 (0.7, 1.6)	Ref	Ref	mRNA-1273; BNT162b2
	50-64			<0.001/98.2	0.7 (0.5, 1.0)	0.65 (0.62, 0.69)	<0.001	
	≥65			<0.001/99.0	0.6 (0.3, 0.8)	0.55 (0.53, 0.58)	<0.001	
Cohort studies: SARS-CoV-2 infection	≥18	2-10	3	<0.001/99.8	1.1 (0.2, 2.0)	Ref	Ref	mRNA-1273; BNT162b2; Ad26.COV2.S
		10-18		<0.001/98.7	4.1 (0.2, 8.1)	2.93 (2.84, 3.02)	<0.001	
		18-26		<0.001/98.9	6.1 (1.7, 10.5)	3.03 (2.94, 3.13)	<0.001	
		26-33		<0.001/99.6	9.3 (3.4, 15.3)	4.22 (4.07, 4.38)	<0.001	
		≥ 33		<0.001/99.7	12.3 (4.1, 20.5)	5.79 (5.52, 6.07)	<0.001	
	≥18	2-10	3	0.023/73.4	1.5 (1.4, 1.6)	Ref	Ref	mRNA-1273; BNT162b2
		10-18		<0.001/99.7	5.4 (3.5, 7.3)	2.98 (2.89, 3.07)	<0.001	
		18-26		<0.001/97.8	6.1 (4.7, 7.5)	2.92 (2.82, 3.02)	<0.001	
		26-33		<0.001/99.2	8.1 (2.8, 13.3)	3.69 (3.55, 3.84)	<0.001	
		≥ 33		0.093/58.0	10.1 (7.7, 12.5)	5.76 (5.49, 6.04)	<0.001	
	18-49	2-26	3	<0.001/97.8	6.0 (1.4, 10.5)	Ref	Ref	mRNA-1273; BNT162b2; Ad26.COV2.S
	50-64			<0.001/98.2	4.3 (1.2, 7.5)	0.83 (0.81, 0.85)	<0.001	
	≥65			<0.001/99.3	1.0 (0.1, 2.0)	0.31 (0.30, 0.32)	<0.001	
	18-49	≥ 26	2	<0.001/98.8	16.1 (10.0, 22.2)	Ref	Ref	
	50-64			0.006/86.8	11.5 (9.9, 13.0)	0.78 (0.73, 0.82)	<0.001	
	≥65			<0.001/97.3	4.8 (3.6, 6.0)	0.33 (0.31, 0.35)	<0.001	

Ref, reference; NA, not available.

RCTs showed that the incidence of COVID-19 caused by the breakthrough infection of the SARS-CoV-2 Delta variant increased from 5.0% within 26–34 weeks post-vaccination to 7.4% within 34–56 weeks post-vaccination with an RR of 0.71 (95% CI: 0.58, 0.87, p < 0.001, **Table 4**). Furthermore, the incidence of COVID-19 was significantly higher in those 18–64 years old than those over 65 years old with an RR of 0.57, 95% CI: 0.38, 0.86, p < 0.001) within 34–56 weeks post-vaccination (**Table 8**). Similar results were observed in the cohort studies and confirmed the relatively higher incidence of COVID-19 in the young people and those with extended intervals post-vaccination (**Table 4**).

Furthermore, the incidence of severe COVID-19 caused by the breakthrough infection of the Delta variant was only 0.1% and increased from 0.0% (95% CI: 0.0%, 2.0%) within 2–18 weeks post-vaccination, 0.1% (95% CI: 0.0%, 0.1%) within 18–26 weeks post-vaccination, 0.2% (95% CI: 0.0%, 0.3%) within 26–33 weeks post-vaccination, to 0.4% (95% CI: 0.1%, 0.8%) more than 33 weeks post-vaccination, respectively (**Table 5**). By comparing with the incidence of severe COVID-19 within 2–18 weeks post-vaccination, the risk ratio of severe COVID-19 increased from 8.8 (7.1, 10.9) within 18–26 weeks post-vaccination, 21.4 (17.1, 26.8) within 26–33 weeks post-vaccination, to 56.3 (44.6, 71.1) more than 33 weeks post-vaccination, respectively, for all the COVID-19 vaccines analyzed (**Table 5**). Similar results were also observed for the two mRNA-based vaccines BNT162b2 and mRNA-1273 (**Table 5**). Interestingly, severe COVID-19 was more often observed when the age of vaccinees and the intervals after vaccination increased. For example, by comparing with those 16–59 years old, the risk ratio for severe COVID-19 of the SARS-CoV-2 Delta variant was 24.38 (17.53, 33.91) for those ≥60 years old within 6–28 weeks post-vaccination (**Table 5**). These results showed a unique characteristic of breakthrough infection of SARS-CoV-2, i.e., more frequent symptomatic COVID-19 in young people and severe COVID-19 in old people.

**Table 5:** Breakthrough severe COVID-19 of the SARS-CoV-2 Delta variant by cohort studies.

Type of disease	Age (year)	Weeks post final vaccination	No. of studies	*P*_h_/*I* ^2^ (%)	Incidence per 100 person years (95% CI)	RR (95% CI)	*P*	Vaccine name
**Severe COVID-19**	≥18	≥ 2	10	<0.001/98.7	0.1 (0.0, 0.1)	NA	NA	mRNA-1273; BNT162b2; Ad26.COV2.S
		2-18	4	<0.001/94.6	0.0 (0.0, 0.0)	Ref	Ref	
		18-26	2	0.003/88.7	0.1 (0.0, 0.1)	8.8 (7.1, 10.9)	<0.001	
		26-33		0.292/10.1	0.2 (0.2, 0.3)	21.4 (17.1, 26.8)	<0.001	
		≥ 33		<0.001/84.8	0.4 (-0.0, 0.8)	56.3 (44.6, 71.1)	<0.001	
	≥18	≥ 2	15	<0.001/98.2	0.1 (0.1, 0.1)	NA	NA	mRNA-1273; BNT162b2
		2-18	6	<0.001/88.8	0.0 (0.0, 0.0)	Ref	Ref	
		18-26	3	0.003/88.7	0.1 (0.0, 0.1)	9.0 (7.0, 11.7)	<0.001	
		26-33		0.292/10.1	0.2 (0.2, 0.3)	22.1 (17.0, 28.6)	<0.001	
		≥ 33		<0.001/84.8	0.4 (-0.0, 0.8)	52.5 (40.1, 68.9)	<0.001	
	18-44	≥ 2	5	<0.001/80.4	0.0 (0.0, 0.0)	Ref	Ref	mRNA-1273; BNT162b2; Ad26.COV2.S
	45-64			<0.001/95.7	0.1 (0.0, 0.1)	3.4 (2.4, 5.0)	<0.001	
	≥65			<0.001/98.8	0.2 (0.1, 0.3)	15.2 (10.9, 21.4)	<0.001	
	18-49	2-34	3	0.048/50.7	0.0 (0.0, 0.0)	Ref	Ref	mRNA-1273; BNT162b2
	50-64			<0.001/90.9	0.0 (0.0, 0.0)	2.62 (2.21, 3.11)	<0.001	
	≥65			<0.001/97.1	0.1 (0.1, 0.1)	8.34 (7.19, 9.68)	<0.001	
	16-59	6-28	6	<0.001/76.3	0.0 (0.0, 0.0)	Ref	Ref	BNT162b2
	≥60		3	<0.001/95.1	0.0 (0.0, 0.0)	24.38 (17.53, 33.91)	<0.001	

### The effectiveness of COVID-19 vaccines to prevent SARS-CoV-2 infection of the Omicron variant

The evaluation of COVID-19 vaccines to prevent Omicron variant infection was mainly conducted in the case–control studies or cohort studies. The case–control studies indicated that the current COVID-19 vaccines could not effectively prevent the infection of Omicron sub-lineage BA.1 since the effectiveness of 32.7% (95% CI: 18.4%, 44.5%) was lower than the goal of 50% set by WHO. However, the overall effectiveness was 64.8% (95% CI: 33.4%, 81.6%) to prevent symptomatic COVID-19 and 75.6% (95% CI: 50.8%, 87.9%) to prevent severe COVID-19 caused by Omicron variant B.1.1.529, respectively (**Table 6**). The cohort studies showed a similar low effectiveness (37.8% [95% CI: 29.3%, 45.6%]) of COVID-19 vaccines to prevent the infection of Omicron sub-lineage BA.1 and marginal effectiveness of 53.3% (95% CI: 40.5%, 63.3%) to prevent severe COVID-19 caused by Omicron variant B.1.1.529 (**Table 6**).

**Table 6:** The effectiveness of COVID-19 vaccines to prevent SARS-CoV-2 Omicron variant infection in case–control and cohort studies.

Type of vaccine	No. of studies	Adjust OR/RR (95% CI)	*P*_h_/*I* ^2^ (%)^&^	VE (%) (95% CI)^#^	*P*	Vaccine name
**Case–control studies**						
**To prevent SARS-CoV-2 infection of the Omicron variant (B.1.1.529)**						
Overall	6	0.673 (0.556, 0.816)	<0.001/98.2	32.7 (18.4, 44.5)	<0.001	
RNA-based vaccine	4	0.671 (0.547, 0.823)	<0.001/96.8	32.9 (17.7, 45.3)	<0.001	BNT162b2; mRNA-1273
**To prevent symptomatic COVID-19 caused by the Omicron variant (B.1.1.529)**						
Overall	5	0.352 (0.184, 0.666)	<0.001/99.1	64.8 (33.4, 81.6)	0.001	
RNA-based vaccine	4	0.288 (0.212, 0.391)	<0.001/88.5	71.2 (60.9, 79.8)	<0.001	BNT162b2; mRNA-1273
**To prevent severe COVID-19 caused by the Omicron variant (B.1.1.529)**						
Overall	6	0.244 (0.121, 0.492)	<0.001/95.5	75.6 (50.8, 87.9)	<0.001	BNT162b2; mRNA-1273
**Cohort studies**						
**To prevent SARS-CoV-2 infection of the Omicron variant (B.1.1.529)**						
Overall	11	0.622 (0.544, 0.707)	<0.001/97.5	37.8 (29.3, 45.6)	<0.001	
RNA-based vaccine	10	0.634 (0.529, 0.761)	<0.001/97.3	36.6 (23.9, 47.1)	<0.001	BNT162b2; mRNA-1273
**To prevent severe COVID-19 caused by the Omicron variant (B.1.1.529)**						
Overall	5	0.467 (0.367, 0.595)	<0.001/85.4	53.3 (40.5, 63.3)	<0.001	
RNA-based vaccine	3	0.319 (0.172, 0.593)	0.030/71.5	68.1 (41.7, 82.8)	<0.001	BNT162b2; mRNA-1273
**To prevent severe COVID-19 caused by the Omicron variant (BA.2)**						
Overall	4	0.192 (0.109, 0.338)	<0.001/87.7	80.8 (66.2, 89.1)	<0.001	
RNA-based vaccine	2	0.124 (0.084, 0.185)	0.227/31.6	87.6 (81.5, 91.6)	<0.001	BNT162b2
Inactivated virus	2	0.291 (0.154, 0.545)	0.010/0.54	70.9 (45.5, 84.6)	<0.001	CoronaVac
**To prevent COVID-19-related death by the Omicron variant (BA.2)**						
Overall	8	0.099 (0.060, 0.164)	<0.001/88.6	90.1 (83.6, 94.0)	<0.001	
RNA-based vaccine	4	0.070 (0.043, 0.113)	0.038/64.5	93.0 (88.7, 95.7)	<0.001	BNT162b2
Inactivated virus	4	0.137 (0.070, 0.270)	<0.001/90.3	86.3 (73.0, 93.0)	<0.001	CoronaVac

^#^Vaccine effectiveness = 100*(1–RR/OR) %; ^&^ NA, not available.

Moreover, several cohort studies indicated that the effectiveness of the current COVID-19 vaccines to prevent the occurrence of severe COVID-19 and COVID-19-related death caused by Omicron sub-lineage BA.2 was 80.8% (95% CI: 66.2%, 89.1%) and 90.1% (95% CI: 83.6, 94.0), respectively. Further analysis showed that the effectiveness was 87.6% (95% CI: 81.5%, 91.6%) and 70.9% (95% CI: 45.5%, 84.6%) for mRNA and inactivated vaccines, respectively, to prevent severe COVID-19 caused by the Omicron BA.2 variant, and 93.0% (95% CI: 88.7%, 95.7%) and 86.3% (95% CI: 73.0%, 93.0%) for mRNA and inactivated vaccines, respectively, to prevent COVID-19-related death (**Table 6**). In addition, one case–control study showed the effectiveness of 75% (95% CI: 52%, 87%) to prevent COVID-19-related death for two mRNA vaccines (20) while one cohort study showed that the effectiveness was only 40% (95% CI: 35%, 45%) (101).

### The effectiveness of one-dose COVID-19 vaccine booster immunization against the Omicron variant

Compared with the unvaccinated population, the overall effectiveness of one-dose booster immunization to prevent Omicron variant infection was 63.9% (95% CI: 37.6%, 78.8%) for all the COVID-19 vaccines analyzed and 68.7% (42.2%, 83.1%) for two RNA-based vaccines based on the cohort studies (**Table 7**). Similar effectiveness was also confirmed by the case–control studies in which the overall effectiveness of one-dose booster immunization to prevent symptomatic COVID-19 caused by the Omicron B.1.1.529 variant was 64.8% (95% CI: 60.7%, 68.9%) for two RNA-based vaccines (**Table 7**). Furthermore, four cohort studies were selected to analyze the overall effectiveness of one-dose booster immunization to prevent severe COVID-19 caused by the Omicron B.1.1.529 variant, which was 84.9% (95% CI: 81.5%, 87.7%) for all the COVID-19 vaccines analyzed and 85.3% (95% CI: 78.1%, 90.1%) for two RNA-based vaccine (**Table 7**). Seven case–control studies showed that the overall effectiveness of one-dose booster immunization to prevent severe COVID-19 caused by the Omicron B.1.1.529 variant was 90.7% (86.2%, 94.2%) while the CE was 91.9% (95% CI: 86.7%, 94.8%) for two mRNA-based vaccines (**Table 7**). The effectiveness was 94.0% (95% CI: 85.0, 98.0) to prevent COVID-19-related death caused by the Omicron B.1.1.529 variant in cohort studies, and a similar result was found in case–control studies.

**Table 7:** The effectiveness of booster immunization of COVID-19 vaccines to prevent SARS-CoV-2 infection and COVID-19 caused by Omicron variants*.

Type of disease	Variant	No. of studies	RR/OR (95% CI)	*P*_h_/*I* ^2^ (%) ^&^	VE (%) (95% CI) ^#^	*P*	Vaccine name
**Omicron variant (B.1.1.529)**							
**Cohort studies：** SARS-CoV-2 infection	Overall	6	0.361 (0.212, 0.624)	<0.001/99.4	63.9 (37.6, 78.8)	<0.001	
	RNA-based vaccine	4	0.313 (0.169, 0.578)	<0.001/93.2	68.7 (42.2, 83.1)	<0.001	BNT16b2; mRNA-1273
Severe COVID-19	Overall	4	0.151 (0.123, 0.185)	<0.001/88.5	84.9 (81.5, 87.7)	<0.001	
	RNA-based vaccine	2	0.147 (0.099, 0.219)	<0.001/92.6	85.3 (78.1, 90.1)	<0.001	BNT16b2; mRNA-1273
COVID-19-related death	Overall	2	0.046 (0.003, 0.782)	<0.001/94.0	95.4 (20.8, 99.7)	<0.001	
**Case–control studies：** SARS-CoV-2 infection	Overall	3	0.393 (0.291, 0.532)	<0.001/96.7	60.7 (46.8, 70.9)	<0.001	
	RNA-based vaccine	2	0.351 (0.258, 0.477)	<0.001/98.2	64.9 (52.3, 74.2)	<0.001	BNT16b2; mRNA-1273
Symptomatic COVID-19	Overall	9	0.352 (0.311, 0.393)	<0.001/92.7	64.8 (60.7, 68.9)	<0.001	BNT16b2; mRNA-1273
Severe COVID-19	Overall	7	0.093 (0.058, 0.138)	0.053/51.8	90.7 (86.2, 94.2)	<0.001	
	RNA-based vaccine	6	0.081 (0.052, 0.133)	0.036/58.1	91.9 (86.7, 94.8)	<0.001	BNT162b2, mRNA-1273
COVID-19-related death	RNA-based vaccine	1	0.060 (0.020, 0.150)	NA	94.0 (85.0, 98.0)	<0.001	BNT162b2, mRNA-1273
**Omicron variant (BA.2)**							
Cohort studies: Symptomatic COVID-19	Overall	4	0.393 (0.281, 0.554)	0.238/29.1	60.7 (44.6, 71.9)	<0.001	
	RNA-based vaccine	2	0.282 (0.178, 0.437)	1.00/0.0	71.8 (56.3, 82.2)	<0.001	BNT16b2; mRNA-1273
	Inactivated virus	2	0.512 (0.354, 0.743)	0.837/0.0	48.8 (25.7, 64.6)	<0.001	CoronaVac
Severe COVID-19	Overall	4	0.023 (0.014, 0.037)	0.138/46.0	97.7 (96.3, 98.6)	<0.001	
	RNA-based vaccine	2	0.027 (0.014, 0.052)	0.180/44.3	97.3 (94.8, 98.6)	<0.001	BNT16b2
	Inactivated virus	2	0.022 (0.007, 0.045)	0.074/68.7	97.8 (95.5, 99.3)	<0.001	CoronaVac
COVID-19-related death	Overall	6	0.017 (0.009, 0.032)	0.415/0.2	98.3 (96.8, 99.1)	<0.001	
	RNA-based vaccine	3	0.017 (0.005, 0.052)	0.136/49.8	98.3 (94.8, 99.5)	<0.001	BNT16b2
	Inactivated virus	3	0.014 (0.005, 0.039)	0.707/0.0	98.6 (96.1, 99.5)	<0.001	CoronaVac

*Comparison between booster vaccinees and unvaccinated group; ^#^Vaccine effectiveness = 100*(1–RR) % or 100*(1–OR) %; ^&^ NA, not available.

Four cohort studies evaluated the effectiveness of one-dose booster immunization against symptomatic COVID-19 caused by the Omicron variant BA.2 and showed an effectiveness of 60.7% (95% CI: 44.6%, 71.9%, **Table 7**). Among them, the effectiveness was 71.8% (95% CI: 56.3%, 82.2%) for two RNA-based vaccines and 48.8% (95% CI: 25.7%, 64.6%) for inactivated virus vaccine CoronaVac. Moreover, the overall effectiveness of booster immunization against severe COVID-19 of the Omicron BA.2 variant was 97.7% (95% CI: 96.3%, 98.6%) (**Table 7**) whereas the effectiveness was 97.3% (95% CI: 94.8%, 98.6%) for two mRNA-based vaccines and 97.8% (95% CI: 95.5%, 99.3%) for inactivated virus vaccine CoronaVac. The overall effectiveness of booster immunization against COVID-19-related death was 98.3% (95% CI: 96.8%, 99.1%), and similar results were also found in BNT162b2 (98.3% [95% CI: 94.8%, 99.5%]) and CoronaVac vaccines (98.6% [95% CI: 96.1, 99.5]) for the Omicron BA.2 variant.

When compared with the non-booster group, we evaluated and found that the overall effectiveness of one-dose booster immunization was 61.4% (95% CI: 48.6%, 71.1%) to prevent the infection of the Omicron B.1.1.529 variant and 62.8% (95% CI: 46.2%, 74.2%) for two mRNA-based vaccines (**Table 8**). Moreover, the overall effectiveness of booster immunization to prevent symptomatic COVID-19 caused by the Omicron B.1.1.529 variant was 67.4% (95% CI: 65.6%, 69.1%) for two mRNA-based vaccines. The booster immunization effectiveness to prevent symptomatic COVID-19, severe COVID-19, and COVID-19-related death caused by the Omicron BA.2 variant was 57.8% (95% CI: 26.9%, 75.6%), 87.8% (95% CI: 82.7%, 91.3%), and 89.0% (95% CI: 81.4%, 94.0%) for mRNA-based vaccine BNT162b2, respectively (**Table 8**).

**Table 8:** The relative effectiveness of booster immunization of COVID-19 vaccines to prevent SARS-CoV-2 infection and COVID-19 caused by the Omicron variant.

Type of disease	Type of vaccine	No. of studies	RR/OR (95% CI)	*P*_h_/*I* ^2^ (%)^&^	VE (%) (95% CI)^#^	*P*	Vaccine name
One dose of COVID-19 vaccine booster immunization*							
**Omicron variant (B.1.1.529)**							
**Cohort studies:** SARS-CoV-2 infection	Overall	5	0.386 (0.289, 0.514)	<0.001/86.1	61.4 (48.6, 71.1)	<0.001	
	RNA-based vaccine	4	0.372 (0.258, 0.538)	<0.001/89.5	62.8 (46.2, 74.2)	<0.001	BNT162b2; mRNA-1273
**Case–control studies:** symptomatic COVID-19	Overall	3	0.326 (0.309, 0.344)	0.205/37.0	67.4 (65.6, 69.1)	<0.001	BNT162b2; mRNA-1273
**Omicron variant (BA.2)**							
**Cohort studies:** symptomatic COVID-19	Overall	2	0.422 (0.244, 0.731)	0.111/60.6	57.8 (26.9, 75.6)	0.002	BNT16b2
Severe COVID-19	Overall	2	0.122 (0.087, 0.173)	0.650/0.0	87.8 (82.7, 91.3)	<0.001	BNT16b2
COVID-19-related death	Overall	1	0.110 (0.060, 0.186)	NA	89.0 (81.4, 94.0)	<0.001	BNT16b2
**Two doses of COVID-19 vaccine booster immunization to prevent the Omicron variant (B.1.1.529) infection ^&^**							
**Non-randomized clinical studies:** SARS-CoV-2 infection	Overall	2	0.784 (0.568, 1.081)	0.465/0.0	21.6 (-8.1, 43.2)	0.138	BNT162b2; mRNA-1273
Symptomatic COVID-19	Overall	2	0.621 (0.432, 0.893)	0.607/0.0	37.9 (10.7, 56.8)	0.010	BNT162b2; mRNA-1273
**Cohort studies:** SARS-CoV-2 infection	Overall	3	0.508 (0.482, 0.537)	0.148/47.6	49.2 (46.3, 51.8)	<0.001	
	RNA-based vaccine	2	0.521 (0.468, 0.582)	0.058/72.1	47.9 (41.8, 53.2)	<0.001	BNT16b2
Severe COVID-19	Overall	2	0.182 (0.073, 0.459)	0.244/26.3	81.8 (54.1, 92.7)	<0.001	
COVID-19-related death	Overall	1	0.070 (0.010, 0.460)	NA	93.0 (54.0, 99.0)	<0.001	

*Comparison between booster vaccinees and non-booster vaccinees groups; ^#^Vaccine effectiveness = 100*(1–RR) % or 100*(1–OR) %^&^ Comparison between two doses of COVID-19 vaccine booster immunization and one dose of COVID-19 vaccine booster immunization groups, VE, vaccine effectiveness.

### The effectiveness of two-dose COVID-19 vaccine booster immunization overone-dose booster group against the Omicron variant

We compared the effectiveness of booster immunization of one dose vs. two doses 4 months apart. Non-randomized clinical studies showed that the two-dose booster immunization provided no additional protection against Omicron variant B.1.1.529 infection (adjusted RR: 0.784, 95% CI: 0.568, 1.081, *P*: 0.138) and symptomatic COVID-19 caused by the Omicron sub-lineage B.1.1.529 with an effectiveness of 37.9% (95% CI: 10.7, 56.8) among the young healthcare workers (**Table 8**). In addition, three cohort studies were included to evaluate the effectiveness of the fourth dose of COVID-19 vaccines to prevent Omicron B.1.1.529 variant infection and showed no significant benefit since the overall effectiveness was only 49.2% (95% CI: 46.3%, 51.8%) and 47.9% (95% CI: 41.8%, 53.2%) for the mRNA-based vaccine BNT162b2 was to prevent Omicron (B.1.1.529) infection (**Table 8**). Moreover, the overall effectiveness of the fourth dose of COVID-19 vaccine was 81.8% (95% CI: 54.1%, 92.7%) and 93.0% (95% CI: 54.0%, 99.0%) to prevent severe COVID-19 and COVID-19-related death caused by Omicron sub-lineage B.1.1.529, respectively (**Table 8**).

### Duration of COVID-19 vaccines against severe COVID-19 caused by the Omicron B.1.1.529 variant

The above analysis indicted that the current COVID-19 vaccines could not prevent Omicron variant infection (**Table 6**) and could effectively prevent symptomatic COVID-19 against the Omicron variant (**Table 6**). Moreover, the effectiveness was quickly waning against Omicron variant infection and symptomatic COVID-19 (**Supplementary Tables 10**, **11**). Cohort studies showed that the effectiveness of RNA-based vaccine BNT162b2 against severe COVID-19 caused by the Omicron B.1.1.529 variant declined from 75.0% (95% CI: 66.7%, 81.2%) 2–13 weeks and 76.0% (95% CI: 56.0%, 86.0%) 13–26 weeks to 61.0% (95% CI: 48.0%, 71.0%) more than 26 weeks post-vaccination (**Table 9**). Moreover, case–control studies indicated that the effectiveness of RNA-based vaccine BNT162b2 against severe COVID-19 caused by the Omicron B.1.1.529 variant decreased from 68.6% (95% CI: 56.0%, 77.6%) 1–14 weeks, 70.4% (95% CI: 60.8%, 77.7%) 14–27 weeks, and 72.0% (95% CI: 65.7%, 77.2%) 27–40 weeks to 42.8% (95% CI: 30.2%, 53.2%) or more than 40 weeks post-vaccination. For RNA-based vaccine mRNA-1273, cohort studies showed that the effectiveness against severe COVID-19 caused by the Omicron B.1.1.529 variant decreased from 92.0% (95% CI: 43.0%, 99.0%) 2–13 weeks and 90.0% (95% CI: 28.0%, 99.0%) 13–26 weeks to 72.0% (95% CI: 43.0%, 86.0%) or over 26 weeks. The case–control studies also found that the effectiveness against severe COVID-19 caused by the Omicron B.1.1.529 variant declined from 76.9% (95% CI: 19.2%, 93.4%) at 4–30 weeks to 64.0% (95% CI: 39.1%, 78.7%) at more than 30 weeks post-vaccination (**Table 9**). Of note, the effectiveness of RNA-based vaccine BNT162b2 against severe COVID-19 increased from 65.2% (95% CI: 43.7%, 78.5%) among the subjects aged 5–11 years within 2–9 weeks post-vaccination, 76.0% (95% CI: 71.3%, 80.0%) of those aged 12–17 years within 2–11 weeks, to 91.0% (95% CI: 79.0%, 96.0%) of those aged 70 or over years within 2–13 years (**Table 9**) probably due to the high frequency of severe COVID-19 in old people.

**Table 9:** The duration of effectiveness of COVID-19 vaccines against severe COVID-19 caused by the Omicron (B.1.1.529) variant.

Variant	No. of studies	Adjust OR/RR (95% CI)	*P*_h_/*I* ^2^ (%)^&^	Time interval of after full vaccination (week)	VE (%) (95% CI)^#^	Vaccine name	Types of vaccine
Cohort studies: Overall	18	0.250 (0.188, 0.333)	0.234/24.8	2-13	75.0 (66.7, 81.2)	BNT162b2	RNA-based vaccine
	1	0.240 (0.140, 0.440)	NA	13-26	76.0 (56.0, 86.0)		
	1	0.390 (0.290, 0.520)	NA	≥26	61.0 (48.0, 71.0)		
5-11 year	7	0.348 (0.215, 0.563)	0.292/18.0	2-9	65.2 (43.7, 78.5)		
12-17 year	9	0.240 (0.200, 0.287)	0.503/0.0	2-11	76.0 (71.3, 80.0)		
≥70 year	1	0.090 (0.040, 0.210)	NA	2-13	91.0 (79.0, 96.0)		
Case–control studies: Overall	4	0.314 (0.224, 0.440)	0.576/0.0	1-14	68.6 (56.0, 77.6)		
	4	0.296 (0.223, 0.392)	0.303/17.7	14-27	70.4 (60.8, 77.7)		
	3	0.280 (0.228, 0.343)	0.706/0.0	27-40	72.0 (65.7, 77.2)		
	3	0.572 (0.468, 0.698)	0.486/0.0	≥40	42.8 (30.2, 53.2)		
Cohort studies: Overall	1	0.080 (0.001, 0.570)	NA	2-13	92.0 (43.0, 99.0)	mRNA-1273	RNA-based vaccine
	1	0.100 (0.010, 0.720)	NA	13-26	90.0 (28.0, 99.0)		
	1	0.280 (0.140, 0.570)	NA	≥ 26	72.0 (43.0, 86.0)		
Case–control studies: Overall	1	0.231 (0.066, 0.808)	NA	4-30	76.9 (19.2, 93.4)		
	1	0.360 (0.213, 0.609)	NA	≥ 30	64.0 (39.1, 78.7)		
Cohort studies: Overall	1	0.010 (0.010, 0.020)	NA	2-13	99.0 (98.0, 99.0)	ChAdOx1 nCoV-19	Viral vector (non-replicating)
	1	0.590 (0.140, 2.140)	NA	13-26	41.0 (−140, 86.0)		
	1	0.570 (0.300, 1.100)	NA	≥ 26	43.0 (−10.0, 70.0)		
Case–control studies: Overall	1	0.501 (0.363, 0.693)	NA	2-8	49.9 (30.7, 63.7)	CoronaVac	Inactivated virus
	1	0.374 (0.337, 0.415)	NA	8-26	62.6 (58.5, 66.3)		
	1	0.430 (0.398, 0.465)	NA	≥ 26	57.0 (53.5, 60.2)		
18-60 year	1	0.312 (0.264, 0.368)	NA	≥ 26	68.8 (63.2, 73.6)		
60-74 year	1	0.411 (0.371, 0.459)	NA	≥ 26	58.9 (52.9, 64.1)		
≥75 year	1	0.538 (0.478, 0.606)	NA	≥ 26	46.2 (39.4, 52.2)		

^#^Vaccine effectiveness = 100*(1–RR/OR) %.

^&^NA, not available.

For the viral vector (non-replicating) ChAdOx1 nCoV-19 vaccine, one cohort study showed that the effectiveness against severe COVID-19 caused by the Omicron B.1.1.529 variant decreased from 99.0% (95% CI: 98.0%, 99.0%) 2–13 weeks post-vaccination to 41.0% (95% CI: −140%, 86.0%) 13–26 weeks and 43.0% (95% CI: −10.0%, 70.0%) or more than 26 weeks post-vaccination (**Table 9**). For the inactivated virus CoronaVac vaccine, one case–control study showed that the effectiveness against severe COVID-19 caused by the Omicron B.1.1.529 variant was 49.9% (95% CI: 30.7%, 63.7%) within 2–8 weeks, 62.6% (95% CI: 58.5%, 66.3%) within 8–26 weeks, and 57.0% (53.5%, 60.2%) or more than 26 weeks after full vaccination, respectively (**Table 9**). The same study also showed that the CoronaVac vaccine effectiveness against severe COVID-19 caused by the Omicron B.1.1.529 variant was higher among people aged 18–60 years (68.8% [95% CI: 63.2%, 73.6%] than those aged 60–74 years (58.9% [95% CI: 52.9%, 64.1%]) and those aged 75 or more years (46.2% [95% CI: 39.4%, 52.2%]) when the time interval was 26 weeks post-vaccination (**Table 9**).

### Duration of COVID-19 vaccine booster immunization against SARS-CoV-2 infection or COVID-19 caused by the Omicron B.1.1.529 variant

Only one case–control study (21) reported the duration of the effectiveness of RNA-based vaccine mRNA-1273 homologous booster immunization against SARS-CoV-2 infection caused by the Omicron B.1.1.529 variant, which was waning from 71.6% (95% CI: 69.7%, 73.4%) 2–8 weeks to 47.4% (95% CI: 40.5%, 53.5%) over 8 weeks post-booster immunization.

Furthermore, the effectiveness of RNA-based vaccine BNT162b2 or mRNA-1273 homologous or heterogenous booster immunization was waning from 60.3% (95% CI: 57.6%, 62.8%) 1–10 weeks post-immunization, 50.2% (95% CI: 39.1%, 59.3%) 10–15 weeks, to 45.5% (95% CI: 43.8%, 47.2%) or more than 15 weeks (**Supplementary Table 12**). For RNA-based vaccine BNT162b2 homologous booster immunization, the effectiveness decreased from 56.9% (95% CI: 50.7%, 62.4%) 1–5 weeks, 49.9% (95% CI: 42.4%, 56.5%) 4–10 weeks, to 45.7% (95% CI: 44.7%, 46.7%) 10 or more weeks. For RNA-based vaccine BNT162b2 heterogenous booster mRNA-1273, the effectiveness declined from 74.0% (95% CI: 73.1%, 74.9%) 1–2 weeks and 73.9% (73.1%, 74.6%) 2–5 weeks to 64.4% (95% CI: 62.6%, 66.1) 5–10 weeks (**Table 4**). For RNA-based vaccine mRNA-1273 homologous booster immunization, the effectiveness was waning from 55.4% (95% CI: 43.6%, 64.7%) 1–6 weeks to 38.6% (95% CI: 19.4%, 53.1%) 6–8 weeks (**Supplementary Table 12**). For RNA-based vaccine mRNA-1273 heterogenous booster BNT162b2, the effectiveness was 64.3% (95% CI: 61.7%, 66.8%) within 1–2 weeks and 64.9% (95% CI: 62.3%, 67.3%) within 2–5 weeks (**Supplementary Table 12**).

For viral vector (non-replicating) ChAdOx1 nCoV-19 homologous booster immunization, the effectiveness decreased from 57.7% (95% CI: 37.6%, 71.3%) 1–2 weeks and 55.6% (95% CI: 44.4%, 64.6%) 2–5 weeks to 46.7% (95% CI: 34.3%, 56.7%) 5–10 weeks (**Supplementary Table 12**). Moreover, for the viral vector (non-replicating) ChAdOx1 nCoV-19 heterogenous booster BNT162b2 vaccine, the effectiveness was waning from 58.8% (95% CI: 57.8%, 59.7%) 1–2 weeks and 62.4% (95% CI: 61.8%, 63.0%) 2–5 weeks, 52.9% (95% CI: 52.1%, 53.7%) 5–10 weeks, to 39.6% (95% CI: 38.0%, 41.1%) 10 or more weeks (**Supplementary Table 12**). Furthermore, for viral vector (non-replicating) ChAdOx1 nCoV-19 heterogenous booster mRNA-1273 vaccine, the effectiveness declined from 68.0% (95% CI: 67.0, 68.9%) 1–2 weeks and 70.1% (95% CI: 69.5%, 70.7%) to 60.9% (95% CI: 59.7%, 62.1%) 5–10 weeks (**Supplementary Table 12**). Our results indicated that inactivated virus CoronaVac homologous booster immunization was not effective (**Supplementary Table 12**). The inactivated virus CoronaVac plus heterogenous booster with BNT162b2 could provide marginal protection against symptomatic COVID-19 caused by the Omicron B.1.1.529 variant (56.8% [95% CI: 56.3%, 57.4%] at 1–8 weeks and 34.9% [95% CI: 34.3%, 35.6%] at 8 or more weeks, **Supplementary Table 12**).

Case–control studies showed that the effectiveness of any RNA-based vaccine BNT162b2 or mRNA-1273 homologous or heterogenous booster immunization against symptomatic COVID-19 caused by the Omicron BA.2 variant was waning from 67.6% (95% CI: 58.5%, 74.6%) 2–15 weeks to 48.4% (95% CI: 45.2%, 51.4%) 15 or more weeks post-immunization (**Supplementary Table 12**).

The effectiveness of any RNA-based vaccine BNT162b2 or mRNA-1273 homologous or heterogenous booster immunization was over 75% against severe COVID-19 at 17 or more weeks post-immunization based on the cohort studies and over 80% within 15 or more weeks according to the case–control studies (**Supplementary Table 13**). The effectiveness was over 65% against severe COVID-19 caused by the Omicron B.1.1.529 variant at more than 8 weeks post-immunization for inactivated virus CoronaVac homologous booster immunization, and 86.1% (95% CI: 85.0%, 87.1%) for the combination of inactivated virus CoronaVac and heterogenous booster immunization with BNT162b2 (**Supplementary Table 13**).

## Discussion

In the present study, we updated the results about the efficacy or effectiveness of COVID-19 vaccines before the pandemic of the Omicron variant and comprehensively analyzed the effectiveness of COVID-19 vaccines in preventing infection of Delta and Omicron variants, which are the two most important and dominant strains of SARS-CoV-2. Our results further confirm the important role of COVID-19 vaccines in preventing the occurrence of COVID-19.

Our results indicated that the current COVID-19 vaccines could effectively prevent COVID-19 illness, especially severe COVID-19 and COVID-19-related death based on phase III RCTs before the pandemic of the Omicron variant. However, a major challenge for COVID-19 vaccines is its effectiveness in preventing infection and COVID-19 caused by SARS-CoV-2 variants. In the past 2 years, the pandemic of COVID-19 was dominated by Delta and Omicron variants. The mutations in the spike protein of SARS-CoV-2 have dramatically changed its biological features including its interaction with host receptor ACE2, weakened the host immune system, and compromised vaccine efficacy (30, 31). In the present study, we confirmed the effectiveness of COVID-19 vaccines to prevent Delta variant infection, in particular the symptomatic COVID-19, severe COVID-19, and COVID-19-related death caused by the Delta variant after full vaccination. However, we found the incidence of breakthrough infection of the SARS-CoV-2 Delta variant increased when the intervals after full vaccination extended due to the waning immunity for the current vaccines. These findings are consistent with neutralization data. Several studies showed that neutralizing antibody titers induced by the first two doses of vaccines declined 6 months later to near or below the seropositive cutoff (100, 128, 129). A booster vaccination of CoronaVac then dramatically increased neutralizing antibody titers to 137·9 and 143·1 GMTs 14–28 days later (128). Moreover, one phase II RCT (129) demonstrated the potential of all vaccines tested (AZD1222 (ChAdOx1 nCoV-19), mRNA-1273, NVX-CoV2373, Ad26.COV2.S, CVnCoV, Valneva, and BNT162b2) to boost immunity following an initial course of ChAd/ChAd and of six vaccines (AZD1222 (ChAdOx1 nCoV-19), BNT162b2, mRNA-1273, NVX-CoV2373, Ad26.COV2.S, and CVnCoV) following an initial course of BNT/BNT. These results indicated that a booster dose might provide longer-lasting immunity and higher levels of protection than a two-dose schedule. Therefore, an ongoing strategy is to adapt booster immunization to enhance the protection efficacy of COVID-19 vaccines. In the present study, we observed that the effectiveness of one-dose booster immunization was over 74.5% to prevent COVID-19 caused by the Delta variant, which confirmed the role of booster immunization.

However, the SARS-CoV-2 virus is continuously mutating from the original strain to the current epidemic strains of Omicron variants. Therefore, it is urgently needed to comprehensively analyze the effectiveness of COVID-19 vaccines and booster vaccination during the pandemic of the SARS-CoV-2 Omicron variant. In the present study, our results showed that two RNA vaccines BNT162b2 and mRNA-1273 could prevent 64.8% and ~70% of symptomatic and severe COVID-19 cases caused by Omicron sub-lineage BA.1.1.529, respectively. Moreover, both RNA vaccine BNT162b2 and inactivated virus CoronaVac could effectively prevent severe COVID-19 cases and COVID-19-related death caused by Omicron sub-lineage BA.2 after full vaccination. These results indicate that the current COVID-19 vaccines are still able to prevent COVID-19 caused by the SARS-CoV-2 Omicron variant. However, the current COVID-19 vaccines could not effectively prevent the infection of the Omicron variant, suggesting that SARS-CoV-2 variants especially the Omicron variant significantly decreased the effectiveness of COVID-19 vaccines. One possibility is its decreased virulence to result in the large amount of asymptomatic infection of Omicron variants (130), which affect the overall efficacy of COVID-19 vaccines. Another possibility for the decreased efficiency of COVID-19 vaccines for the Omicron variant is likely due to a combination of extended time interval post-vaccination and the immune escape caused by the mutations in the viral spike proteins (30, 31, 131). In this study, we found that booster immunization could enhance the effectiveness of COVID-19 vaccines to prevent the infection, symptomatic COVID-19, severe COVID-19, and COVID-19-related death which were about 62%, 65%, 88%, and 94% for Omicron sub-lineage BA.1.1.529, respectively. Moreover, the overall effectiveness was about 61%, 98%, and 98% in preventing symptomatic, severe COVID-19, and COVID-19-related death caused by Omicron sub-lineage BA.2, respectively. Two doses of booster immunization could provide additional protection for severe COVID-19 and COVID-19-related death with an effectiveness of 81.8% and 93% compared with dose 3 over 3 months, respectively, but not for the infection and symptomatic COVID-19 caused by the Omicron variant. These results indicated that booster immunization could effectively protect COVID-19 illness caused by the Omicron variant. Although booster immunization is less effective for the Omicron variant against infection, it could effectively protect COVID-19 and COVID-19-related death.

Another important issue is about the duration of COVID-19 vaccination since the waning immunity post-vaccination has been repeatedly reported. In our study, we systematically evaluated the duration of the effectiveness of COVID-19 vaccines to prevent SARS-CoV-2 infection and COVID-19 during the pandemic of SARS-CoV-2 Omicron variant infection. Our results confirmed that the effectiveness of COVID-19 vaccines was rapidly waning. Fortunately, homologous or heterogenous booster immunization with RNA-based vaccine BNT162b2 or mRNA-1273 could effectively prevent severe COVID-19 with over 75% efficacy. Moreover, we found that heterogenous booster immunization showed better effectiveness than homologous booster immunization. These findings are consistent with neutralization data for the Omicron variant. Several studies indicated a reduction in neutralizing antibody activity in serum specimens by a factor of 20- to 40-fold for the neutralizing antibodies acquired after two doses of BNT162b2 vaccination against SARS-CoV-2 strains from the early pandemic and by a factor of at least 10 against the Delta variant (132–134). In serum specimens obtained from the recipients of two doses of ChAdOx1 nCoV-19, a greater reduction in neutralizing activity was also observed among the serum samples of vaccines and a large proportion of them showed neutralizing activity below the limit of quantification (134). Low neutralizing antibody responses against the Omicron variant have been observed in individuals receiving two doses and three doses of CoronaVac (135–138). However, a heterogenous booster immunization could increase neutralizing activity (132–134). In addition, one booster dose of mRNA vaccine BNT162b2 has been shown to increase the neutralizing antibodies against the Omicron variant when compared to the original inactivated CoronaVac vaccine or mRNA vaccine BNT162b2 (135–138). Several possibilities may explain a decline in the effectiveness of COVID-19 vaccines when the time interval extended after vaccination. The decreased efficacy may reflect lower vaccine effectiveness against SARS-CoV-2 variants, or the true waning immunity caused by loss of vaccine-induced immunological protection, or sampling biases that may cause large heterogeneity of the studies or inconsistent results among the studies.

Our results indicate that the overall efficacy of COVID-19 vaccines is <50% in preventing asymptomatic infection of SARS-CoV-2 (**Table 1**), indicating that the current COVID-19 vaccines are inefficient in preventing infection of SARS-CoV-2, especially against asymptomatic SARS-CoV-2 infection. Unlike traditional vaccines, WHO also defines COVID-19 rather than SARS-CoV-2 infection as the primary endpoint of the efficacy of COVID-19 vaccines (121). SARS-CoV-2 infection exhibits symptomatic COVID-19 (including severe COVID-19) and asymptomatic infection (139). Sah et al. found that 35.1% (95% CI: 30.7, 39.9) of SARS-CoV-2-infected persons never developed clinical symptoms and thus were truly asymptomatic infection (140). Hence, it is necessary to evaluate the vaccine efficacy to prevent asymptomatic SARS-CoV-2 infection. People with asymptomatic infection of SARS-CoV-2 may be an important source of COVID-19 epidemic and may inevitably distort the dynamics of the COVID-19 pandemic especially for the pandemic of the Omicron variant since they may have high viral loads even a few days prior to symptom onset and cannot be timely diagnosed (141). In the present study, the current COVID-19 vaccines were not effective in preventing asymptomatic SARS-CoV-2 infection based on the RCTs. The efficacy was only 53.6% for RNA-based vaccine mRNA-1273, 48.4% for inactivated vaccines, and 45.1% for the viral vector (non-replicating) vaccines (**Table 1**). Moreover, the case–control studies showed that the overall effectiveness was only 47.3% to prevent asymptomatic COVID-19 illness caused by Delta variant for the two RNA-based vaccines BNT162b2 and mRNA-1273. Of note, the data about asymptomatic infection of SARS-CoV-2 and the efficacy evaluation of COVID-19 vaccines are prone to some biases. Some studies did not record negative PCR results of patients, which may lead to some presymptomatic cases to be mistakenly classified as asymptomatic patients. Moreover, the recorded symptoms of patients mainly based on self-reporting data in some studies, which may not be accurate and reliable. Furthermore, the proportion of asymptomatic infections was different and is associated with SARS-CoV-2 variants (130). Therefore, the efficacy or effectiveness of COVID-19 vaccines against asymptomatic COVID-19 infection should be further evaluated and explained with caution. In addition, the reasons and factors that affect the efficacy of COVID-19 vaccines to prevent SARS-CoV-2 infection, especially asymptomatic infection, remain to be elucidated.

Furthermore, breakthrough infection is urgently needed to be investigated since the studies may provide important information for the development of refined COVID-19 vaccines and for the identification of the subjects who are susceptible to breakthrough infection of SARS-CoV-2. In the present study, we found that the risk and incidence of breakthrough infections with the delta variant were increased when the time extended after vaccination. These results confirmed the feature of waning vaccine efficacy or effectiveness over time. Moreover, young people had a higher risk of breakthrough infection, which may indicate the presence of potential confounding behavioral factors in these people to cause a higher exposure to the virus. In addition, older people had a higher risk of severe COVID-19 of the SARS-CoV-2 Delta variant due to breakthrough infection probably because they had coexisting medical conditions, which are the major risk factors associated with the occurrence of severe COVID-19. Therefore, COVID-19 vaccination should give priority to the elderly people to prevent severe COVID-19 illness.

**Figure 6:**
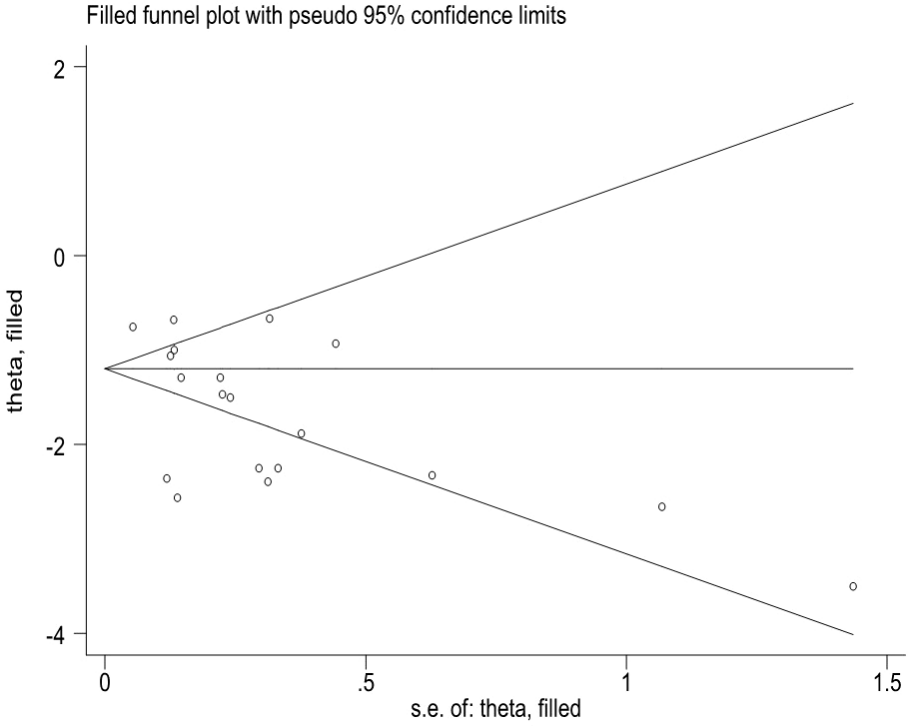
The Duval and Tweedie nonparametric "trim and fill" method's funnel plot on the efficacy of COVID-19 vaccines to prevent symptomatic COVID-19 illness based on RCT studies.

This study has several limitations and some biases. First, it is possible that additional studies on the COVID-19 vaccines’ effectiveness were not captured by our search strategy, and new studies will become available considering the rapid pace and multiple preprint publishing options for COVID-19-related papers. Second, preprint studies involved might change their results in the final publication. Third, a small number of vaccines were evaluated in observational studies that may affect the quality and reliability of the study results. Fourth, all the included studies did not evaluate the effectiveness to prevent asymptomatic Omicron infection. Fifth, we did not evaluate the effectiveness of COVID-19 vaccines among the subjects with chronic diseases and those with previous SARS-CoV-2 infections; Sixth, the major bias was incomplete adjustment for the confounders for the observational studies. Moreover, some potential biases in assessing the vaccine effectiveness over time can occur. For example, people who are vaccinated change behavior over time. Seventh, publication bias was observed on the efficacy of COVID-19 vaccines to prevent symptomatic COVID-19 illness based on RCT studies. However, when we applied a non-parametric “trim and fill” method (**Figure 6**), the analysis showed that no more study should be added. Ninth, heterogeneity between studies was much large in overall and among several subgroups. Our analysis indicated that the heterogeneity may come from the data itself since adequate statistical correction and analysis did not significantly improve the heterogeneity. The source of heterogeneity may be due to the factors such as the areas in which the studies were conducted, the risk of exposure to SARS-CoV-2, and other factors that were beyond our control, such as type of vaccines and different vaccine manufacturers. Moreover, non-reporting of negative data and heterogeneity of samples may also be a source of heterogeneity and may affect the accuracy of results. Further analysis is needed to use real-world data about the efficacy or effectiveness of COVID-19 vaccines.

In summary, the current COVID-19 vaccines could effectively protect COVID-19 caused by the Delta variant and relatively less effective for the Omicron variant. The waning immunity was observed especially in old people after full vaccination. Breakthrough infection seems rare within 6 months and is less likely to cause severe COVID-19 in young people after full vaccination against the Delta variant. Booster immunization could enhance protection capability. Moreover, two doses’ immunization could provide additional protection for severe COVID-19 against the Omicron variant.

## Data availability statement

The original contributions presented in the study are included in the article/**Supplementary Material**. Further inquiries can be directed to the corresponding author.

## Author contributions

Conceptualization: XH, ST. Data curation: XH, JS, YM, WZ. Formal analysis: JS, YM, WZ. Investigation: JS. Methodology: XH, YM, JS. Project administration: XH, JS, YM, WZ. Supervision: ST. Validation: XH, JS, ST. Writing—original draft: XH, ST. Writing—review and editing: XH, JS, YM, WZ, ST. All authors contributed to the article and approved the submitted version.

## Conflict of interest

The authors declare that the research was conducted in the absence of any commercial or financial relationships that could be construed as a potential conflict of interest.

## Publisher’s note

All claims expressed in this article are solely those of the authors and do not necessarily represent those of their affiliated organizations, or those of the publisher, the editors and the reviewers. Any product that may be evaluated in this article, or claim that may be made by its manufacturer, is not guaranteed or endorsed by the publisher.
