# Supplementary material for: A comprehensive analysis of the efficacy and effectiveness of COVID-19 vaccines
Source: Front Immunol. 2022 Aug 26;13:945930. doi: 10.3389/fimmu.2022.945930 (PMC9459021; doi:10.3389/fimmu.2022.945930)
Supplement: Supplementary file 2 [file Table_1.docx]

**Supplementary Table 1** The characteristics of included studies for the COVID-19 vaccines information

| **Vaccine type** | **Vaccine name** | **Developer** | **Dose schedule** | **References** |
| --- | --- | --- | --- | --- |
| Inactivated virus | CoronaVac | Sinovac Research and Development | 0 days: 3 ug; 14 days: 3 ug | Palacios R [44] 2021; Fadlyana E [42] 2021; Tanriover MD [38] 2021 |
| Inactivated virus | WIV04 | The Beijing Institute of Biological Products Co, Ltd | 0 days:4 µg; 21 days: 4 ug | Al Kaabi N [31] 2021 |
| Inactivated virus | HB02 | The Beijing Institute of Biological Products Co, Ltd | 0 days:4 µg; 21 days: 4 ug | Al Kaabi N [31] 2021 |
| Inactivated virus | BBV152 | Bharat Biotech International Hyderabad, India | 0 days: 6 ug; 28 days: 6 ug | Ella R [32] 2021 |
| Protein subunit | NVX-CoV2373 | Novavax | 0 days: 5 mg with Matrix-M1 adjuvant; 21 days: 5 mg with Matrix-M1 adjuvant | Dunkle LM [49] 2022; Heath PT [36] 2021; Shinde V [54] 2021 |
| Protein subunit | SCB-2019 | Clover Biopharmaceuticals, China | 0 days: 30 ug; 21 days: 30 ug | Bravo L [52] 2022 |
| RNA-based vaccine | BNT162b2 | Pfizer/BioNTech; Fosun Pharma | 0 days: 30 ug; 21 days: 30 ug | Frenck RW Jr [35] 2021; Polack FP [117] 2020; Thomas SJ [39] 2021; Walter EB [46] 2022 |
| RNA-based vaccine | mRNA-1273 | Moderna; National Institute of Allergy and Infectious Diseases (NIAID) | 0 days: 100 ug; 28 days: 100 ug | Ali K [33] 2021; Baden LR [116] 2020; El Sahly HM [41] 2021 |
| RNA-based vaccine candidate | CVnCoV | CureVac AG | 0 days: 12 ug; 28 days: 12 ug | Kremsner PG [45] 2022 |
| Viral vector (non-replicating) | AZD1222 (ChAdOx1 nCoV-19) | University of Oxford/AstraZeneca | 0 days: low dose (2.2×10^10^ virus particles)/standard dose (3.5–6.5×10^10^ virus particles); 28 days: standard dose (3.5–6.5×10^10^ virus particles) | Clemens SAC [43] 2021; Falsey AR [34] 2021; Madhi SA [40] 2021; Voysey M [29] 2021; Voysey M [47] 2021 |
| Viral vector (non-replicating) | Sputnik V | Gamaleya Research Institute; Health Ministry of the Russian Federation | 0 days: rAd26 10^11^ viral particles; 21 days: rAd5 10^11^ viral particles | Logunov DY [31] 2021 |
| Viral vector (non-replicating) | Ad26.COV2.S | Janssen/Johnson & Johnson | Single dose: 5×10^10^ viral particles | Sadoff J [37] 2021 |
| Viral vector (non-replicating) | Ad5-nCoV | CanSino Biologics, Tianjin, China | Single dose: 5×10^10^ viral particles | Halperin SA [50] 2022 |
