# Supplementary material for: A comprehensive analysis of the efficacy and effectiveness of COVID-19 vaccines
Source: Front Immunol. 2022 Aug 26;13:945930. doi: 10.3389/fimmu.2022.945930 (PMC9459021; doi:10.3389/fimmu.2022.945930)
Supplement: Supplementary file 3 [file Table_2.docx]

**Supplementary Table 2** Efficacy of included Phase III RCT studies for the SARS-CoV-2 infection and COVID-19

| **First author/year** | **Vaccine name** | **Times of confirmed cases** | **Country** | **Age (year)** | **Dose and times of confirmed cases** | **Type of cases** | **Subgroup** | **Vaccine** | | **Placebo** | | **VE % (95%CI)** |
| --- | --- | --- | --- | --- | --- | --- | --- | --- | --- | --- | --- | --- |
|  |  |  |  |  |  |  |  | **No. of events** | **Total no.** | **No. of events** | **Total no.** |  |
| Al Kaabi N [31] 2021 | HB02 | July 16, 2020 to December 20, 2020 | United Arab and Emirates | ≥18 | After dose 2 (≥ 14) | Asymptomatic infection | Overall | 10 | 12726 | 21 | 12817 | 52.0 (-1.9, 77.4) |
| Al Kaabi N [31] 2021 | HB02 | July 16, 2020 to December 20, 2020 | United Arab and Emirates | ≥18 | After dose 2 (≥ 14) | SARS-CoV-2 infection | Overall | 31 | 12726 | 116 | 12722 | 73.5 (60.6, 82.2) |
| Al Kaabi N [31] 2021 | HB02 | July 16, 2020 to December 20, 2020 | United Arab and Emirates | ≥18 | After dose 2 (≥ 14) | Severe COVID-19 | Overall | 0 | 12726 | 2 | 12737 | 100 |
| Al Kaabi N [31] 2021 | HB02 | July 16, 2020 to December 20, 2020 | United Arab and Emirates | ≥18 | After dose 2 (≥ 14) | Symptomatic COVID-19 | 18- 59 year | 21 | 12525 | 95 | 12539 | 78.1 (64.9, 86.4) |
| Al Kaabi N [31] 2021 | HB02 | July 16, 2020 to December 20, 2020 | United Arab and Emirates | ≥18 | After dose 2 (≥ 14) | Symptomatic COVID-19 | Female | 3 | 1976 | 12 | 1932 | 75.6 (9.5, 95.6) |
| Al Kaabi N [31] 2021 | HB02 | July 16, 2020 to December 20, 2020 | United Arab and Emirates | ≥18 | After dose 2 (≥ 14) | Symptomatic COVID-19 | Male | 18 | 10750 | 83 | 10805 | 78.4 (64.1, 87.0) |
| Al Kaabi N [31] 2021 | HB02 | July 16, 2020 to December 20, 2020 | United Arab and Emirates | ≥18 | After dose 2 (≥ 14) | Symptomatic COVID-19 | Overall | 21 | 12726 | 95 | 12737 | 78.1 (64.8, 86.3) |
| Al Kaabi N [31] 2021 | WIV04 | July 16, 2020 to December 20, 2020 | United Arab and Emirates | ≥18 | After dose 2 (≥ 14) | Asymptomatic infection | Overall | 16 | 12753 | 21 | 12817 | 23.4 (-46.7, 60.0) |
| Al Kaabi N [31] 2021 | WIV04 | July 16, 2020 to December 20, 2020 | United Arab and Emirates | ≥18 | After dose 2 (≥ 14) | SARS-CoV-2 infection | Overall | 42 | 12743 | 116 | 12722 | 64.0 (48.8, 74.7) |
| Al Kaabi N [31] 2021 | WIV04 | July 16, 2020 to December 20, 2020 | United Arab and Emirates | ≥18 | After dose 2 (≥ 14) | Severe COVID-19 | Overall | 0 | 12743 | 2 | 12737 | 100 |
| Al Kaabi N [31] 2021 | WIV04 | July 16, 2020 to December 20, 2020 | United Arab and Emirates | ≥18 | After dose 2 (≥ 14) | Symptomatic COVID-19 | 18- 60 year | 26 | 12530 | 95 | 12539 | 72.8 (58.0, 82.4) |
| Al Kaabi N [31] 2021 | WIV04 | July 16, 2020 to December 20, 2020 | United Arab and Emirates | ≥18 | After dose 2 (≥ 14) | Symptomatic COVID-19 | Female | 3 | 2037 | 12 | 1932 | 76.7 (13.5, 95.8) |
| Al Kaabi N [31] 2021 | WIV04 | July 16, 2020 to December 20, 2020 | United Arab and Emirates | ≥18 | After dose 2 (≥ 14) | Symptomatic COVID-19 | Male | 23 | 10706 | 83 | 10805 | 72.2 (55.8, 82.5) |
| Al Kaabi N [31] 2021 | WIV04 | July 16, 2020 to December 20, 2020 | United Arab and Emirates | ≥18 | After dose 2 (≥ 14) | Symptomatic COVID-19 | Overall | 26 | 12743 | 95 | 12737 | 72.8 (58.1, 82.4) |
| Ali K [33] 2021 | mRNA-1273 | December 9, 2020 to February 28, 2021 | USA | 12-17 | After dose 2 (≥ 14) | Asymptomatic infection | Overall | 21 | 2139 | 16 | 1042 | 39.2 (−24.7, 69.7) |
| Ali K [33] 2021 | mRNA-1273 | December 9, 2020 to February 28, 2021 | USA | 12-17 | After dose 2 (≥ 14) | SARS-CoV-2 infection | Overall | 22 | 2139 | 23 | 1042 | 55.7 (16.8, 76.4) |
| Ali K [33] 2021 | mRNA-1273 | December 9, 2020 to February 28, 2021 | USA | 12-17 | After dose 2 (≥ 14) | Symptomatic COVID-19 | Overall | 1 | 2139 | 7 | 1042 | 93.3 (47.9, 99.9) |
| Baden LR [116] 2020 | mRNA-1273 | July 27, 2020, and November 25, 2020 | USA | ≥18 | After dose 2 (≥ 14) | Severe COVID-19 | Overall | 0 | 14073 | 30 | 14073 | 100 |
| Baden LR [116] 2020 | mRNA-1273 | July 27, 2020, and November 25, 2020 | USA | ≥18 | After dose 2 (≥ 14) | Symptomatic COVID-19 | ≥65 year | 4 | 3583 | 29 | 3552 | 86.4 (61.4, 95.2) |
| Baden LR [116] 2020 | mRNA-1273 | July 27, 2020, and November 25, 2020 | USA | ≥18 | After dose 2 (≥ 14) | Symptomatic COVID-19 | 18- 64 year | 7 | 10551 | 156 | 10521 | 95.6 (90.6, 97.9) |
| Baden LR [116] 2020 | mRNA-1273 | July 27, 2020, and November 25, 2020 | USA | ≥18 | After dose 2 (≥ 14) | Symptomatic COVID-19 | Female | 7 | 6768 | 98 | 6611 | 93.1 (85.2, 96.8) |
| Baden LR [116] 2020 | mRNA-1273 | July 27, 2020, and November 25, 2020 | USA | ≥18 | After dose 2 (≥ 14) | Symptomatic COVID-19 | Male | 4 | 7366 | 87 | 7462 | 95.4 (87.4, 98.3) |
| Bravo L [52] 2022 | SCB-2019 | March 24, 2021 to Aug 10, 2021 | Multiple country | ≥18 | After dose 2 (≥ 14) | Symptomatic COVID-19 | ≥60 year | 3 | 121 | 8 | 127 | 58·4 (-73·4, 92·9) |
| Bravo L [52] 2022 | SCB-2019 | March 24, 2021 to Aug 10, 2021 | Multiple country | ≥18 | After dose 2 (≥ 14) | Symptomatic COVID-19 | 18−59 year | 49 | 5814 | 147 | 5679 | 67·5 (54·8, 77·0) |
| Bravo L [52] 2022 | SCB-2019 | March 24, 2021 to Aug 10, 2021 | Multiple country | ≥18 | After dose 2 (≥ 14) | Symptomatic COVID-19 | Female | 27 | 3232 | 83 | 3237 | 67·4 (49·1, 79·7) |
| Bravo L [52] 2022 | SCB-2019 | March 24, 2021 to Aug 10, 2021 | Multiple country | ≥18 | After dose 2 (≥ 14) | Symptomatic COVID-19 | Male | 25 | 2703 | 72 | 2569 | 67·1 (47·5, 80·0) |
| Bravo L [52] 2022 | SCB-2019 | March 24, 2021 to Aug 10, 2021 | Multiple country | ≥18 | After dose 2 (≥ 14) | Symptomatic COVID-19 | Overall | 52 | 5935 | 155 | 5806 | 67·2 (54·3, 76·8) |
| Bravo L [52] 2022 | SCB-2019 | March 24, 2021 to Aug 10, 2021 | Multiple country | ≥18 | After dose 2 (≥ 14) | Severe COVID-19 | Overall | 0 | 5935 | 8 | 5806 | 100 |
| Palacios R [44] 2021 | CoronaVac | July 21, 2020 and Dec 16, 2020 | Brazil | ≥18 | After dose 2 (≥ 14) | Symptomatic COVID-19 | ≥60 year | 2 | 212 | 4 | 207 | 51.1 (-166.9, 91.0) |
| Palacios R [44] 2021 | CoronaVac | July 21, 2020 and Dec 16, 2020 | Brazil | ≥18 | After dose 2 (≥ 14) | Symptomatic COVID-19 | 18-59 year | 83 | 4741 | 164 | 4663 | 50.7 (35.8, 62.1) |
| Palacios R [44] 2021 | CoronaVac | July 21, 2020 and Dec 16, 2020 | Brazil | ≥18 | After dose 2 (≥ 14) | Severe COVID-19 | Overall | 0 | 4953 | 6 | 4870 | 100·0(16·9, 100·0) |
| Palacios R [44] 2021 | CoronaVac | July 21, 2020 and Dec 16, 2020 | Brazil | ≥18 | After dose 2 (≥ 14) | Symptomatic COVID-19 | Overall | 85 | 4953 | 168 | 4870 | 50.7 (35.7, 62.2) |
| Clemens SAC [43] 2021 | ChAdOx1 nCoV-19 | June 23, 2020 to December 1, 2020. | Brazil | ≥18 | After dose 2 (≥ 14) | Severe COVID-19 | Overall | 1 | 4772 | 18 | 4661 | 95 (61, 99) |
| Clemens SAC [43] 2021 | ChAdOx1 nCoV-19 | June 23, 2020 to December 1, 2020. | Brazil | ≥18 | After dose 2 (≥ 14) | Symptomatic COVID-19 | Overall | 77 | 4772 | 222 | 4661 | 65.1 (54.9, 73.0) |
| Dunkle LM [49] 2022 | NVX-CoV2373 | December 27, 2020, to February 18, 2021 | USA and Mexico | ≥18 | After dose 2 (≥ 7) | Severe COVID-19 | Overall | 0 | 17312 | 4 | 8140 | 100 |
| Dunkle LM [49] 2022 | NVX-CoV2373 | December 27, 2020, to February 18, 2021 | USA and Mexico | ≥18 | After dose 2 (≥ 7) | Symptomatic COVID-19 | ≥65 year | 2 | 2048 | 2 | 946 | 54.8 (-228, 94) |
| Dunkle LM [49] 2022 | NVX-CoV2373 | December 27, 2020, to February 18, 2021 | USA and Mexico | ≥18 | After dose 2 (≥ 7) | Symptomatic COVID-19 | 18- 64 year | 12 | 15264 | 61 | 7194 | 91.5 (84.2, 95.4) |
| Dunkle LM [49] 2022 | NVX-CoV2373 | December 27, 2020, to February 18, 2021 | USA and Mexico | ≥18 | After dose 2 (≥ 7) | Symptomatic COVID-19 | Female | 9 | 8262 | 40 | 4009 | 90.0 (79.3, 95.1) |
| Dunkle LM [49] 2022 | NVX-CoV2373 | December 27, 2020, to February 18, 2021 | USA and Mexico | ≥18 | After dose 2 (≥ 7) | Symptomatic COVID-19 | Male | 5 | 9050 | 23 | 4131 | 90.9 (76.0, 96.5) |
| Dunkle LM [49] 2022 | NVX-CoV2373 | December 27, 2020, to February 18, 2021 | USA and Mexico | ≥18 | After dose 2 (≥ 7) | Symptomatic COVID-19 | Overall | 14 | 17312 | 63 | 8140 | 90.4 (82.9, 94.6) |
| El Sahly HM [41] 2021 | mRNA-1273 | From July 27 2020 to March 26, 2021 | USA | ≥18 | After dose 2 (≥ 14) | Asymptomatic infection | Overall | 214 | 14287 | 498 | 14164 | 63.0 (56.6–68.5) |
| El Sahly HM [41] 2021 | mRNA-1273 | From July 27 2020 to March 26, 2021 | USA | ≥18 | After dose 2 (≥ 14) | SARS-CoV-2 infection | Overall | 280 | 14287 | 1339 | 14164 | 82.0 (79.5–84.2) |
| El Sahly HM [41] 2021 | mRNA-1273 | From July 27 2020 to March 26, 2021 | USA | ≥18 | After dose 2 (≥ 14) | Severe COVID-19 | Overall | 2 | 14287 | 106 | 14164 | 98.2 (92.8–99.6) |
| El Sahly HM [41] 2021 | mRNA-1273 | From July 27 2020 to March 26, 2021 | USA | ≥18 | After dose 2 (≥ 14) | COVID-19-related death | Overall | 0 | 14287 | 3 | 14164 | 100.0 (NE, 100.0) |
| El Sahly HM [41] 2021 | mRNA-1273 | From July 27 2020 to March 26, 2021 | USA | ≥18 | After dose 2 (≥ 14) | Symptomatic COVID-19 | ≥65 year | 9 | 3626 | 100 | 3595 | 91.5 (83.2, 95.7) |
| El Sahly HM [41] 2021 | mRNA-1273 | From July 27 2020 to March 26, 2021 | USA | ≥18 | After dose 2 (≥ 14) | Symptomatic COVID-19 | ≥75 year | 0 | 636 | 19 | 697 | 100.0 (NE, 100.0) |
| El Sahly HM [41] 2021 | mRNA-1273 | From July 27 2020 to March 26, 2021 | USA | ≥18 | After dose 2 (≥ 14) | Symptomatic COVID-19 | 18-65 year | 46 | 10661 | 644 | 10569 | 93.4 (91.1, 95.1) |
| El Sahly HM [41] 2021 | mRNA-1273 | From July 27 2020 to March 26, 2021 | USA | ≥18 | After dose 2 (≥ 14) | Symptomatic COVID-19 | 65-75 year | 9 | 2990 | 81 | 2898 | 89.7 (79.6, 94.9) |
| El Sahly HM [41] 2021 | mRNA-1273 | From July 27 2020 to March 26, 2021 | USA | ≥18 | After dose 2 (≥ 14) | Symptomatic COVID-19 | Female | 25 | 6848 | 366 | 6670 | 93.8 (90.7, 95.9) |
| El Sahly HM [41] 2021 | mRNA-1273 | From July 27 2020 to March 26, 2021 | USA | ≥18 | After dose 2 (≥ 14) | Symptomatic COVID-19 | Male | 30 | 7439 | 378 | 7494 | 92.5 (89.1, 94.8) |
| El Sahly HM [41] 2021 | mRNA-1273 | From July 27 2020 to March 26, 2021 | USA | ≥18 | After dose 2 (≥ 14) | Symptomatic COVID-19 | Overall | 55 | 14287 | 744 | 14164 | 93.2 (91.0, 94.8) |
| Ella R [32] 2021 | BBV152 | November 16, 2020 to May 17, 2021 | Indian | ≥18 | After dose 2 (≥ 14) | Asymptomatic infection | Overall | 13 | 3248 | 33 | 3041 | 63·6 (29·0, 82·4) |
| Ella R [32] 2021 | BBV152 | November 16, 2020 to May 17, 2021 | Indian | ≥18 | After dose 2 (≥ 14) | SARS-CoV-2 infection | Overall | 19 | 3248 | 56 | 3041 | 68·8 (46·7, 82·5) |
| Ella R [32] 2021 | BBV152 | November 16, 2020 to May 17, 2021 | Indian | ≥18 | After dose 2 (≥ 14) | Severe COVID-19 | Overall | 1 | 8471 | 15 | 8502 | 93·4 (57·1, 99·8) |
| Ella R [32] 2021 | BBV152 | November 16, 2020 to May 17, 2021 | Indian | ≥18 | After dose 2 (≥ 14) | Symptomatic COVID-19 | ≥60 | 5 | 893 | 16 | 965 | 67·8 (8·0, 90·0) |
| Ella R [32] 2021 | BBV152 | November 16, 2020 to May 17, 2021 | Indian | ≥18 | After dose 2 (≥ 14) | Symptomatic COVID-19 | 18- 59 year | 19 | 7578 | 90 | 7537 | 79·4 (66·0, 88·2) |
| Ella R [32] 2021 | BBV152 | November 16, 2020 to May 17, 2021 | Indian | ≥18 | After dose 2 (≥ 14) | Symptomatic COVID-19 | Overall | 24 | 8471 | 106 | 8502 | 77·8 (65·2, 86·4) |
| Fadlyana E [42] 2021 | CoronaVac | August 11, 2020 to October 21, 2020 | Indonesia | ≥18 | After dose 2 (≥ 14) | Symptomatic COVID-19 | Overall | 7 | 811 | 18 | 809 | 60.7 (6.4, 83.5) |
| Falsey AR [34] 2021 | ChAdOx1 nCoV-19 | August 28, 2020 to January 15, 2021 | United States, Chile, and Peru | ≥18 | After dose 2 (≥ 14) | Severe COVID-19 | Overall | 0 | 17762 | 8 | 8550 | 100.0 (71.6, NE) |
| Falsey AR [34] 2021 | ChAdOx1 nCoV-19 | August 28, 2020 to January 15, 2021 | United States, Chile, and Peru | ≥18 | After dose 2 (≥ 14) | Symptomatic COVID-19 | ≥65 year | 5 | 3696 | 14 | 1812 | 83.5 (54.2, 94.1) |
| Falsey AR [34] 2021 | ChAdOx1 nCoV-19 | August 28, 2020 to January 15, 2021 | United States, Chile, and Peru | ≥18 | After dose 2 (≥ 14) | Symptomatic COVID-19 | 18-65 year | 68 | 13966 | 116 | 6738 | 72.8 (63.4, 79.9) |
| Falsey AR [34] 2021 | ChAdOx1 nCoV-19 | August 28, 2020 to January 15, 2021 | United States, Chile, and Peru | ≥18 | After dose 2 (≥ 14) | Symptomatic COVID-19 | Female | 32 | 7740 | 46 | 3721 | 68.2 (50.0, 79.7) |
| Falsey AR [34] 2021 | ChAdOx1 nCoV-19 | August 28, 2020 to January 15, 2021 | United States, Chile, and Peru | ≥18 | After dose 2 (≥ 14) | Symptomatic COVID-19 | Male | 41 | 9922 | 84 | 4829 | 77.2 (66.9, 84.3) |
| Falsey AR [34] 2021 | ChAdOx1 nCoV-19 | August 28, 2020 to January 15, 2021 | United States, Chile, and Peru | ≥18 | After dose 2 (≥ 14) | Symptomatic COVID-19 | Overall | 73 | 17662 | 130 | 8550 | 74.0 (65.3, 80.5) |
| Frenck RW Jr [35] 2021 | BNT162b2 | October 15, 2020 to January 12, 2021 | USA | 12-15 | After dose 2 (≥ 7) | Symptomatic COVID-19 | Overall | 0 | 1005 | 16 | 978 | 100 (75.3, 100) |
| Halperin SA [50] 2022 | Ad5-nCoV | Sept 22, 2020 to Jan 15, 2021 | Multiple country | ≥18 | After single dose (≥ 14) | Severe COVID-19 | Overall | 1 | 14591 | 25 | 14586 | 96.0 (70.4, 99.5) |
| Halperin SA [50] 2022 | Ad5-nCoV | Sept 22, 2020 to Jan 15, 2021 | Multiple country | ≥18 | After single dose (≥ 14) | Symptomatic COVID-19 | ≥60 year | 10 | 1323 | 21 | 1347 | 53·3 (0·9, 78·0) |
| Halperin SA [50] 2022 | Ad5-nCoV | Sept 22, 2020 to Jan 15, 2021 | Multiple country | ≥18 | After single dose (≥ 14) | Symptomatic COVID-19 | 18–44 year | 49 | 10102 | 143 | 10114 | 65·8 (52·7, 75·3) |
| Halperin SA [50] 2022 | Ad5-nCoV | Sept 22, 2020 to Jan 15, 2021 | Multiple country | ≥18 | After single dose (≥ 14) | Symptomatic COVID-19 | 45–59 year | 18 | 3166 | 47 | 3125 | 62·7 (35·8, 78·4) |
| Halperin SA [50] 2022 | Ad5-nCoV | Sept 22, 2020 to Jan 15, 2021 | Multiple country | ≥18 | After single dose (≥ 14) | Symptomatic COVID-19 | Female | 34 | 4794 | 73 | 4577 | 55·7 (33·5, 70·5) |
| Halperin SA [50] 2022 | Ad5-nCoV | Sept 22, 2020 to Jan 15, 2021 | Multiple country | ≥18 | After single dose (≥ 14) | Symptomatic COVID-19 | Male | 43 | 9797 | 138 | 10009 | 68·5 (55·7, 77·7) |
| Heath PT [36] 2021 | NVX-CoV2373 | Sept 28, 2020 to Nov 28, 2020 | UK and South Africa | 18-84 | After dose 2 (≥ 7) | Severe COVID-19 | Overall | 0 | 7020 | 5 | 7019 | 100 |
| Heath PT [36] 2021 | NVX-CoV2373 | Sept 28, 2020 to Nov 28, 2020 | UK and South Africa | 18-84 | After dose 2 (≥ 7) | Symptomatic COVID-19 | ≥65 year | 1 | 1953 | 9 | 1957 | 88.9 (20.2, 99.7) |
| Heath PT [36] 2021 | NVX-CoV2373 | Sept 28, 2020 to Nov 28, 2020 | UK and South Africa | 18-84 | After dose 2 (≥ 7) | Symptomatic COVID-19 | 18-65 year | 9 | 5067 | 87 | 5062 | 89.8 (79.7, 95.5) |
| Heath PT [36] 2021 | NVX-CoV2373 | Sept 28, 2020 to Nov 28, 2020 | UK and South Africa | 18-84 | After dose 2 (≥ 7) | Symptomatic COVID-19 | Overall | 10 | 7020 | 96 | 7019 | 89.7 (80.2, 94.6) |
| Kremsner PG [45] 2022 | CVnCoV | December 11, 2020 to April 12, 2020 | Multiple country | ≥18 | After dose 2 (≥ 14) | Severe COVID-19 | ≥61 year | 2 | 1319 | 1 | 1180 | -78.8 (-80.3, 83.8) |
| Kremsner PG [45] 2022 | CVnCoV | December 11, 2020 to April 12, 2020 | Multiple country | ≥18 | After dose 2 (≥ 14) | Severe COVID-19 | 18- 60 year | 2 | 11532 | 9 | 11031 | 78.7 (1.6, 95.4) |
| Kremsner PG [45] 2022 | CVnCoV | December 11, 2020 to April 12, 2020 | Multiple country | ≥18 | After dose 2 (≥ 14) | Severe COVID-19 | Overall | 4 | 12851 | 10 | 12211 | 62.0 (-21.2, 78.1) |
| Kremsner PG [45] 2022 | CVnCoV | December 11, 2020 to April 12, 2020 | Multiple country | ≥18 | After dose 2 (≥ 14) | Symptomatic COVID-19 | ≥61 year | 12 | 1319 | 9 | 1180 | -19.1 (-181.7, 49.6) |
| Kremsner PG [45] 2022 | CVnCoV | December 11, 2020 to April 12, 2020 | Multiple country | ≥18 | After dose 2 (≥ 14) | Symptomatic COVID-19 | 18- 60 year | 71 | 11532 | 136 | 11031 | 52·5 (36·2, 64·8) |
| Kremsner PG [45] 2022 | CVnCoV | December 11, 2020 to April 12, 2020 | Multiple country | ≥18 | After dose 2 (≥ 14) | Symptomatic COVID-19 | Overall | 83 | 12851 | 145 | 12211 | 48·2 (31·0, 61·4) |
| Logunov DY [31] 2021 | Sputnik V | September 7, 2020 to November 24, 2020 | Russia | ≥18 | After dose 1 (≥ 21) | Symptomatic COVID-19 | Overall | 13 | 14094 | 47 | 4601 | 91·1 (83·8, 95·1) |
| Madhi SA [40] 2021 | ChAdOx1 nCoV-19 | June 24, 2020 to November 9, 2020 | South Africa | 18-65 | After dose 2 (≥ 14) | Symptomatic COVID-19 | Overall | 19 | 750 | 23 | 717 | 21.9 (-49.9, 59.8) |
| Polack FP [117] 2020 | BNT162b2 | July 27, 2020, and November 14, 2020 | Multiple country | ≥ 16 | After dose 2 (≥ 7) | Symptomatic COVID-19 | >55 year | 3 | 7500 | 48 | 7543 | 93.7 (80.6, 98.8) |
| Polack FP [117] 2020 | BNT162b2 | July 27, 2020, and November 14, 2020 | Multiple country | ≥ 16 | After dose 2 (≥ 7) | Symptomatic COVID-19 | ≥65 year | 1 | 3848 | 19 | 3880 | 94.7 (66.7, 99.9 |
| Polack FP [117] 2020 | BNT162b2 | July 27, 2020, and November 14, 2020 | Multiple country | ≥ 16 | After dose 2 (≥ 7) | Symptomatic COVID-19 | ≥75 year | 0 | 774 | 5 | 785 | 100.0 |
| Polack FP [117] 2020 | BNT162b2 | July 27, 2020, and November 14, 2020 | Multiple country | ≥ 16 | After dose 2 (≥ 7) | Symptomatic COVID-19 | 16-55 year | 5 | 9897 | 114 | 9955 | 95.6 (89.4, 98.6) |
| Polack FP [117] 2020 | BNT162b2 | July 27, 2020, and November 14, 2020 | Multiple country | ≥ 16 | After dose 2 (≥ 7) | Symptomatic COVID-19 | Female | 5 | 8536 | 81 | 8749 | 93.7 (84.7, 98.0) |
| Polack FP [117] 2020 | BNT162b2 | July 27, 2020, and November 14, 2020 | Multiple country | ≥ 16 | After dose 2 (≥ 7) | Symptomatic COVID-19 | Male | 3 | 8875 | 81 | 8762 | 96.4 (88.9, 99.3) |
| Polack FP [117] 2020 | BNT162b2 | July 27, 2020, and November 14, 2020 | Multiple country | ≥ 16 | After dose 2 (≥ 7) | Symptomatic COVID-19 | Overall | 8 | 17411 | 162 | 17511 | 95.0 (90.3, 97.6) |
| Sadoff J [37] 2021 | Ad26.COV2.S | September 21, 2020 to January 22, 2021 | Multiple country | ≥18 | After single dose (≥ 14) | Asymptomatic infection | Overall | 18 | 19306 | 50 | 19178 | 65.5 (39.9, 81.1) |
| Sadoff J [37] 2021 | Ad26.COV2.S | September 21, 2020 to January 22, 2021 | Multiple country | ≥18 | After single dose (≥ 14) | Severe COVID-19 | Overall | 14 | 19514 | 60 | 19544 | 76.7 (54.6, 89.1) |
| Sadoff J [37] 2021 | Ad26.COV2.S | September 21, 2020 to January 22, 2021 | Multiple country | ≥18 | After single dose (≥ 14) | Symptomatic COVID-19 | ≥60 year | 22 | 19514 | 91 | 19544 | 76.3 (61.6, 86.0) |
| Sadoff J [37] 2021 | Ad26.COV2.S | September 21, 2020 to January 22, 2021 | Multiple country | ≥18 | After single dose (≥ 14) | Symptomatic COVID-19 | 18−59 year | 95 | 19514 | 260 | 19544 | 63.7 (53.9, 71.6) |
| Sadoff J [37] 2021 | Ad26.COV2.S | September 21, 2020 to January 22, 2021 | Multiple country | ≥18 | After single dose (≥ 14) | Symptomatic COVID-19 | Female | 88 | 8649 | 240 | 8708 | 63.4 (53.1, 71.7) |
| Sadoff J [37] 2021 | Ad26.COV2.S | September 21, 2020 to January 22, 2021 | Multiple country | ≥18 | After single dose (≥ 14) | Symptomatic COVID-19 | Male | 85 | 10861 | 269 | 10832 | 68.8 (60.1, 75.9) |
| Sadoff J [37] 2021 | Ad26.COV2.S | September 21, 2020 to January 22, 2021 | Multiple country | ≥18 | After single dose (≥ 14) | Symptomatic COVID-19 | Overall | 117 | 19514 | 351 | 19544 | 66.9 (59.1, 73.4) |
| Sadoff J [53] 2022 | Ad26.COV2.S | September 21, 2020 to July 9, 2021 | Multiple country | ≥18 | After single dose (≥ 14) | Severe COVID-19 | Overall | 56 | 19400 | 205 | 19398 | 73.3 (63.9, 80.5) |
| Sadoff J [53] 2022 | Ad26.COV2.S | September 21, 2020 to July 9, 2021 | Multiple country | ≥18 | After single dose (≥ 14) | Symptomatic COVID-19 | Overall | 495 | 19400 | 1082 | 19398 | 55.9 (51.0, 60.5) |
| Shinde V [54] 2021 | NVX-CoV2373 | August 17, 2020 to December 30, 2020 | South Africa | 18-84 | After dose 2 (≥ 7) | Symptomatic COVID-19 | Overall | 15 | 1357 | 29 | 1327 | 49.4 (6.1, 72.8) |
| Tanriover MD [38] 2021 | CoronaVac | Sept 14, 2020 to Jan 5, 2021 | Turkey | ≥18 | After dose 2 (≥ 7) | Severe COVID-19 | Overall | 0 | 6646 | 30 | 3,568 | 100 (20.4, 100) |
| Tanriover MD [38] 2021 | CoronaVac | Sept 14, 2020 to Jan 5, 2021 | Turkey | ≥18 | After dose 2 (≥ 14) | Symptomatic COVID-19 | Overall | 9 | 6646 | 32 | 3568 | 83.5 (65.4, 92.1) |
| Thomas SJ [39] 2021 | BNT162b2 | July 27, 2020 to March 13, 2021 | Multiple country | ≥16 | After dose 2 (≥ 7) | Symptomatic COVID-19 | >55 year | 25 | 8194 | 266 | 8208 | 90.9 (86.3, 94.2) |
| Thomas SJ [39] 2021 | BNT162b2 | July 27, 2020 to March 13, 2021 | Multiple country | ≥ 16 | After dose 2 (≥ 7) | Symptomatic COVID-19 | ≥65 year | 7 | 4192 | 124 | 4226 | 94.5 (88.3, 97.8) |
| Thomas SJ [39] 2021 | BNT162b2 | July 27, 2020 to March 13, 2021 | Multiple country | ≥ 16 | After dose 2 (≥ 7) | Symptomatic COVID-19 | ≥75 year | 1 | 842 | 26 | 847 | 96.2 (76.9, 99.9) |
| Thomas SJ [39] 2021 | BNT162b2 | July 27, 2020 to March 13, 2021 | Multiple country | ≥ 16 | After dose 2 (≥ 7) | Symptomatic COVID-19 | 16-17 year | 0 | 342 | 10 | 331 | 100 (58.2, 100) |
| Thomas SJ [39] 2021 | BNT162b2 | July 27, 2020 to March 13, 2021 | Multiple country | ≥ 16 | After dose 2 (≥ 7) | Symptomatic COVID-19 | 16-55 year | 52 | 11517 | 568 | 11533 | 91.2 (88.3, 93.5) |
| Thomas SJ [39] 2021 | BNT162b2 | July 27, 2020 to March 13, 2021 | Multiple country | ≥ 16 | After dose 2 (≥ 7) | Symptomatic COVID-19 | Female | 35 | 10075 | 451 | 10280 | 92.4 (89.2, 94.7) |
| Thomas SJ [39] 2021 | BNT162b2 | July 27, 2020 to March 13, 2021 | Multiple country | ≥ 16 | After dose 2 (≥ 7) | Symptomatic COVID-19 | Male | 42 | 10637 | 399 | 10433 | 90.1 (86.4, 93.0) |
| Thomas SJ [39] 2021 | BNT162b2 | July 27, 2020 to March 13, 2021 | Multiple country | ≥ 16 | After dose 2 (≥ 7) | Symptomatic COVID-19 | Overall | 77 | 20712 | 850 | 20713 | 91.3 (89.0, 93.2) |
| Thomas SJ [56] 2022 | BNT162b2 | July 27, 2020 to March 13, 2021 | Multiple country | ≥12 | After dose 2 (≥ 7) | Symptomatic COVID-19 | Cancer | 4 | 20712 | 71 | 20713 | 94.4 (85.1, 98.5) |
| Voysey M [29] 2021 | ChAdOx1 nCoV-19 | April 23. 2020 and Dec 6, 2020 | UK | ≥18 | After dose 2 (≥ 14) | Asymptomatic infection | Overall | 57 | 4071 | 73 | 4136 | 22·2 (–9·9, 45·0) |
| Voysey M [29] 2021 | ChAdOx1 nCoV-19 | April 23. 2020 and Dec 6, 2020 | UK, Brazil, and South Africa | ≥18 | After dose 2 (≥ 14) | SARS-CoV-2 infection | Overall | 161 | 8597 | 346 | 8581 | 54·1 (44·7, 61·9) |
| Voysey M [29] 2021 | ChAdOx1 nCoV-19 | April 23. 2020 and Dec 6, 2020 | UK, Brazil, and South Africa | ≥18 | After dose 2 (≥ 14) | Symptomatic COVID-19 | Overall | 84 | 8597 | 248 | 8581 | 66·7 (57·4, 74·0) |
| Voysey M [47] 2021 | ChAdOx1 nCoV-19 | April 23, 2020 to Nov 4, 2020 | UK, Brazil, and South Africa | ≥18 | After dose 2 (≥ 14) | Asymptomatic infection | Overall | 29 | 3288 | 40 | 3350 | 27·3 (−17·2, 54·9) |
| Voysey M [47] 2021 | ChAdOx1 nCoV-19 | April 23, 2020 to Nov 4, 2020 | UK, Brazil, and South Africa | ≥18 | After dose 2 (≥ 14) | SARS-CoV-2 infection | Overall | 68 | 5807 | 153 | 5829 | 55·7 (41·1, 66·7) |
| Voysey M [47] 2021 | ChAdOx1 nCoV-19 | April 23, 2020 to Nov 4, 2020 | UK, Brazil, and South Africa | ≥18 | After dose 2 (≥ 14) | Symptomatic COVID-19 | Overall | 30 | 5807 | 101 | 5829 | 70·4 (54·8 to 80·6) |
| Walter EB [46] 2022 | BNT162b2 | June 7, 2021 to September 6, 2021 | USA | 5-11 | After dose 2 (≥ 7) | Symptomatic COVID-19 | Overall | 3 | 1305 | 16 | 663 | 90.7 (67.7, 98.3) |

**Full vaccination as ≥7 days after the second dose vaccination for COVID-19 vaccines BNT162b2 and NVX-CoV2373; ≥14 days for COVID-19 vaccines BNT162b2, mRNA-1273, ChAdOx1 nCoV-19, CoronaVac, BBV152, CVnCoV, WIV04, Sputnik V, HB02, SCB-2019; or after the single dose vaccination for COVID-19 vaccines Ad26.COV2.S; ≥21 days after the first dose vaccination for COVID-19 vaccine Sputnik V.**
