# Supplementary material for: A comprehensive analysis of the efficacy and effectiveness of COVID-19 vaccines
Source: Front Immunol. 2022 Aug 26;13:945930. doi: 10.3389/fimmu.2022.945930 (PMC9459021; doi:10.3389/fimmu.2022.945930)
Supplement: Supplementary file 4 [file Table_3.docx]

**Supplementary Table 3.** Efficacy or effectiveness of included studies during the SARS-CoV-2 Delta and Omicron variants period

| **First author** | **Vaccine name** | **Time interval since final dose (days)** | **Type of study** | **Age (year)** | **Type of cases** | **During variant Period/Variant** | **Country** | **Adjusted VE % (95%CI)** | **Adjusted RR or OR (95% CI)** |
| --- | --- | --- | --- | --- | --- | --- | --- | --- | --- |
| Andeweg SP [75] 2022 | BNT162b2, mRNA-1273, or Ad26.COV2.S | After full vaccination (≥ 14) | Case-control study | > 0 | SARS-CoV-2 infection | Delta (B.1.617.2) | The Netherlands | 76 (75, 76) | 0.24 (0.24, 0.25) |
| Andeweg SP [75] 2022 | BNT162b2, mRNA-1273, or Ad26.COV2.S | After full vaccination (≥ 14) | Case-control study | > 0 | SARS-CoV-2 infection | Omicron (B.1.1.529) | The Netherlands | 33 (31, 35) | 0.67 (0.65, 0.69) |
| Andrews N [105] 2021 | ChAdOx1 nCoV-19 | After dose 2 (≥ 14) | Case-control study | ≥65 | COVID-19-related death | Delta (B.1.617.2) | UK | 89 (78, 94) | 0.11 (0.06, 0.22) |
| Andrews N [105] 2021 | ChAdOx1 nCoV-19 | After dose 2 (≥ 14) | Case-control study | ≥16 | COVID-19-related death | Delta (B.1.617.2) | UK | 89 (80, 93) | 0.11 (0.07, 0.20) |
| Andrews N [105] 2021 | BNT162b2 | After dose 2 (≥ 14) | Case-control study | ≥65 | COVID-19-related death | Delta (B.1.617.2) | UK | 94 (90, 97) | 0.06 (0.03, 0.10) |
| Andrews N [105] 2021 | BNT162b2 | After dose 2 (≥ 14) | Case-control study | ≥16 | COVID-19-related death | Delta (B.1.617.2) | UK | 94 (91, 96) | 0.06 (0.04, 0.09) |
| Andrews N [105] 2021 | ChAdOx1 nCoV-19 | After dose 2 (≥ 14) | Case-control study | ≥65 | Severe COVID-19 | Delta (B.1.617.2) | UK | 86 (81, 90) | 0.14 (0.10, 0.19) |
| Andrews N [105] 2021 | ChAdOx1 nCoV-19 | After dose 2 (≥ 14) | Case-control study | ≥16 | Severe COVID-19 | Delta (B.1.617.2) | UK | 88 (85, 90) | 0.12 (0.10, 0.15) |
| Andrews N [105] 2021 | BNT162b2 | After dose 2 (≥ 14) | Case-control study | ≥65 | Severe COVID-19 | Delta (B.1.617.2) | UK | 94 (92, 96) | 0.06 (0.04, 0.08) |
| Andrews N [105] 2021 | BNT162b2 | After dose 2 (≥ 14) | Case-control study | ≥16 | Severe COVID-19 | Delta (B.1.617.2) | UK | 96 (94, 97) | 0.04 (0.03, 0.06) |
| Andrews N [105] 2021 | ChAdOx1 nCoV-19 | After dose 2 (≥ 14) | Case-control study | ≥65 | Symptomatic COVID-19 | Delta (B.1.617.2) | UK | 47 (42, 52) | 0.53 (0.48, 0.58) |
| Andrews N [105] 2021 | ChAdOx1 nCoV-19 | After dose 2 (≥ 14) | Case-control study | ≥16 | Symptomatic COVID-19 | Delta (B.1.617.2) | UK | 57 (56, 58) | 0.43 (0.42, 0.44) |
| Andrews N [105] 2021 | BNT162b2 | After dose 2 (≥ 14) | Case-control study | ≥65 | Symptomatic COVID-19 | Delta (B.1.617.2) | UK | 67 (63, 70) | 0.33 (0.30, 0.37) |
| Andrews N [105] 2021 | BNT162b2 | After dose 2 (≥ 14) | Case-control study | ≥16 | Symptomatic COVID-19 | Delta (B.1.617.2) | UK | 78 (77, 79) | 0.22 (0.21, 0.23) |
| Andrews N [105] 2021 | mRNA-1273 | After dose 2 (≥ 14) | Case-control study | ≥16 | Symptomatic COVID-19 | Delta (B.1.617.2) | UK | 92 (81, 96) | 0.08 (0.04, 0.19) |
| Bravo L [52] 2022 | SCB-2019 | After dose 2 (≥ 14) | RCT | ≥18 | Symptomatic COVID-19 | Delta (B.1.617.2) | Multiple | 79 (57, 90) | 0.21 (0.10, 0.43) |
| Bruxvoort KJ [66] 2021 | mRNA-1273 | After dose 2 (≥ 14) | Case-control study | ≥18 | SARS-CoV-2 infection | Delta (B.1.617.2) | USA | 87 (84, 89) | 0.13 (0.11, 0.16) |
| Bruxvoort KJ [66] 2021 | mRNA-1273 | After dose 2 (≥ 14) | Case-control study | ≥18 | Severe COVID-19 | Delta (B.1.617.2) | USA | 98 (93, 99) | 0.02 (0.01, 0.07) |
| Chin ET [69] 2021 | mRNA-1273 | After dose 2 (≥ 14) | Cohort study | > 0 | SARS-CoV-2 infection | Delta (B.1.617.2) | USA | 57 (42, 68) | 0.43 (0.32, 0.58) |
| Chin ET [69] 2021 | mRNA-1273 | After dose 2 (≥ 14) | Cohort study | > 0 | Symptomatic COVID-19 | Delta (B.1.617.2) | USA | 84 (56, 94) | 0.16 (0.06, 0.44) |
| Collie S [59] 2022 | BNT162b2 | After dose 2 (≥ 14) | Case-control study | > 0 | SARS-CoV-2 infection | Omicron (B.1.1.529) | South Africa | 69 (48–81) | 0.31 (0.19, 0.52) |
| Collie S [59] 2022 | BNT162b2 | After dose 2 (≥ 14) | Case-control study | > 0 | Severe COVID-19 | Omicron (B.1.1.529) | South Africa | 70 (62, 76) | 0.30 (0.24, 0.38) |
| Collie S [59] 2022 | BNT162b2 | After dose 2 (≥ 14) | Case-control study | > 0 | Symptomatic COVID-19 | Omicron (B.1.1.529) | South Africa | 50 (35, 62) | 0.50 (0.38, 0.65) |
| Dorabawila V [68] 2022 | BNT162b2 | After dose 2 (≥ 14) | Cohort study | 5-11 | SARS-CoV-2 infection | Omicron (B.1.1.529) | USA | 41 (38, 44) | 0.59 (0.56, 0.62) |
| Dorabawila V [68] 2022 | BNT162b2 | After dose 2 (≥ 14) | Cohort study | 12-17 | SARS-CoV-2 infection | Omicron (B.1.1.529) | USA | 61 (59, 62) | 0.39 (0.38, 0.41) |
| Dorabawila V [68] 2022 | BNT162b2 | After dose 2 (≥ 14) | Cohort study | 5-11 | Severe COVID-19 | Omicron (B.1.1.529) | USA | 70 (-16, 90) | 0.30 (0.10, 1.16) |
| Dorabawila V [68] 2022 | BNT162b2 | After dose 2 (≥ 14) | Cohort study | 12-17 | Severe COVID-19 | Omicron (B.1.1.529) | USA | 80 (63, 89) | 0.20 (0.11, 0.37) |
| Ella R [32] 2021 | BBV152 | After dose 2 (≥ 14) | RCT | ≥18 | Symptomatic COVID-19 | Delta (B.1.617.2) | Indian | 65 (33, 83) | 0.35 (0.17, 0.67) |
| Fabiani M [78] 2022 | BNT162b2 or mRNA-1273 | After dose 2 (≥ 14) | Cohort study | ≥80 | SARS-CoV-2 infection | Delta (B.1.617.2) | Italy | 35 (17, 49) | 0.65 (0.51, 0.83) |
| Fabiani M [78] 2022 | BNT162b2 or mRNA-1273 | After dose 2 (≥ 14) | Cohort study | 60-79 | SARS-CoV-2 infection | Delta (B.1.617.2) | Italy | 56 (51, 61) | 0.44 (0.39, 0.49) |
| Fabiani M [78] 2022 | BNT162b2 or mRNA-1273 | After dose 2 (≥ 14) | Cohort study | ≥16 | SARS-CoV-2 infection | Delta (B.1.617.2) | Italy | 69 (67, 71) | 0.31 (0.29, 0.33) |
| Fabiani M [78] 2022 | BNT162b2 or mRNA-1273 | After dose 2 (≥ 14) | Cohort study | ≥80 | Severe COVID-19 | Delta (B.1.617.2) | Italy | 77 (69, 82) | 0.23 (0.18, 0.31) |
| Fabiani M [78] 2022 | BNT162b2 or mRNA-1273 | After dose 2 (≥ 14) | Cohort study | 60-79 | Severe COVID-19 | Delta (B.1.617.2) | Italy | 91 (89, 92) | 0.09 (0.08, 0.11) |
| Fabiani M [78] 2022 | BNT162b2 or mRNA-1273 | After dose 2 (≥ 14) | Cohort study | ≥16 | Severe COVID-19 | Delta (B.1.617.2) | Italy | 91 (90, 92) | 0.09 (0.08, 0.10) |
| Ferdinands JM [85] 2022 | BNT162b2 or mRNA-1273 | After dose 2 (≥ 14) | Cohort study | ≥18 | SARS-CoV-2 infection | Delta (B.1.617.2) | USA | 80 (79, 81) | 0.20 (0.19, 0.21) |
| Ferdinands JM [85] 2022 | BNT162b2 or mRNA-1273 | After dose 2 (≥ 14) | Cohort study | ≥18 | SARS-CoV-2 infection | Omicron (B.1.1.529) | USA | 41 (38, 43) | 0.59 (0.57, 0.62) |
| Ferdinands JM [85] 2022 | BNT162b2 or mRNA-1273 | After dose 2 (≥ 14) | Cohort study | ≥18 | Severe COVID-19 | Omicron (B.1.1.529) | USA | 55 (50, 60) | 0.45 (0.40, 0.50) |
| Ferdinands JM [85] 2022 | BNT162b2 or mRNA-1273 | After dose 2 (≥ 14) | Cohort study | ≥18 | Severe COVID-19 | Delta (B.1.617.2) | USA | 85 (84, 86) | 0.15 (0.14, 0.16) |
| Fowlkes A [79] 2021 | BNT162b2, mRNA-1273, or Ad26.COV2.S | After dose 2 (≥ 14) | Cohort study | > 0 | SARS-CoV-2 infection | Delta (B.1.617.2) | USA | 66 (26, 84) | 0.34 (0.16, 0.74) |
| Hansen CH [64] 2021 | BNT162b2 | After dose 2 (≥ 14) | Cohort study | > 0 | SARS-CoV-2 infection | Delta (B.1.617.2) | Denmark | 74 (72, 75) | 0.26 (0.25, 0.28) |
| Hansen CH [64] 2021 | mRNA-1273 | After dose 2 (≥ 14) | Cohort study | > 0 | SARS-CoV-2 infection | Delta (B.1.617.2) | Denmark | 77 (74, 79) | 0.23 (0.21, 0.26) |
| Hansen CH [64] 2021 | mRNA-1273 | After dose 2 (≥ 14) | Cohort study | > 0 | SARS-CoV-2 infection | Omicron (B.1.1.529) | Denmark | 8 (-51, 38) | 0.92 (0.62, 1.51) |
| Hansen CH [64] 2021 | BNT162b2 | After dose 2 (≥ 14) | Cohort study | > 0 | SARS-CoV-2 infection | Omicron (B.1.1.529) | Denmark | 1 (-26, 20) | 0.99 (0.80, 1.26) |
| Kang M [83] 2022 | HB02, WIV04, CoronaVac, et al. | After dose 2 (≥ 14) | Cohort study | ≥18 | SARS-CoV-2 infection | Delta (B.1.617.2) | China | 52 (20, 83) | 0.48 (0.17, 0.80) |
| Kang M [83] 2022 | HB02, WIV04, CoronaVac, et al. | After dose 2 (≥ 14) | Cohort study | ≥18 | Symptomatic COVID-19 | Delta (B.1.617.2) | China | 60 (32, 89) | 0.40 (0.11, 0.68) |
| Kirsebom FCM [111] 2022 | BNT162b2, mRNA-1273, or ChAdOx1 nCoV-19 | After dose 2 (≥ 14) | Case-control study | ≥18 | Symptomatic COVID-19 | Omicron (B.1.1.529) | UK | 27 (25, 29) | 0.73 (0.71, 0.75) |
| Kirsebom FCM [111] 2022 | BNT162b2, mRNA-1273, or ChAdOx1 nCoV-19 | After dose 2 (≥ 14) | Case-control study | ≥18 | Symptomatic COVID-19 | Omicron (BA.2) | UK | 33 (29, 36) | 0.67 (0.64, 0.71) |
| Kiss Z [108] 2022 | BNT162b2, mRNA-1273, Sputnik V, et al. | After dose 2 (≥ 14) | Cohort study | ≥16 | COVID-19-related death | Delta (B.1.617.2) | Hungary | 73 (72, 75) | 0.27 (0.25, 0.28) |
| Kiss Z [108] 2022 | BNT162b2, mRNA-1273, Sputnik V, et al. | After dose 2 (≥ 14) | Cohort study | 65-74 | COVID-19-related death | Delta (B.1.617.2) | Hungary | 76 (73, 78) | 0.24 (0.22, 0.27) |
| Kiss Z [108] 2022 | BNT162b2, mRNA-1273, Sputnik V, et al. | After dose 2 (≥ 14) | Cohort study | 75-84 | COVID-19-related death | Delta (B.1.617.2) | Hungary | 71 (68, 73) | 0.29 (0.27, 0.32) |
| Kiss Z [108] 2022 | BNT162b2, mRNA-1273, Sputnik V, et al. | After dose 2 (≥ 14) | Cohort study | ≥85 | COVID-19-related death | Delta (B.1.617.2) | Hungary | 57 (51, 62) | 0.43 (0.38, 0.49) |
| Kiss Z [108] 2022 | BNT162b2, mRNA-1273, Sputnik V, et al. | After dose 2 (≥ 14) | Cohort study | ≥16 | COVID-19-related death | Omicron (B.1.1.529) | Hungary | 40 (35, 45) | 0.60 (0.55, 0.65) |
| Kiss Z [108] 2022 | BNT162b2, mRNA-1273, Sputnik V, et al. | After dose 2 (≥ 14) | Cohort study | 65-74 | COVID-19-related death | Omicron (B.1.1.529) | Hungary | 46 (36, 56) | 0.54 (0.44, 0.64) |
| Kiss Z [108] 2022 | BNT162b2, mRNA-1273, Sputnik V, et al. | After dose 2 (≥ 14) | Cohort study | 75-84 | COVID-19-related death | Omicron (B.1.1.529) | Hungary | 42 (32, 51) | 0.58 (0.49, 0.68) |
| Kiss Z [108] 2022 | BNT162b2, mRNA-1273, Sputnik V, et al. | After dose 2 (≥ 14) | Cohort study | ≥85 | COVID-19-related death | Omicron (B.1.1.529) | Hungary | 19 (4, 32) | 0.81 (0.68, 0.96) |
| Klein NP [57] 2022 | BNT162b2 | After dose 2 (≥ 14) | Cohort study | 16-17 | SARS-CoV-2 infection | Delta (B.1.617.2) | USA | 81 (74, 87) | 0.19 (0.13, 0.26) |
| Klein NP [57] 2022 | BNT162b2 | After dose 2 (≥ 14) | Cohort study | 12-15 | SARS-CoV-2 infection | Delta (B.1.617.2) | USA | 86 (79, 90) | 0.14 (0.10, 0.21) |
| Klein NP [57] 2022 | BNT162b2 | After dose 2 (≥ 14) | Cohort study | 16-17 | SARS-CoV-2 infection | Omicron (B.1.1.529) | USA | 16 (-11, 36) | 0.84 (0.64, 1.11) |
| Klein NP [57] 2022 | BNT162b2 | After dose 2 (≥ 14) | Cohort study | 12-15 | SARS-CoV-2 infection | Omicron (B.1.1.529) | USA | 22 (3, 37) | 0.78 (0.63, 0.97) |
| Klein NP [57] 2022 | BNT162b2 | After dose 2 (≥ 14) | Cohort study | 5-11 | SARS-CoV-2 infection | Omicron (B.1.1.529) | USA | 51 (30, 65) | 0.49 (0.35, 0.70) |
| Veneti L [110] 2022 | BNT162b2 | After dose 2 (≥ 7) | Cohort study | 16-17 | SARS-CoV-2 infection | Delta (B.1.617.2) | Norway | 90 (83, 94) | 0.10 (0.06, 0.17) |
| Veneti L [110] 2022 | BNT162b2 | After dose 2 (≥ 7) | Cohort study | 16-17 | SARS-CoV-2 infection | Omicron (B.1.1.529) | Norway | 41 (27, 52) | 0.59 (0.48, 0.73) |
| Lauring AS [71] 2022 | BNT162b2 or mRNA-1273 | After dose 2 (≥ 14) | Case-control study | ≥18 | Severe COVID-19 | Omicron (B.1.1.529) | USA | 65 (51, 75) | 0.35 (0.25, 0.49) |
| Lauring AS [71] 2022 | BNT162b2 or mRNA-1273 | After dose 2 (≥ 14) | Case-control study | ≥65 | Severe COVID-19 | Delta (B.1.617.2) | USA | 81 (77, 84) | 0.19 (0.16, 0.23) |
| Lauring AS [71] 2022 | BNT162b2 | After dose 2 (≥ 14) | Case-control study | ≥18 | Severe COVID-19 | Delta (B.1.617.2) | USA | 82 (80, 84) | 0.18 (0.16, 0.20) |
| Lauring AS [71] 2022 | mRNA-1273 | After dose 2 (≥ 14) | Case-control study | ≥18 | Severe COVID-19 | Delta (B.1.617.2) | USA | 88 (86, 90) | 0.12 (0.10, 0.14) |
| Li XN [61] 2021 | HB02, WIV04, CoronaVac, et al. | After dose 2 (≥ 14) | Case-control study | 18-59 | Symptomatic COVID-19 | Delta (B.1.617.2) | China | 59 (16, 81.6) | 0.41 (0.19–0.84) |
| Lopez Bernal J [65] 2021 | ChAdOx1 nCoV-19 | After dose 2 (≥ 14) | Case-control study | ≥16 | Symptomatic COVID-19 | Delta (B.1.617.2) | UK | 67 (61, 72) | 0.33 (0.28, 0.39) |
| Lopez Bernal J [65] 2021 | BNT162b2 | After dose 2 (≥ 14) | Case-control study | ≥16 | Symptomatic COVID-19 | Delta (B.1.617.2) | UK | 88 (85, 90) | 0.12 (0.10, 0.15) |
| Martínez-Baz I [101] 2021 | Ad26.COV2.S | After single dose (≥ 14) | Cohort study | ≥18 | SARS-CoV-2 infection | Delta (B.1.617.2) | Spain | 42 (18, 59) | 0.58 (0.41, 0.82) |
| Martínez-Baz I [101] 2021 | ChAdOx1 nCoV-19 | After dose 2 (≥ 14) | Cohort study | ≥18 | SARS-CoV-2 infection | Delta (B.1.617.2) | Spain | 55 (39, 67) | 0.45 (0.33, 0.61) |
| Martínez-Baz I [101] 2021 | BNT162b2 | After dose 2 (≥ 14) | Cohort study | ≥18 | SARS-CoV-2 infection | Delta (B.1.617.2) | Spain | 67 (59, 74) | 0.33 (0.26, 0.41) |
| Martínez-Baz I [101] 2021 | mRNA-1273 | After dose 2 (≥ 14) | Cohort study | ≥18 | SARS-CoV-2 infection | Delta (B.1.617.2) | Spain | 77 (64, 85) | 0.23 (0.15, 0.36) |
| McMenamin ME [55] 2022 | CoronaVac | After dose 2 (≥ 14) | Cohort study | 20-59 | Severe COVID-19 | Omicron (BA.2) | China | 92 (88, 94) | 0.08 (0.06, 0.12) |
| McMenamin ME [55] 2022 | CoronaVac | After dose 2 (≥ 14) | Cohort study | 60-69 | Severe COVID-19 | Omicron (BA.2) | China | 83 (74, 88) | 0.17 (0.12, 0.26) |
| McMenamin ME [55] 2022 | CoronaVac | After dose 2 (≥ 14) | Cohort study | 70-79 | Severe COVID-19 | Omicron (BA.2) | China | 81 (73, 87) | 0.19 (0.13, 0.27) |
| McMenamin ME [55] 2022 | CoronaVac | After dose 2 (≥ 14) | Cohort study | ≥ 80 | Severe COVID-19 | Omicron (BA.2) | China | 60 (44, 72) | 0.40 (0.28, 0.56) |
| McMenamin ME [55] 2022 | BNT162b2 | After dose 2 (≥ 14) | Cohort study | 20-59 | Severe COVID-19 | Omicron (BA.2) | China | 95 (93, 97) | 0.05 (0.03, 0.07) |
| McMenamin ME [55] 2022 | BNT162b2 | After dose 2 (≥ 14) | Cohort study | 60-69 | Severe COVID-19 | Omicron (BA.2) | China | 91 (85, 95) | 0.09 (0.05, 0.15) |
| McMenamin ME [55] 2022 | BNT162b2 | After dose 2 (≥ 14) | Cohort study | 70-79 | Severe COVID-19 | Omicron (BA.2) | China | 89 (83, 93) | 0.11 (0.07, 0.17) |
| McMenamin ME [55] 2022 | BNT162b2 | After dose 2 (≥ 14) | Cohort study | ≥ 80 | Severe COVID-19 | Omicron (BA.2) | China | 85 (76, 90) | 0.15 (0.10, 0.24) |
| McMenamin ME [55] 2022 | CoronaVac | After dose 2 (≥ 14) | Cohort study | 20-59 | COVID-19-related death | Omicron (BA.2) | China | 94 (90, 97) | 0.06 (0.03, 0.10) |
| McMenamin ME [55] 2022 | CoronaVac | After dose 2 (≥ 14) | Cohort study | 60-69 | COVID-19-related death | Omicron (BA.2) | China | 88 (81, 92) | 0.12 (0.08, 0.19) |
| McMenamin ME [55] 2022 | CoronaVac | After dose 2 (≥ 14) | Cohort study | 70-79 | COVID-19-related death | Omicron (BA.2) | China | 84 (78, 89) | 0.16 (0.11, 0.22) |
| McMenamin ME [55] 2022 | CoronaVac | After dose 2 (≥ 14) | Cohort study | ≥ 80 | COVID-19-related death | Omicron (BA.2) | China | 67 (52, 77) | 0.33 (0.23, 0.48) |
| McMenamin ME [55] 2022 | BNT162b2 | After dose 2 (≥ 14) | Cohort study | 20-59 | COVID-19-related death | Omicron (BA.2) | China | 96 (94, 98) | 0.04 (0.02, 0.06) |
| McMenamin ME [55] 2022 | BNT162b2 | After dose 2 (≥ 14) | Cohort study | 60-69 | COVID-19-related death | Omicron (BA.2) | China | 94 (89, 97) | 0.06 (0.03, 0.11) |
| McMenamin ME [55] 2022 | BNT162b2 | After dose 2 (≥ 14) | Cohort study | 70-79 | COVID-19-related death | Omicron (BA.2) | China | 92 (87, 96) | 0.08 (0.04, 0.13) |
| McMenamin ME [55] 2022 | BNT162b2 | After dose 2 (≥ 14) | Cohort study | ≥ 80 | COVID-19-related death | Omicron (BA.2) | China | 88 (80, 93) | 0.12 (0.07, 0.20) |
| McMenamin ME [55] 2022 | CoronaVac | After dose 2 (≥ 14) | Cohort study | 20-59 | Symptomatic COVID-19 | Omicron (BA.2) | China | 18 (-18, 43) | 0.82 (0.57, 1.18) |
| McMenamin ME [55] 2022 | BNT162b2 | After dose 2 (≥ 14) | Cohort study | 20-59 | Symptomatic COVID-19 | Omicron (BA.2) | China | 31 (2, 52) | 0.69 (0.48, 0.98) |
| Nanduri S [87] 2021 | mRNA-1273 | After dose 2 (≥ 14) | Cohort study | > 0 | SARS-CoV-2 infection | Delta (B.1.617.2) | USA | 51 (45, 56) | 0.49 (0.44, 0.55) |
| Nanduri S [87] 2021 | BNT162b2 | After dose 2 (≥ 14) | Cohort study | > 0 | SARS-CoV-2 infection | Delta (B.1.617.2) | USA | 52 (48, 56) | 0.48 (0.44, 0.52) |
| Nasreen S [74] 2021 | ChAdOx1 nCoV-19 | After dose 2 (≥ 14) | Case-control study | ≥16 | Severe COVID-19 | Delta (B.1.617.2) | Canada | 90 (67, 97) | 0.10 (0.03, 0.33) |
| Nasreen S [74] 2021 | BNT162b2 | After dose 2 (≥ 7) | Case-control study | ≥16 | Severe COVID-19 | Delta (B.1.617.2) | Canada | 97 (96, 98) | 0.03 (0.02, 0.04) |
| Nasreen S [74] 2021 | mRNA-1273 | After dose 2 (≥ 14) | Case-control study | ≥16 | Severe COVID-19 | Delta (B.1.617.2) | Canada | 98 (93, 99) | 0.02 (0.01, 0.07) |
| Nasreen S [74] 2021 | BNT162b2 | After dose 2 (≥ 14) | Case-control study | ≥16 | Severe COVID-19 | Delta (B.1.617.2) | Canada | 98 (96, 99) | 0.02 (0.01, 0.04) |
| Nasreen S [74] 2021 | ChAdOx1 nCoV-19 | After dose 2 (≥ 14) | Case-control study | ≥16 | Symptomatic COVID-19 | Delta (B.1.617.2) | Canada | 88 (68, 96) | 0.12 (0.04, 0.32) |
| Nasreen S [74] 2021 | BNT162b2 | After dose 2 (≥ 7) | Case-control study | ≥16 | Symptomatic COVID-19 | Delta (B.1.617.2) | Canada | 89 (83, 93) | 0.11 (0.07, 0.17) |
| Nasreen S [74] 2021 | BNT162b2 | After dose 2 (≥ 14) | Case-control study | ≥16 | Symptomatic COVID-19 | Delta (B.1.617.2) | Canada | 92 (89, 94) | 0.08 (0.06, 0.11) |
| Nasreen S [74] 2021 | BNT162b2 | After dose 2 (≥ 7) | Case-control study | ≥16 | Symptomatic COVID-19 | Delta (B.1.617.2) | Canada | 92 (90, 94) | 0.08 (0.06, 0.10) |
| Nasreen S [74] 2021 | mRNA-1273 | After dose 2 (≥ 14) | Case-control study | ≥16 | Symptomatic COVID-19 | Delta (B.1.617.2) | Canada | 94 (90, 97) | 0.06 (0.03, 0.10) |
| Nunes MC [77] 2022 | BNT162b2 | After dose 2 (≥ 14) | Case-control study | > 0 | SARS-CoV-2 infection | Omicron (B.1.1.529) | South Africa | 41 (-57, 77) | 0.59 (0.23, 1.57) |
| Nunes MC [77] 2022 | Ad26.COV2.S | After full vaccination (≥ 14) | Case-control study | > 0 | SARS-CoV-2 infection | Omicron (B.1.1.529) | South Africa | 19 (-43, 54) | 0.81 (0.46, 1.43) |
| Poukka E [102] 2021 | BNT162b2 or mRNA-1273 | After dose 2 (≥ 14) | Cohort study | 16-70 | SARS-CoV-2 infection | Delta (B.1.617.2) | Finland | 69 (62, 75) | 0.31 (0.25, 0.38) |
| Poukka E [102] 2021 | Ad26.COV2.S, BNT162b2 or mRNA-1273 | After full vaccination (≥ 14) | Cohort study | 16-70 | SARS-CoV-2 infection | Delta (B.1.617.2) | Finland | 72 (53, 73) | 0.28 (0.27, 0.47) |
| Poukka E [102] 2021 | Ad26.COV2.S | After fully vaccined (≥ 14) | Cohort study | 16-70 | SARS-CoV-2 infection | Delta (B.1.617.2) | Finland | 75 (-53, 95) | 0.25 (0.05, 1.53) |
| Poukka E [102] 2021 | BNT162b2 or mRNA-1273 | After dose 2 (≥ 14) | Cohort study | 16-70 | Severe COVID-19 | Delta (B.1.617.2) | Finland | 98 (92, 99) | 0.02 (0.01, 0.08) |
| Pouwels KB [86] 2021 | ChAdOx1 nCoV-19 | After dose 2 (≥ 14) | Case-control study | ≥18 | SARS-CoV-2 infection | Delta (B.1.617.2) | UK | 67 (62, 71) | 0.33 (0.29, 0.38) |
| Pouwels KB [86] 2021 | ChAdOx1 nCoV-19 | After dose 2 (≥ 14) | Case-control study | 18-64 | SARS-CoV-2 infection | Delta (B.1.617.2) | UK | 67 (62, 71) | 0.33 (0.29, 0.38) |
| Pouwels KB [86] 2021 | BNT162b2 | After dose 2 (≥ 14) | Case-control study | ≥18 | SARS-CoV-2 infection | Delta (B.1.617.2) | UK | 80 (77, 83) | 0.20 (0.17, 0.23) |
| Pouwels KB [86] 2021 | BNT162b2 | After dose 2 (≥ 14) | Case-control study | 18-64 | SARS-CoV-2 infection | Delta (B.1.617.2) | UK | 82 (79–85) | 0.18 (0.15, 0.21) |
| Pouwels KB [86] 2021 | ChAdOx1 nCoV-19 | After dose 2 (≥ 14) | Case-control study | 18-64 | Symptomatic COVID-19 | Delta (B.1.617.2) | UK | 70 (66–74) | 0.30 (0.26, 0.34) |
| Pouwels KB [86] 2021 | ChAdOx1 nCoV-19 | After dose 2 (≥ 14) | Case-control study | ≥18 | Symptomatic COVID-19 | Delta (B.1.617.2) | UK | 71 (66, 74) | 0.29 (0.26, 0.34) |
| Pouwels KB [86] 2021 | BNT162b2 | After dose 2 (≥ 14) | Case-control study | ≥18 | Symptomatic COVID-19 | Delta (B.1.617.2) | UK | 84 (82, 86) | 0.16 (0.14, 0.18) |
| Pouwels KB [86] 2021 | BNT162b2 | After dose 2 (≥ 14) | Case-control study | 18-64 | Symptomatic COVID-19 | Delta (B.1.617.2) | UK | 86 (83–88) | 0.14 (0.12, 0.17) |
| Powell AA [62] 2021 | BNT162b2 | After dose 2 (≥ 14) | Case-control study | 12-15 | Symptomatic COVID-19 | Omicron (B.1.1.529) | UK | 73 (66, 78) | 0.27 (0.22, 0.34) |
| Powell AA [62] 2021 | BNT162b2 | After dose 2 (≥ 7) | Case-control study | 16-17 | Symptomatic COVID-19 | Omicron (B.1.1.529) | UK | 76 (73, 79) | 0.24 (0.21, 0.27) |
| Powell AA [62] 2021 | BNT162b2 | After dose 2 (≥ 7) | Case-control study | 12-15 | Symptomatic COVID-19 | Omicron (B.1.1.529) | UK | 78 (72, 83) | 0.22 (0.17, 0.28) |
| Powell AA [62] 2021 | BNT162b2 | After dose 2 (≥ 14) | Case-control study | 12-15 | Symptomatic COVID-19 | Delta (B.1.617.2) | UK | 87 (74, 94) | 0.13 (0.06, 0.26) |
| Powell AA [62] 2021 | BNT162b2 | After dose 2 (≥ 7) | Case-control study | 12-15 | Symptomatic COVID-19 | Delta (B.1.617.2) | UK | 90 (78, 96) | 0.10 (0.04, 0.22) |
| Powell AA [62] 2021 | BNT162b2 | After dose 2 (≥ 7) | Case-control study | 16-17 | Symptomatic COVID-19 | Delta (B.1.617.2) | UK | 93 (92, 94) | 0.07 (0.06, 0.08) |
| Price AM [107] 2022 | BNT162b2 | After dose 2 (≥ 14) | Case-control study | 12-18 | Severe COVID-19 | Omicron (B.1.1.529) | USA | 40 (9, 60) | 0.60 (0.40, 0.91) |
| Price AM [107] 2022 | BNT162b2 | After dose 2 (≥ 14) | Case-control study | 5-11 | Severe COVID-19 | Omicron (B.1.1.529) | USA | 68 (42, 82) | 0.32 (0.18, 0.58) |
| Price AM [107] 2022 | BNT162b2 | After dose 2 (≥ 14) | Case-control study | 12-18 | Severe COVID-19 | Delta (B.1.617.2) | USA | 92 (89, 95) | 0.08 (0.05, 0.11) |
| Reis BY [104] 2021 | BNT162b2 | After dose 2 (≥ 7) | Cohort study | 12-18 | SARS-CoV-2 infection | Delta (B.1.617.2) | Israel | 90 (88, 92) | 0.10 (0.08, 0.12) |
| Reis BY [104] 2021 | BNT162b2 | After dose 2 (≥ 7) | Cohort study | 12-18 | Symptomatic COVID-19 | Delta (B.1.617.2) | Israel | 93 (88, 97) | 0.07 (0.03, 0.12) |
| Sadoff J [53] 2022 | Ad26.COV2.S | After single dose (≥ 14) | RCT | ≥18 | Symptomatic COVID-19 | Delta (B.1.617.2) | Multiple | −6 (−178, 59) | 1.06 (0.41, 2.78) |
| Seppälä E [84] 2021 | BNT162b2, mRNA-1273, or ChAdOx1 nCoV-19 | After dose 2 (≥ 14) | Cohort study | ≥18 | SARS-CoV-2 infection | Delta (B.1.617.2) | Norway | 65 (61, 68) | 0.35 (0.32, 0.39) |
| Sheikh A [82] 2021 | ChAdOx1 nCoV-19 | After dose 2 (≥ 14) | Case-control study | > 0 | SARS-CoV-2 infection | Delta (B.1.617.2) | UK | 60 (53, 66) | 0.40 (0.34, 0.47) |
| Sheikh A [82] 2021 | BNT162b2 | After dose 2 (≥ 14) | Case-control study | > 0 | SARS-CoV-2 infection | Delta (B.1.617.2) | UK | 79 (75, 82) | 0.21 (0.18, 0.25) |
| Sheikh A [82] 2021 | ChAdOx1 nCoV-19 | After dose 2 (≥ 14) | Case-control study | > 0 | Symptomatic COVID-19 | Delta (B.1.617.2) | UK | 61 (51, 70) | 0.39 (0.30, 0.49) |
| Sheikh A [82] 2021 | BNT162b2 | After dose 2 (≥ 14) | Case-control study | > 0 | Symptomatic COVID-19 | Delta (B.1.617.2) | UK | 83 (78, 87) | 0.17 (0.13, 0.22) |
| Skowronski DM [103] 2021 | ChAdOx1 nCoV-19 | After dose 2 (≥ 14) | Case-control study | ≥18 | SARS-CoV-2 infection | Delta (B.1.617.2) | Canada | 70 (66, 73) | 0.30 (0.27, 0.34) |
| Skowronski DM [103] 2021 | ChAdOx1 nCoV-19 | After dose 2 (≥ 14) | Case-control study | ≥18 | SARS-CoV-2 infection | Delta (B.1.617.2) | Canada | 73 (69, 78) | 0.27 (0.22, 0.31) |
| Skowronski DM [103] 2021 | BNT162b2, mRNA-1273, or ChAdOx1 nCoV-19 | After dose 2 (≥ 14) | Case-control study | ≥18 | SARS-CoV-2 infection | Delta (B.1.617.2) | Canada | 88 (85, 89) | 0.12 (0.11, 0.15) |
| Skowronski DM [103] 2021 | BNT162b2 or mRNA-1273 | After dose 2 (≥ 14) | Case-control study | ≥18 | SARS-CoV-2 infection | Delta (B.1.617.2) | Canada | 89 (86, 91) | 0.11 (0.09, 0.14) |
| Skowronski DM [103] 2021 | BNT162b2 | After dose 2 (≥ 14) | Case-control study | ≥18 | SARS-CoV-2 infection | Delta (B.1.617.2) | Canada | 89 (88, 90) | 0.11 (0.10, 0.12) |
| Skowronski DM [103] 2021 | BNT162b2, mRNA-1273, or ChAdOx1 nCoV-19 | After dose 2 (≥ 14) | Case-control study | ≥18 | SARS-CoV-2 infection | Delta (B.1.617.2) | Canada | 91 (89, 92) | 0.09 (0.08, 0.12) |
| Skowronski DM [103] 2021 | mRNA-1273 | After dose 2 (≥ 14) | Case-control study | ≥18 | SARS-CoV-2 infection | Delta (B.1.617.2) | Canada | 91 (90, 92) | 0.09 (0.08, 0.10) |
| Skowronski DM [103] 2021 | BNT162b2 | After dose 2 (≥ 14) | Case-control study | ≥18 | SARS-CoV-2 infection | Delta (B.1.617.2) | Canada | 91 (90, 92) | 0.09 (0.08, 0.10) |
| Skowronski DM [103] 2021 | mRNA-1273 | After dose 2 (≥ 14) | Case-control study | ≥18 | SARS-CoV-2 infection | Delta (B.1.617.2) | Canada | 92 (91, 93) | 0.08 (0.07, 0.09) |
| Skowronski DM [103] 2021 | BNT162b2 or mRNA-1273 | After dose 2 (≥ 14) | Case-control study | ≥18 | SARS-CoV-2 infection | Delta (B.1.617.2) | Canada | 92 (91, 93) | 0.08 (0.07, 0.09) |
| Skowronski DM [103] 2021 | ChAdOx1 nCoV-19 | After dose 2 (≥ 14) | Case-control study | ≥18 | Severe COVID-19 | Delta (B.1.617.2) | Canada | 92 (86, 95) | 0.08 (0.05, 0.14) |
| Skowronski DM [103] 2021 | ChAdOx1 nCoV-19 | After dose 2 (≥ 14) | Case-control study | ≥18 | Severe COVID-19 | Delta (B.1.617.2) | Canada | 94 (89, 97) | 0.06 (0.03, 0.11) |
| Skowronski DM [103] 2021 | BNT162b2 or mRNA-1273 | After dose 2 (≥ 14) | Case-control study | ≥18 | Severe COVID-19 | Delta (B.1.617.2) | Canada | 95 (86, 98) | 0.05 (0.02, 0.14) |
| Skowronski DM [103] 2021 | mRNA-1273 | After dose 2 (≥ 14) | Case-control study | ≥18 | Severe COVID-19 | Delta (B.1.617.2) | Canada | 97 (96, 98) | 0.03 (0.02, 0.04) |
| Skowronski DM [103] 2021 | BNT162b2 | After dose 2 (≥ 14) | Case-control study | ≥18 | Severe COVID-19 | Delta (B.1.617.2) | Canada | 97 (96, 98) | 0.03 (0.02, 0.04) |
| Skowronski DM [103] 2021 | BNT162b2, mRNA-1273, or ChAdOx1 nCoV-19 | After dose 2 (≥ 14) | Case-control study | ≥18 | Severe COVID-19 | Delta (B.1.617.2) | Canada | 98 (94, 99) | 0.02 (0.01, 0.06) |
| Skowronski DM [103] 2021 | mRNA-1273 | After dose 2 (≥ 14) | Case-control study | ≥18 | Severe COVID-19 | Delta (B.1.617.2) | Canada | 98 (96, 99) | 0.02 (0.01, 0.04) |
| Skowronski DM [103] 2021 | BNT162b2 | After dose 2 (≥ 14) | Case-control study | ≥18 | Severe COVID-19 | Delta (B.1.617.2) | Canada | 98 (97, 99) | 0.02 (0.01, 0.03) |
| Skowronski DM [103] 2021 | BNT162b2 or mRNA-1273 | After dose 2 (≥ 14) | Case-control study | ≥18 | Severe COVID-19 | Delta (B.1.617.2) | Canada | 98 (97, 99) | 0.02 (0.01, 0.03) |
| Skowronski DM [103] 2021 | BNT162b2, mRNA-1273, or ChAdOx1 nCoV-19 | After dose 2 (≥ 14) | Case-control study | ≥18 | Severe COVID-19 | Delta (B.1.617.2) | Canada | 98 (97, 99) | 0.02 (0.01, 0.03) |
| Šmíd M [63] 2022 | BNT162b2, mRNA-1273, Ad26.COV2.S et al. | After full vaccination (≥ 14) | Cohort study | > 0 | SARS-CoV-2 infection | Delta (B.1.617.2) | Czech | 73 (72, 74) | 0.27 (0.26, 0.28) |
| Šmíd M [63] 2022 | BNT162b2, mRNA-1273, Ad26.COV2.S et al. | After full vaccination (≥ 14) | Cohort study | > 0 | SARS-CoV-2 infection | Omicron (B.1.1.529) | Czech | 43 (42, 44) | 0.57 (0.56, 0.58) |
| Šmíd M [63] 2022 | BNT162b2, mRNA-1273, Ad26.COV2.S et al. | After full vaccination (≥ 14) | Cohort study | > 0 | Severe COVID-19 | Omicron (B.1.1.529) | Czech | 45 (29, 57) | 0.55 (0.43, 0.71) |
| Sritipsukho P [81] 2022 | CoronaVac | After dose 2 (≥ 14) | Case-control study | ≥18 | SARS-CoV-2 infection | Delta (B.1.617.2) | Thailand | 60 (49, 69) | 0.40 (0.31, 0.51) |
| Sritipsukho P [81] 2022 | CoronaVac or ChAdOx1 nCoV-19 | After dose 2 (≥ 14) | Case-control study | ≥18 | SARS-CoV-2 infection | Delta (B.1.617.2) | Thailand | 74 (43, 88) | 0.26 (0.12, 0.57) |
| Sritipsukho P [81] 2022 | ChAdOx1 nCoV-19 | After dose 2 (≥ 14) | Case-control study | ≥18 | SARS-CoV-2 infection | Delta (B.1.617.2) | Thailand | 83 (70, 90) | 0.17 (0.10, 0.30) |
| Starrfelt J [109] 2022 | BNT162b2, mRNA-1273, Ad26.COV2.S et al. | After full vaccination (≥ 14) | Cohort study | ≥18 | SARS-CoV-2 infection | Delta (B.1.617.2) | Norway | 46 (44, 48) | 0.54 (0.52, 0.56) |
| Starrfelt J [109] 2022 | BNT162b2, mRNA-1273, Ad26.COV2.S et al. | After full vaccination (≥ 14) | Cohort study | ≥18 | SARS-CoV-2 infection | Delta (B.1.617.2) | Norway | 64 (63, 66) | 0.36 (0.34, 0.37) |
| Starrfelt J [109] 2022 | BNT162b2, mRNA-1273, Ad26.COV2.S et al. | After full vaccination (≥ 14) | Cohort study | ≥18 | Severe COVID-19 | Delta (B.1.617.2) | Norway | 91 (86, 94) | 0.09 (0.06, 0.14) |
| Starrfelt J [109] 2022 | BNT162b2, mRNA-1273, Ad26.COV2.S et al. | After full vaccination (≥ 14) | Cohort study | ≥18 | Severe COVID-19 | Delta (B.1.617.2) | Norway | 92 (90, 94) | 0.08 (0.06, 0.10) |
| Starrfelt J [109] 2022 | BNT162b2, mRNA-1273, Ad26.COV2.S et al. | After full vaccination (≥ 14) | Cohort study | ≥18 | COVID-19-related death | Delta (B.1.617.2) | Norway | 90 (89, 93) | 0.10 (0.07, 0.11) |
| Starrfelt J [109] 2022 | BNT162b2, mRNA-1273, Ad26.COV2.S et al. | After full vaccination (≥ 14) | Cohort study | ≥18 | COVID-19-related death | Delta (B.1.617.2) | Norway | 83 (62, 92) | 0.17 (0.08, 0.38) |
| Tang P [60] 2021 | BNT162b2 or mRNA-1273 | After dose 2 (≥ 14) | Case-control study | > 0 | Asymptomatic infection | Delta (B.1.617.2) | Qatar | 46 (33, 56) | 0.54 (0.44, 0.67) |
| Tang P [60] 2021 | BNT162b2 | After dose 2 (≥ 14) | Case-control study | > 0 | Asymptomatic infection | Delta (B.1.617.2) | Qatar | 46 (32, 57) | 0.54 (0.43, 0.68) |
| Tang P [60] 2021 | mRNA-1273 | After dose 2 (≥ 14) | Case-control study | > 0 | Asymptomatic infection | Delta (B.1.617.2) | Qatar | 54 (33, 68) | 0.46 (0.32, 0.67) |
| Tang P [60] 2021 | BNT162b2 | After dose 2 (≥ 14) | Case-control study | ≥50 | SARS-CoV-2 infection | Delta (B.1.617.2) | Qatar | 17 (-21, 43) | 0.83 (0.57, 1.21) |
| Tang P [60] 2021 | BNT162b2 or mRNA-1273 | After dose 2 (≥ 14) | Case-control study | ≥50 | SARS-CoV-2 infection | Delta (B.1.617.2) | Qatar | 30 (-0.1, 50) | 0.70 (0.5, 1.01) |
| Tang P [60] 2021 | BNT162b2 | After dose 2 (≥ 14) | Case-control study | > 0 | SARS-CoV-2 infection | Delta (B.1.617.2) | Qatar | 51 (45, 55) | 0.49 (0.45, 0.55) |
| Tang P [60] 2021 | BNT162b2 or mRNA-1273 | After dose 2 (≥ 14) | Case-control study | > 0 | SARS-CoV-2 infection | Delta (B.1.617.2) | Qatar | 54 (50, 58) | 0.46 (0.42, 0.50) |
| Tang P [60] 2021 | mRNA-1273 | After dose 2 (≥ 14) | Case-control study | ≥50 | SARS-CoV-2 infection | Delta (B.1.617.2) | Qatar | 65 (16, 85) | 0.35 (0.15, 0.84) |
| Tang P [60] 2021 | mRNA-1273 | After dose 2 (≥ 14) | Case-control study | > 0 | SARS-CoV-2 infection | Delta (B.1.617.2) | Qatar | 72 (66, 77) | 0.28 (0.23, 0.34) |
| Tang P [60] 2021 | BNT162b2 | After dose 2 (≥ 14) | Case-control study | ≥50 | Severe COVID-19 | Delta (B.1.617.2) | Qatar | 82 (50, 93) | 0.18 (0.07, 0.50) |
| Tang P [60] 2021 | BNT162b2 or mRNA-1273 | After dose 2 (≥ 14) | Case-control study | ≥50 | Severe COVID-19 | Delta (B.1.617.2) | Qatar | 85 (53, 95) | 0.15 (0.05, 0.47) |
| Tang P [60] 2021 | BNT162b2 or mRNA-1273 | After dose 2 (≥ 14) | Case-control study | > 0 | Severe COVID-19 | Delta (B.1.617.2) | Qatar | 93 (85, 97) | 0.07 (0.03, 0.15) |
| Tang P [60] 2021 | BNT162b2 | After dose 2 (≥ 14) | Case-control study | > 0 | Severe COVID-19 | Delta (B.1.617.2) | Qatar | 94 (86, 98) | 0.06 (0.02, 0.14) |
| Tang P [60] 2021 | mRNA-1273 | After dose 2 (≥ 14) | Case-control study | > 0 | Severe COVID-19 | Delta (B.1.617.2) | Qatar | 96 (71, 99) | 0.04 (0.01, 0.29) |
| Tang P [60] 2021 | BNT162b2 | After dose 2 (≥ 14) | Case-control study | > 0 | Symptomatic COVID-19 | Delta (B.1.617.2) | Qatar | 44 (37, 51) | 0.56 (0.49, 0.63) |
| Tang P [60] 2021 | BNT162b2 or mRNA-1273 | After dose 2 (≥ 14) | Case-control study | > 0 | Symptomatic COVID-19 | Delta (B.1.617.2) | Qatar | 49 (43, 55) | 0.51 (0.45, 0.57) |
| Tang P [60] 2021 | mRNA-1273 | After dose 2 (≥ 14) | Case-control study | > 0 | Symptomatic COVID-19 | Delta (B.1.617.2) | Qatar | 74 (66, 80) | 0.26 (0.20, 0.34) |
| Tartof SY [80] 2021 | BNT162b2 | After dose 2 (≥ 7) | Cohort study | ≥12 | SARS-CoV-2 infection | Delta (B.1.617.2) | USA | 75 (71, 78) | 0.25 (0.22, 0.29) |
| Tartof SY [80] 2021 | BNT162b2 | After dose 2 (≥ 7) | Cohort study | ≥12 | Severe COVID-19 | Delta (B.1.617.2) | USA | 93 (84, 96) | 0.07 (0.04, 0.16) |
| Thiruvengadam R [58] 2022 | ChAdOx1 nCoV-19 | After dose 2 (≥ 14) | Case-control study | > 0 | SARS-CoV-2 infection | Delta (B.1.617.2) | India | 63 (52, 72) | 0.37 (0.28, 0.48) |
| Thiruvengadam R [58] 2022 | ChAdOx1 nCoV-19 | After dose 2 (≥ 14) | Case-control study | > 0 | Severe COVID-19 | Delta (B.1.617.2) | India | 82 (10, 99) | 0.18 (0.01, 0.9) |
| Tseng HF [88] 2022 | mRNA-1273 | After dose 2 (≥ 14) | Case-control study | ≥18 | SARS-CoV-2 infection | Delta (B.1.617.2) | USA | 64 (60, 67) | 0.36 (0.33, 0.40) |
| Tseng HF [88] 2022 | mRNA-1273 | After dose 2 (≥ 14) | Case-control study | ≥18 | SARS-CoV-2 infection | Omicron (B.1.1.529) | USA | 14 (11, 17) | 0.86 (0.83, 0.89) |
| Tseng HF [88] 2022 | mRNA-1273 | After dose 2 (≥ 14) | Case-control study | ≥18 | Severe COVID-19 | Omicron (B.1.1.529) | USA | 85 (23, 97) | 0.15 (0.03, 0.77) |
| Tseng HF [88] 2022 | mRNA-1273 | After dose 2 (≥ 14) | Case-control study | ≥18 | Severe COVID-19 | Delta (B.1.617.2) | USA | 98 (93, 99) | 0.02 (0.01, 0.07) |
| Yinong [73] 2022 | BNT162b2 or mRNA-1273 | After dose 2 (≥ 14) | Case-control study | ≥18 | SARS-CoV-2 infection | Delta (B.1.617.2) | USA | 55 (51, 58) | 0.45 (0.42, 0.49) |
| Yinong [73] 2022 | BNT162b2 or mRNA-1273 | After dose 2 (≥ 14) | Case-control study | ≥18 | SARS-CoV-2 infection | Omicron (B.1.1.529) | USA | 7 (3, 10) | 0.93 (0.90, 0.97) |
| Yinong [73] 2022 | BNT162b2 or mRNA-1273 | After dose 2 (≥ 14) | Case-control study | ≥18 | Severe COVID-19 | Delta (B.1.617.2) | USA | 44 (26, 58) | 0.56 (0.42, 0.74) |
| Yinong [73] 2022 | BNT162b2 or mRNA-1273 | After dose 2 (≥ 14) | Case-control study | ≥18 | Severe COVID-19 | Omicron (B.1.1.529) | USA | 75 (70, 80) | 0.25 (0.20, 0.30) |
| Yinong [73] 2022 | BNT162b2 or mRNA-1273 | After dose 2 (≥ 14) | Case-control study | ≥18 | COVID-19-related death | Delta (B.1.617.2) | USA | 93 (85, 97) | 0.07 (0.03, 0.15) |
| Yinong [73] 2022 | BNT162b2 or mRNA-1273 | After dose 2 (≥ 14) | Case-control study | ≥18 | COVID-19-related death | Omicron (B.1.1.529) | USA | 75 (52, 87) | 0.25 (0.13, 0.48) |
| Yoon SK [112] 2022 | BNT162b2 or mRNA-1273 | After dose 2 (≥ 14) | Cohort study | > 0 | SARS-CoV-2 infection | Delta (B.1.617.2) | USA | 65 (49–76) | 0.35 (0.24, 0.51) |
| Yoon SK [112] 2022 | BNT162b2 or mRNA-1273 | After dose 2 (≥ 14) | Cohort study | > 0 | SARS-CoV-2 infection | Omicron (B.1.1.529) | USA | 46 (25–61) | 0.54 (0.39, 0.75) |

VE: vaccine effectiveness/efficacy; Full vaccination as ≥7 or 14 days after the second dose vaccination for COVID-19 vaccines BNT162b2; ≥14 days after the second dose vaccination for all other COVID-19 vaccines or after the single dose vaccination for COVID-19 vaccines Ad26.COV2.S
