# Supplementary material for: A comprehensive analysis of the efficacy and effectiveness of COVID-19 vaccines
Source: Front Immunol. 2022 Aug 26;13:945930. doi: 10.3389/fimmu.2022.945930 (PMC9459021; doi:10.3389/fimmu.2022.945930)
Supplement: Supplementary file 5 [file Table_4.docx]

**Supplementary Table 4** Effectiveness of included studies for the COVID-19 vaccine booster immunization during the delta and Omicron variants period

| **First author** | **Vaccine booster name** | **Dose and time interval since final dose (days)** | **Age (year)** | **Type of study** | **During variant Period/Variant** | **Type of cases** | **Country** | **Adjusted VE % (95%CI)** | **Adjusted RR /OR (95% CI)** |
| --- | --- | --- | --- | --- | --- | --- | --- | --- | --- |
| **Comparison between booster vaccinees and unvaccinated group** | | | | | | | | | |
| Accorsi EK [89] 2022 | BNT16b2 or mRNA-1273 | After booster dose (≥ 14) | ≥18 | Case-control study | Delta (B.1.617.2) | Symptomatic COVID-19 | USA | 93 (92, 94) | 0.07 (0.06, 0.08) |
| Accorsi EK [89] 2022 | BNT16b2 or mRNA-1273 | After booster dose (≥ 14) | ≥18 | Case-control study | Omicron (B.1.1.529) | Symptomatic COVID-19 | USA | 67 (65, 69) | 0.33 (0.31, 0.35) |
| Accorsi EK [89] 2022 | BNT16b2 | After booster dose (≥ 14) | ≥18 | Case-control study | Delta (B.1.617.2) | Symptomatic COVID-19 | USA | 92 (91, 93) | 0.08 (0.07, 0.09) |
| Accorsi EK [89] 2022 | BNT16b2 | After booster dose (≥ 14) | ≥18 | Case-control study | Omicron (B.1.1.529) | Symptomatic COVID-19 | USA | 65 (62, 68) | 0.35 (0.32, 0.38) |
| Accorsi EK [89] 2022 | mRNA-1273 | After booster dose (≥ 14) | ≥18 | Case-control study | Delta (B.1.617.2) | Symptomatic COVID-19 | USA | 95 (94, 96) | 0.05 (0.04, 0.06) |
| Accorsi EK [89] 2022 | mRNA-1273 | After booster dose (≥ 14) | ≥18 | Case-control study | Omicron (B.1.1.529) | Symptomatic COVID-19 | USA | 72 (69, 74) | 0.28 (0.26, 0.31) |
| Andrews N [97] 2022 | BNT162b2 | After booster dose (≥ 14) | ≥16 | Case-control study | Omicron (B.1.1.529) | Symptomatic COVID-19 | UK | 71 (42, 86) | 0.29 (0.14, 0.58) |
| Andrews N [97] 2022 | BNT162b2 | After booster dose (≥ 14) | ≥16 | Case-control study | Delta (B.1.617.2) | Symptomatic COVID-19 | UK | 94 (93, 95) | 0.06 (0.05, 0.07) |
| Andrews N [97] 2022 | BNT162b2 | After booster dose (≥ 14) | ≥16 | Case-control study | Omicron (B.1.1.529) | Symptomatic COVID-19 | UK | 76 (56, 86) | 0.24 (0.14, 0.44) |
| Andrews N [97] 2022 | BNT162b2 | After booster dose (≥ 14) | ≥16 | Case-control study | Delta (B.1.617.2) | Symptomatic COVID-19 | UK | 93 (92, 94) | 0.07 (0.06, 0.08) |
| Yoon SK [112] 2022 | BNT16b2 or mRNA-1273 | After booster dose (≥ 7) | > 0 | Cohort study | Delta (B.1.617.2) | SARS-CoV-2 infection | USA | 91 (84–95) | 0.09 (0.05, 0.16) |
| Yoon SK [112] 2022 | BNT16b2 or mRNA-1273 | After booster dose (≥ 7) | > 0 | Cohort study | Omicron (B.1.1.529) | SARS-CoV-2 infection | USA | 60 (42–72) | 0.40 (0.28, 0.58) |
| Buchan SA [98] 2021 | BNT16b2 or mRNA-1273 | After booster dose (≥ 7) | ≥18 | Case-control study | Omicron (B.1.1.529) | Symptomatic COVID-19 | Canada | 61 (56, 65) | 0.39 (0.35, 0.44) |
| Buchan SA [98] 2021 | BNT16b2 or mRNA-1273 | After booster dose (≥ 7) | ≥18 | Case-control study | Delta (B.1.617.2) | Symptomatic COVID-19 | Canada | 97 (96, 98) | 0.03 (0.02, 0.04) |
| Buchan SA [98] 2021 | BNT16b2 | After booster dose (≥ 7) | ≥18 | Case-control study | Omicron (B.1.1.529) | Symptomatic COVID-19 | Canada | 60 (55, 65) | 0.40 (0.35, 0.45) |
| Buchan SA [98] 2021 | BNT16b2 | After booster dose (≥ 7) | ≥18 | Case-control study | Delta (B.1.617.2) | Symptomatic COVID-19 | Canada | 97 (96, 98) | 0.03 (0.02, 0.04) |
| Buchan SA [98] 2021 | mRNA-1273 | After booster dose (≥ 7) | ≥18 | Case-control study | Omicron (B.1.1.529) | Symptomatic COVID-19 | Canada | 65 (55, 72) | 0.35 (0.28, 0.45) |
| Buchan SA [98] 2021 | mRNA-1273 | After booster dose (≥ 7) | ≥18 | Case-control study | Delta (B.1.617.2) | Symptomatic COVID-19 | Canada | 97 (95, 98) | 0.03 (0.02, 0.05) |
| Buchan SA [98] 2021 | BNT16b2 or mRNA-1273 | After booster dose (≥ 7) | ≥18 | Case-control study | Omicron (B.1.1.529) | Severe COVID-19 | Canada | 95 (87, 98) | 0.05 (0.02, 0.13) |
| Buchan SA [98] 2021 | BNT16b2 or mRNA-1273 | After booster dose (≥ 7) | ≥18 | Case-control study | Delta (B.1.617.2) | Severe COVID-19 | Canada | 99 (98, 99) | 0.01 (0.01, 0.02) |
| Buchan SA [98] 2021 | BNT16b2 | After booster dose (≥ 7) | ≥18 | Case-control study | Omicron (B.1.1.529) | Severe COVID-19 | Canada | 95 (87, 98) | 0.05 (0.02, 0.13) |
| Buchan SA [98] 2021 | BNT16b2 | After booster dose (≥ 7) | ≥18 | Case-control study | Delta (B.1.617.2) | Severe COVID-19 | Canada | 99 (98, 99) | 0.01 (0.01, 0.02) |
| Buchan SA [98] 2021 | mRNA-1273 | After booster dose (≥ 7) | ≥18 | Case-control study | Omicron (B.1.1.529) | Severe COVID-19 | Canada | 93 (74, 98) | 0.07 (0.02, 0.26) |
| Buchan SA [98] 2021 | mRNA-1273 | After booster dose (≥ 7) | ≥18 | Case-control study | Delta (B.1.617.2) | Severe COVID-19 | Canada | 99 (98, 99) | 0.01 (0.01, 0.02) |
| Ferdinands JM [85] 2022 | BNT16b2 or mRNA-1273 | After booster dose (≥ 14) | ≥18 | Cohort study | Delta (B.1.617.2) | SARS-CoV-2 infection | USA | 96 (95–96) | 0.04 (0.04, 0.05) |
| Ferdinands JM [85] 2022 | BNT16b2 or mRNA-1273 | After booster dose (≥ 14) | ≥18 | Cohort study | Omicron (B.1.1.529) | SARS-CoV-2 infection | USA | 83 (82–84) | 0.17 (0.16, 0.18) |
| Ferdinands JM [85] 2022 | BNT16b2 or mRNA-1273 | After booster dose (≥ 14) | ≥18 | Cohort study | Delta (B.1.617.2) | Severe COVID-19 | USA | 95 (95–96) | 0.05 (0.04, 0.05) |
| Ferdinands JM [85] 2022 | BNT16b2 or mRNA-1273 | After booster dose (≥ 14) | ≥18 | Cohort study | Omicron (B.1.1.529) | Severe COVID-19 | USA | 88 (86–90) | 0.12 (0.10, 0.14) |
| Kirsebom FCM [111] 2022 | BNT16b2 or mRNA-1273 | After booster dose (≥ 14) | ≥18 | Case-control study | Omicron (B.1.1.529) | Symptomatic COVID-19 | UK | 59 (58, 60) | 0.41 (0.40, 0.42) |
| Kirsebom FCM [111] 2022 | BNT16b2 or mRNA-1273 | After booster dose (≥ 14) | ≥18 | Case-control study | Omicron (BA.2) | Symptomatic COVID-19 | UK | 62 (60, 64) | 0.38 (0.36, 0.40) |
| Gray GE [76] 2021 | Ad26.COV2.S | After booster dose (≥ 14) | ≥18 | Case-control study | Omicron (B.1.1.529) | Severe COVID-19 | South Africa | 85 (61, 94) | 0.15 (0.06, 0.39) |
| Hansen CH [64] 2021 | BNT16b2 | After booster dose (≥ 14) | > 0 | Cohort study | Omicron (B.1.1.529) | SARS-CoV-2 infection | Denmark | 55 (30, 70) | 0.45 (0.30, 0.70) |
| Hansen CH [64] 2021 | BNT16b2 | After booster dose (≥ 14) | > 0 | Cohort study | Delta (B.1.617.2) | SARS-CoV-2 infection | Denmark | 81 (79, 83) | 0.19 (0.17, 0.21) |
| Hansen CH [64] 2021 | mRNA-1273 | After booster dose (≥ 14) | > 0 | Cohort study | Delta (B.1.617.2) | SARS-CoV-2 infection | Denmark | 83 (59; 93) | 0.17 (0.07, 0.41) |
| Kiss Z [108] 2022 | Multiple vaccines | After booster dose (≥ 14) | ≥18 | Cohort study | Delta (B.1.617.2) | COVID-19-related death | Hungary | 96 (95, 96) | 0.04 (0.04, 0.05) |
| Kiss Z [108] 2022 | Multiple vaccines | After booster dose (≥ 14) | ≥18 | Cohort study | Omicron (B.1.1.529) | COVID-19-related death | Hungary | 82 (80, 84) | 0.18 (0.16, 0.20) |
| Kiss Z [108] 2022 | Multiple vaccines | After booster dose (≥ 14) | ≥18 | Cohort study | Omicron (B.1.1.529) | COVID-19-related death | Hungary | 99 (92, 100) | 0.01 (0.00, 0.08) |
| Lauring AS [71] 2022 | BNT16b2 or mRNA-1273 | After booster dose (≥ 14) | ≥18 | Case-control study | Delta (B.1.617.2) | Severe COVID-19 | USA | 94 (92, 95) | 0.06 (0.05, 0.08) |
| Lauring AS [71] 2022 | BNT16b2 or mRNA-1273 | After booster dose (≥ 14) | ≥18 | Case-control study | Delta (B.1.617.2) | Severe COVID-19 | USA | 86 (77, 91) | 0.14 (0.09, 0.23) |
| McMenamin ME [55] 2022 | BNT16b2 | After booster dose (≥ 14) | 20-59 | Cohort study | Omicron (BA.2) | Symptomatic COVID-19 | China | 72 (49, 84) | 0.28 (0.16, 0.51) |
| McMenamin ME [55] 2022 | CoronaVac | After booster dose (≥ 14) | 20-59 | Cohort study | Omicron (BA.2) | Symptomatic COVID-19 | China | 47 (12, 67) | 0.53 (0.33, 0.88) |
| McMenamin ME [55] 2022 | BNT16b2 | After booster dose (≥ 14) | ≥60 | Cohort study | Omicron (BA.2) | Symptomatic COVID-19 | China | 72 (44, 86) | 0.28 (0.14, 0.56) |
| McMenamin ME [55] 2022 | CoronaVac | After booster dose (≥ 14) | ≥60 | Cohort study | Omicron (BA.2) | Symptomatic COVID-19 | China | 51 (13, 72) | 0.49 (0.28, 0.87) |
| McMenamin ME [55] 2022 | CoronaVac | After booster dose (≥ 14) | 20-59 | Cohort study | Omicron (BA.2) | Severe COVID-19 | China | 98.5 (95.2, 99.5) | 0.015 (0.005, 0.048) |
| McMenamin ME [55] 2022 | CoronaVac | After booster dose (≥ 14) | 60-69 | Cohort study | Omicron (BA.2) | Severe COVID-19 | China | 98.5 (95.3, 99.6) | 0.015 (0.004, 0.047) |
| McMenamin ME [55] 2022 | CoronaVac | After booster dose (≥ 14) | 70-79 | Cohort study | Omicron (BA.2) | Severe COVID-19 | China | 96.7 (92.3, 98.6) | 0.033 (0.014, 0.077) |
| McMenamin ME [55] 2022 | CoronaVac | After booster dose (≥ 14) | ≥ 80 | Cohort study | Omicron (BA.2) | Severe COVID-19 | China | 98.6 (94.3, 99.7) | 0.014 (0.003, 0.057) |
| McMenamin ME [55] 2022 | BNT16b2 | After booster dose (≥ 14) | 20-59 | Cohort study | Omicron (BA.2) | Severe COVID-19 | China | 98.5 (95.9, 99.4) | 0.015 (0.006, 0.041) |
| McMenamin ME [55] 2022 | BNT16b2 | After booster dose (≥ 14) | 60-69 | Cohort study | Omicron (BA.2) | Severe COVID-19 | China | 99.2 (96.7, 99.8) | 0.008 (0.002, 0.033) |
| McMenamin ME [55] 2022 | BNT16b2 | After booster dose (≥ 14) | 70-79 | Cohort study | Omicron (BA.2) | Severe COVID-19 | China | 99.5 (96.0, 99.9) | 0.005 (0.001, 0.04) |
| McMenamin ME [55] 2022 | BNT16b2 | After booster dose (≥ 14) | ≥ 80 | Cohort study | Omicron (BA.2) | Severe COVID-19 | China | 95.7 (89.0, 98.3) | 0.043 (0.017, 0.11) |
| McMenamin ME [55] 2022 | CoronaVac | After booster dose (≥ 14) | 60-69 | Cohort study | Omicron (BA.2) | COVID-19-related death | China | 98.7 (94.4, 99.7) | 0.013 (0.003, 0.056) |
| McMenamin ME [55] 2022 | CoronaVac | After booster dose (≥ 14) | 70-79 | Cohort study | Omicron (BA.2) | COVID-19-related death | China | 97.2 (92.3, 99.0) | 0.028 (0.001, 0.077) |
| McMenamin ME [55] 2022 | CoronaVac | After booster dose (≥ 14) | ≥ 80 | Cohort study | Omicron (BA.2) | COVID-19-related death | China | 99.2 (94.3, 99.9) | 0.008 (0.001, 0.057) |
| McMenamin ME [55] 2022 | BNT16b2 | After booster dose (≥ 14) | 20-59 | Cohort study | Omicron (BA.2) | COVID-19-related death | China | 99.4 (95.6, 99.9) | 0.006 (0.001, 0.044) |
| McMenamin ME [55] 2022 | BNT16b2 | After booster dose (≥ 14) | 60-69 | Cohort study | Omicron (BA.2) | COVID-19-related death | China | 98.9 (95.3, 99.7) | 0.011 (0.003, 0.047) |
| McMenamin ME [55] 2022 | BNT16b2 | After booster dose (≥ 14) | ≥ 80 | Cohort study | Omicron (BA.2) | COVID-19-related death | China | 96.0 (88.8, 98.6) | 0.04 (0.014, 0.112) |
| Nordström P [94] 2021 | BNT16b2 | After booster dose (≥ 14) | > 0 | Cohort study | Delta (B.1.617.2) | Symptomatic COVID-19 | Sweden | 78 (77, 80) | 0.22 (0.20, 0.23) |
| Nordström P [94] 2021 | BNT162b2 | After booster dose (≥ 14) | > 0 | Cohort study | Delta (B.1.617.2) | Symptomatic COVID-19 | Sweden | 67 (59, 73) | 0.33 (0.27, 0.41) |
| Nordström P [94] 2021 | ChAdOx1 nCoV-19 | After booster dose (≥ 14) | > 0 | Cohort study | Delta (B.1.617.2) | Symptomatic COVID-19 | Sweden | 50 (41, 58) | 0.50 (0.42, 0.59) |
| Nordström P [94] 2021 | mRNA-1273 | After booster dose (≥ 14) | > 0 | Cohort study | Delta (B.1.617.2) | Symptomatic COVID-19 | Sweden | 79 (62, 88) | 0.21 (0.12, 0.38) |
| Nordström P [94] 2021 | mRNA-1273 | After booster dose (≥ 14) | > 0 | Cohort study | Delta (B.1.617.2) | Symptomatic COVID-19 | Sweden | 87 (84, 88) | 0.13 (0.12, 0.16) |
| Nunes MC [77] 2022 | Ad26.COV2.S | After booster dose (≥ 14) | > 0 | Case-control study | Omicron (B.1.1.529) | SARS-CoV-2 infection | South Africa | 6 (-103, 56) | 0.94 (0.44, 2.03) |
| Poukka E [102] 2021 | Ad26.COV2.S | After booster dose (≥ 14) | 16-70 | Cohort study | Delta (B.1.617.2) | SARS-CoV-2 infection | Finland | 88 (71, 95) | 0.12 (0.05, 0.29) |
| Poukka E [102] 2021 | Ad26.COV2.S, BNT16b2, or mRNA-1273 | After booster dose (≥ 14) | 16-70 | Cohort study | Delta (B.1.617.2) | SARS-CoV-2 infection | Finland | 80 (72, 86) | 0.20 (0.14, 0.28) |
| Šmíd M [63] 2022 | Multiple vaccines | After booster dose (≥ 14) | > 0 | Cohort study | Delta (B.1.617.2) | SARS-CoV-2 infection | Czech | 90 (89, 91) | 0.10 (0.09, 0.11) |
| Šmíd M [63] 2022 | Multiple vaccines | After booster dose (≥ 14) | > 0 | Cohort study | Omicron (B.1.1.529) | SARS-CoV-2 infection | Czech | 56 (55, 57) | 0.44 (0.43, 0.45) |
| Šmíd M [63] 2022 | Multiple vaccines | After booster dose (≥ 14) | > 0 | Cohort study | Omicron (B.1.1.529) | Severe COVID-19 | Czech | 87 (84, 88) | 0.13 (0.12, 0.16) |
| Spensley K [99] 2022 | ChAdOx1 nCoV-19 | After booster dose (≥ 14) | > 0 | Cohort study | Omicron (B.1.1.529) | SARS-CoV-2 infection | UK | 47 (2, 70) | 0.53 (0.30, 0.98) |
| Spensley K [99] 2022 | BNT16b2 | After booster dose (≥ 14) | > 0 | Cohort study | Omicron (B.1.1.529) | SARS-CoV-2 infection | UK | 66 (36, 81) | 0.34 (0.19, 0.64) |
| Sritipsukho P [81] 2022 | BNT162b2 | After booster dose (≥ 7) | ≥18 | Case-control study | Delta (B.1.617.2) | SARS-CoV-2 infection | Thailand | 98 (87, 99) | 0.02 (0.01, 0.13) |
| Sritipsukho P [81] 2022 | ChAdOx1 nCoV-19 | After booster dose (≥ 7) | ≥18 | Case-control study | Delta (B.1.617.2) | SARS-CoV-2 infection | Thailand | 86 (74, 93) | 0.14 (0.07, 0.26) |
| Starrfelt J [109] 2022 | BNT16b2 | After booster dose (≥ 7) | ≥18 | Cohort study | Delta (B.1.617.2) | SARS-CoV-2 infection | Norway | 75 (73, 78) | 0.25 (0.22, 0.27) |
| Starrfelt J [109] 2022 | mRNA-1273 | After booster dose (≥ 7) | ≥18 | Cohort study | Delta (B.1.617.2) | SARS-CoV-2 infection | Norway | 68 (58, 76) | 0.32 (0.24, 0.42) |
| Starrfelt J [109] 2022 | BNT16b2 | After booster dose (≥ 7) | ≥18 | Cohort study | Delta (B.1.617.2) | Severe COVID-19 | Norway | 96 (93, 97) | 0.04 (0.03, 0.07) |
| Starrfelt J [109] 2022 | mRNA-1273 | After booster dose (≥ 7) | ≥18 | Cohort study | Delta (B.1.617.2) | Severe COVID-19 | Norway | 74 (46, 87) | 0.26 (0.13, 0.54) |
| Starrfelt J [109] 2022 | Any 3 doses of mRNA vaccine | After booster dose (≥ 7) | ≥18 | Cohort study | Delta (B.1.617.2) | COVID-19-related death | Norway | 96 (93, 98) | 0.04 (0.02, 0.07) |
| Tenforde MW [95] 2022 | Any 3 doses of mRNA vaccine | After booster dose (≥ 14) | ≥18 | Case-control study | Delta (B.1.617.2) | SARS-CoV-2 infection | USA | 97 (95, 99) | 0.03 (0.01, 0.05) |
| Thompson MG [91] 2022 | Any 3 doses of mRNA vaccine | After booster dose (≥ 14) | ≥18 | Cohort study | Delta (B.1.617.2) | Severe COVID-19 | USA | 94 (93, 94) | 0.06 (0.06, 0.07) |
| Thompson MG [91] 2022 | Any 3 doses of mRNA vaccine | After booster dose (≥ 14) | ≥18 | Cohort study | Omicron (B.1.1.529) | Severe COVID-19 | USA | 82 (79, 84) | 0.18 (0.16, 0.21) |
| Tseng HF [88] 2022 | mRNA-1273 | After booster dose (≥ 14) | ≥18 | Case-control study | Delta (B.1.617.2) | SARS-CoV-2 infection | USA | 95 (93, 96) | 0.05 (0.04, 0.07) |
| Tseng HF [88] 2022 | mRNA-1273 | After booster dose (≥ 14) | ≥18 | Case-control study | Omicron (B.1.1.529) | SARS-CoV-2 infection | USA | 70 (68, 72) | 0.30 (0.28, 0.32) |
| Tseng HF [88] 2022 | mRNA-1273 | After booster dose (≥ 14) | ≥18 | Case-control study | Delta (B.1.617.2) | Severe COVID-19 | USA | 98 (97, 99) | 0.02 (0.01, 0.03) |
| Tseng HF [88] 2022 | mRNA-1273 | After booster dose (≥ 14) | ≥18 | Case-control study | Omicron (B.1.1.529) | Severe COVID-19 | USA | 98 (76, 99) | 0.02 (0.01, 0.24) |
| Yinong [73] 2022 | BNT162b2 or mRNA-1273 | After booster dose (≥ 14) | ≥18 | Case-control study | Delta (B.1.617.2) | SARS-CoV-2 infection | USA | 90 (88, 92) | 0.10 (0.08, 0.12) |
| Yinong [73] 2022 | BNT162b2 or mRNA-1273 | After booster dose (≥ 14) | ≥18 | Case-control study | Omicron (B.1.1.529) | SARS-CoV-2 infection | USA | 59 (57, 61) | 0.41 (0.39, 0.43) |
| Yinong [73] 2022 | BNT162b2 or mRNA-1273 | After booster dose (≥ 14) | ≥18 | Case-control study | Delta (B.1.617.2) | COVID-19-related death | USA | 96 (88, 99) | 0.04 (0.01, 0.12) |
| Yinong [73] 2022 | BNT162b2 or mRNA-1273 | After booster dose (≥ 14) | ≥18 | Case-control study | Omicron (B.1.1.529) | COVID-19-related death | USA | 94 (85, 98) | 0.06 (0.02, 0.15) |
| Yinong [73] 2022 | BNT162b2 or mRNA-1273 | After booster dose (≥ 14) | ≥18 | Case-control study | Delta (B.1.617.2) | Severe COVID-19 | USA | 95 (91, 97) | 0.05 (0.03, 0.09) |
| Yinong [73] 2022 | BNT162b2 or mRNA-1273 | After booster dose (≥ 14) | ≥18 | Case-control study | Omicron (B.1.1.529) | Severe COVID-19 | USA | 87 (80, 91) | 0.13 (0.09, 0.20) |
| **Comparison between booster vaccinees and non-booster vaccinees groups** | | | | | | | | | |
| Accorsi EK [89] 2022 | Any 3 doses of mRNA vaccine | After booster dose (≥ 14) | ≥18 | Case-control study | Delta (B.1.617.2) | Symptomatic COVID-19 | USA | 84 (83, 86) | 0.16 (0.14, 0.17) |
| Accorsi EK [89] 2022 | Any 3 doses of mRNA vaccine | After booster dose (≥ 14) | ≥18 | Case-control study | Omicron (B.1.1.529) | Symptomatic COVID-19 | USA | 66 (64, 68) | 0.34 (0.32, 0.36) |
| Accorsi EK [89] 2022 | BNT16b2 | After booster dose (≥ 14) | ≥18 | Case-control study | Delta (B.1.617.2) | Symptomatic COVID-19 | USA | 83 (81, 84) | 0.17 (0.16, 0.19) |
| Accorsi EK [89] 2022 | BNT16b2 | After booster dose (≥ 14) | ≥18 | Case-control study | Omicron (B.1.1.529) | Symptomatic COVID-19 | USA | 65 (63, 68) | 0.35 (0.32, 0.37) |
| Accorsi EK [89] 2022 | mRNA-1273 | After booster dose (≥ 14) | ≥18 | Case-control study | Delta (B.1.617.2) | Symptomatic COVID-19 | USA | 87 (85, 89) | 0.13 (0.11, 0.15) |
| Accorsi EK [89] 2022 | mRNA-1273 | After booster dose (≥ 14) | ≥18 | Case-control study | Omicron (B.1.1.529) | Symptomatic COVID-19 | USA | 69 (66, 72) | 0.31 (0.28, 0.34) |
| Barda N [93] 2021 | BNT16b2 | After booster dose (≥ 7) | ≥16 | Cohort study | Delta (B.1.617.2) | SARS-CoV-2 infection | Israel | 88 (87–90) | 0.12 (0.10, 0.13) |
| Barda N [93] 2021 | BNT16b2 | After booster dose (≥ 7) | ≥16 | Cohort study | Delta (B.1.617.2) | Symptomatic COVID-19 | Israel | 91 (89, 92) | 0.09 (0.08, 0.11) |
| Barda N [93] 2021 | BNT16b2 | After booster dose (≥ 7) | ≥16 | Cohort study | Delta (B.1.617.2) | Severe COVID-19 | Israel | 89 (83, 94) | 0.11 (0.06, 0.17) |
| Barda N [93] 2021 | BNT16b2 | After booster dose (≥ 7) | ≥16 | Cohort study | Delta (B.1.617.2) | COVID-19-related death | Israel | 84 (67, 93) | 0.16 (0.07, 0.33) |
| McMenamin ME [55] 2022 | BNT162b2 | After booster dose (≥ 14) | ≥20 | Cohort study | Omicron (BA.2) | Symptomatic COVID-19 | UK | 48 (32, 60) | 0.52 (0.40, 0.68) |
| McMenamin ME [55] 2022 | BNT162b2 | After booster dose (≥ 14) | ≥70 | Cohort study | Omicron (BA.2) | Symptomatic COVID-19 | UK | 71 (43, 85) | 0.29 (0.15, 0.57) |
| McMenamin ME [55] 2022 | BNT162b2 | After booster dose (≥ 14) | ≥20 | Cohort study | Omicron (BA.2) | Severe COVID-19 | UK | 87 (81, 92) | 0.13 (0.08, 0.19) |
| McMenamin ME [55] 2022 | BNT162b2 | After booster dose (≥ 14) | ≥70 | Cohort study | Omicron (BA.2) | Severe COVID-19 | UK | 89 (81, 94) | 0.11 (0.06, 0.19) |
| McMenamin ME [55] 2022 | BNT162b2 | After booster dose (≥ 14) | ≥20 | Cohort study | Omicron (BA.2) | COVID-19-related death | UK | 89.0 (81.4, 94.0) | 0.11 (0.06, 0.186) |
| McMenamin ME [55] 2022 | BNT162b2 | After booster dose (≥ 14) | ≥70 | Cohort study | Omicron (BA.2) | COVID-19-related death | UK | 89.6 (79.2, 94.8) | 0.104 (0.052, 0.208) |
| Norddahl GL [70] 2022 | BNT16b2 | After booster dose (≥ 14) | ≥18 | Cohort study | Omicron (B.1.1.529) | SARS-CoV-2 infection | Iceland | 47 (36, 56) | 0.53 (0.44-0.64) |
| Norddahl GL [70] 2022 | mRNA-1273 | After booster dose (≥ 14) | ≥18 | Cohort study | Omicron (B.1.1.529) | SARS-CoV-2 infection | Iceland | 50 (34, 62) | 0.50 (0.38-0.66) |
| Norddahl GL [70] 2022 | BNT16b2 | After booster dose (≥ 14) | ≥18 | Cohort study | Delta (B.1.617.2) | SARS-CoV-2 infection | Iceland | 52 (28, 69) | 0.48 (0.31-0.72) |
| Norddahl GL [70] 2022 | mRNA-1273 | After booster dose (≥ 14) | ≥18 | Cohort study | Delta (B.1.617.2) | SARS-CoV-2 infection | Iceland | 73 (29, 90) | 0.27 (0.10-0.71) |
| Saciuk Y [92] 2022 | BNT162b2 | After booster dose (≥ 7) | > 0 | Cohort study | Delta (B.1.617.2) | SARS-CoV-2 infection | Israel | 89 (88, 91) | 0.11 (0.09, 0.12) |
| Spitzer A [90] 2022 | BNT162b2 | After booster dose (≥ 7) | > 0 | Cohort study | Delta (B.1.617.2) | SARS-CoV-2 infection | Israel | 93 (80, 98) | 0.07 (0.02, 0.20) |
| Spitzer A [90] 2022 | BNT162b2 | After booster dose (≥ 7) | > 0 | Cohort study | Delta (B.1.617.2) | Symptomatic COVID-19 | Israel | 93 (75, 98) | 0.07 (0.02, 0.25) |
| Spitzer A [90] 2022 | BNT162b2 | After booster dose (≥ 7) | > 0 | Cohort study | Delta (B.1.617.2) | Asymptomatic infection | Israel | 92 (52, 99) | 0.08 (0.01, 0.48) |
| Tai CG [100] 2022 | BNT162b2 or mRNA-1273 | After booster dose (≥ 14) | > 0 | Cohort study | Omicron (B.1.1.529) | SARS-CoV-2 infection | USA | 64 (57, 70) | 0.36 (0.30, 0.43) |
| Tai CG [100] 2022 | Ad26.COV2.S | After booster dose (≥ 14) | > 0 | Cohort study | Omicron (B.1.1.529) | SARS-CoV-2 infection | USA | 57 (44, 66) | 0.43 (0.34, 0.56) |
| Tan SHX [96] 2022 | BNT162b2 | After booster dose (≥ 14) | ≥ 60 | Cohort study | Delta (B.1.617.2) | SARS-CoV-2 infection | Singapore | 73 (71, 74) | 0.27 (0.26, 0.29) |
| Tan SHX [96] 2022 | mRNA-1273 | After booster dose (≥ 14) | ≥ 60 | Cohort study | Delta (B.1.617.2) | SARS-CoV-2 infection | Singapore | 82 (77, 86) | 0.18 (0.14, 0.23) |
| Tan SHX [96] 2022 | mRNA-1273 | After booster dose (≥ 14) | ≥ 60 | Cohort study | Delta (B.1.617.2) | SARS-CoV-2 infection | Singapore | 80 (73, 86) | 0.20 (0.14, 0.27) |
| Tan SHX [96] 2022 | BNT162b2 | After booster dose (≥ 14) | ≥ 60 | Cohort study | Delta (B.1.617.2) | SARS-CoV-2 infection | Singapore | 86 (62, 95) | 0.14 (0.05, 0.38) |
| Tan SHX [96] 2022 | BNT162b2 | After booster dose (≥ 14) | ≥ 60 | Cohort study | Delta (B.1.617.2) | Severe COVID-19 | Singapore | 95 (92, 97) | 0.05 (0.03, 0.08) |
| Tan SHX [96] 2022 | mRNA-1273 | After booster dose (≥ 14) | ≥ 60 | Cohort study | Delta (B.1.617.2) | Severe COVID-19 | Singapore | 92 (44, 99) | 0.08 (0.01, 0.56) |
| Yoon SK [112] 2022 | BNT162b2 or mRNA-1273 | After booster dose (≥ 7) | > 0 | Cohort study | Delta (B.1.617.2) | SARS-CoV-2 infection | USA | 86 (69–94) | 0.14 (0.06, 0.31) |
| Yoon SK [112] 2022 | BNT162b2 or mRNA-1273 | After booster dose (≥ 7) | > 0 | Cohort study | Omicron (B.1.1.529) | SARS-CoV-2 infection | USA | 60 (40–73) | 0.40 (0.27, 0.60) |
| **Comparison between two doses of COVID-19 vaccine booster immunization and one dose of COVID-19 vaccine booster immunization groups** | | | | | | | | | |
| Cohen MJ [118] 2022 | BNT16b2 | After dose 4 (> 7) | ≥18 | Cohort study | Omicron (B.1.1.529) | SARS-CoV-2 infection | Israel | 44 (37, 50) | 0.56 (0.50, 0.63) |
| Regev-Yochay G [67] 2021 | BNT16b2 | After dose 4 (> 7) | ≥18 | Non-RCT study | Omicron (B.1.1.529) | SARS-CoV-2 infection | Israel | 30 (-9, 55) | 0.70 (0.45, 1.09) |
| Regev-Yochay G [67] 2021 | BNT16b2 | After dose 4 (> 7) | ≥18 | Non-RCT study | Omicron (B.1.1.529) | Symptomatic COVID-19 | Israel | 43 (7, 65) | 0.57 (0.35, 0.93) |
| Regev-Yochay G [67] 2021 | mRNA-1273 | After dose 4 (> 7) | ≥18 | Non-RCT study | Omicron (B.1.1.529) | SARS-CoV-2 infection | Israel | 11 (-43, 44) | 0.89 (0.56, 1.43) |
| Regev-Yochay G [67] 2021 | mRNA-1273 | After dose 4 (> 7) | ≥18 | Non-RCT study | Omicron (B.1.1.529) | Symptomatic COVID-19 | Israel | 31 (-18, 60) | 0.69 (0.40, 1.18) |
| Bar-On YM [72] 2022 | BNT162b2 | After dose 4 (≥ 12) | ≥ 60 | Cohort study | Omicron (B.1.1.529) | SARS-CoV-2 infection | Israel | 50 (50, 52) | 0.5 (0.48, 0.50) |
| Bar-On YM [72] 2022 | BNT162b2 | After dose 4 (≥ 12) | ≥ 60 | Cohort study | Omicron (B.1.1.529) | Severe COVID-19 | Israel | 77 (58, 87) | 0.23 (0.13, 0.42) |
| Kiss Z [108] 2022 | Multiple vaccines | After dose 4 (≥ 14) | ≥18 | Cohort study | Omicron (B.1.1.529) | COVID-19-related death | Hungary | 93 (54, 99) | 0.07 (0.01, 0.46) |
| Kiss Z [108] 2022 | Multiple vaccines | After dose 4 (≥ 14) | ≥18 | Cohort study | Omicron (B.1.1.529) | SARS-CoV-2 infection | Hungary | 51 (47, 56) | 0.49 (0.44, 0.53) |

VE: vaccine effectiveness/efficacy; booster vaccination as ≥ 7, 12, or 14 days after the third dose vaccination for COVID-19 vaccines BNT162b2, mRNA-1273, ChAdOx1 nCoV-19, and CoronaVac and ≥ 14 days after the second dose vaccination for COVID-19 vaccines Ad26.COV2.S.
