# Supplementary material for: A comprehensive analysis of the efficacy and effectiveness of COVID-19 vaccines
Source: Front Immunol. 2022 Aug 26;13:945930. doi: 10.3389/fimmu.2022.945930 (PMC9459021; doi:10.3389/fimmu.2022.945930)
Supplement: Supplementary file 6 [file Table_5.docx]

**Supplementary Table 5** Characteristics of studies of breakthrough symptomatic COVID-19 and SARS-CoV-2 infection during the delta variant period

| **First author/year** | **Country** | **Type of study** | **Study population** | **Vaccine name** | **Age (year)** | **Type of cases** | **During variant Period/Variant** | **Dose and weeks since final dose** | **Case of number** | **Total number of persons or no. person-years** | **Risk or rate per 1000** |
| --- | --- | --- | --- | --- | --- | --- | --- | --- | --- | --- | --- |
| Baden LR [51] 2021 | USA | RCT | 11431 | mRNA-1273 | ≥18 | Symptomatic COVID-19 | Delta (B.1.617.2) | After dose 2 (26-34) | 88 | 1796 | 49 |
| Baden LR [51] 2021 | USA | RCT | 14746 | mRNA-1273 | ≥18 | Symptomatic COVID-19 | Delta (B.1.617.2) | After dose 2 (47-56) | 162 | 2102 | 77.07 |
| US FDA [48] 2022 | Multiple | RCT | 36442 | BNT162b2 | ≥18 | Symptomatic COVID-19 | Delta (B.1.617.2) | After dose 2 (1-17) | 26 | 598 | 43.48 |
| US FDA [48] 2022 | Multiple | RCT | 36442 | BNT162b2 | ≥18 | Symptomatic COVID-19 | Delta (B.1.617.2) | After dose 2 (17-26) | 108 | 2019 | 53.49 |
| US FDA [48] 2022 | Multiple | RCT | 36442 | BNT162b2 | ≥18 | Symptomatic COVID-19 | Delta (B.1.617.2) | After dose 2 (26-34) | 19 | 327 | 58.1 |
| US FDA [48] 2022 | Multiple | RCT | 36442 | BNT162b2 | ≥18 | Symptomatic COVID-19 | Delta (B.1.617.2) | After dose 2 (34-43) | 73 | 951 | 76.76 |
| US FDA [48] 2022 | Multiple | RCT | 36442 | BNT162b2 | ≥18 | Symptomatic COVID-19 | Delta (B.1.617.2) | After dose 2 (≥ 43) | 86 | 1282 | 67.08 |
| Baden LR [51] 2021 | USA | RCT | 11431 | mRNA-1273 | 18-65 | Symptomatic COVID-19 | Delta (B.1.617.2) | After dose 2 (26-34) | 68 | 1289 | 52.75 |
| Baden LR [51] 2021 | USA | RCT | 11431 | mRNA-1273 | ≥65 | Symptomatic COVID-19 | Delta (B.1.617.2) | After dose 2 (26-34) | 20 | 507 | 39.45 |
| Baden LR [51] 2021 | USA | RCT | 14746 | mRNA-1273 | 18-65 | Symptomatic COVID-19 | Delta (B.1.617.2) | After dose 2 (330-389) | 136 | 1558 | 87.29 |
| Baden LR [51] 2021 | USA | RCT | 14746 | mRNA-1273 | ≥65 | Symptomatic COVID-19 | Delta (B.1.617.2) | After dose 2 (330-389) | 26 | 544 | 47.79 |
| Rosenberg ES [115] 2022 | USA | Cohort study | 6394228 | BNT162b2 | 18-49 | Symptomatic COVID-19 | Delta (B.1.617.2) | After full vaccination (1-21) | 5858 | 571755 | 10.25 |
| Rosenberg ES [115] 2022 | USA | Cohort study | 6394228 | BNT162b2 | 50-64 | Symptomatic COVID-19 | Delta (B.1.617.2) | After full vaccination (1-21) | 3382 | 559209 | 6.048 |
| Rosenberg ES [115] 2022 | USA | Cohort study | 6394228 | BNT162b2 | ≥65 | Symptomatic COVID-19 | Delta (B.1.617.2) | After full vaccination (1-21) | 1898 | 427979 | 4.435 |
| Rosenberg ES [115] 2022 | USA | Cohort study | 6394228 | mRNA­1273 | 18-49 | Symptomatic COVID-19 | Delta (B.1.617.2) | After full vaccination (1-21) | 1984 | 306215 | 6.479 |
| Rosenberg ES [115] 2022 | USA | Cohort study | 6394228 | mRNA­1273 | 50-64 | Symptomatic COVID-19 | Delta (B.1.617.2) | After full vaccination (1-21) | 1149 | 338352 | 3.396 |
| Rosenberg ES [115] 2022 | USA | Cohort study | 6394228 | mRNA­1273 | ≥65 | Symptomatic COVID-19 | Delta (B.1.617.2) | After full vaccination (1-21) | 1189 | 437431 | 2.718 |
| Rosenberg ES [115] 2022 | USA | Cohort study | 6394228 | Ad26.COV2.S | 18-49 | Symptomatic COVID-19 | Delta (B.1.617.2) | After full vaccination (1-21) | 2450 | 217706 | 11.25 |
| Rosenberg ES [115] 2022 | USA | Cohort study | 6394228 | Ad26.COV2.S | 50-64 | Symptomatic COVID-19 | Delta (B.1.617.2) | After full vaccination (1-21) | 910 | 126627 | 7.186 |
| Rosenberg ES [115] 2022 | USA | Cohort study | 6394228 | Ad26.COV2.S | ≥65 | Symptomatic COVID-19 | Delta (B.1.617.2) | After full vaccination (1-21) | 405 | 65182 | 6.213 |
| Rosenberg ES [115] 2022 | USA | Cohort study | 6394228 | BNT162b2 | 18-49 | Symptomatic COVID-19 | Delta (B.1.617.2) | After full vaccination (4-26) | 1822 | 151525 | 12.02 |
| Rosenberg ES [115] 2022 | USA | Cohort study | 6394228 | BNT162b2 | 50-64 | Symptomatic COVID-19 | Delta (B.1.617.2) | After full vaccination (4-26) | 1068 | 135138 | 7.903 |
| Rosenberg ES [115] 2022 | USA | Cohort study | 6394228 | BNT162b2 | ≥65 | Symptomatic COVID-19 | Delta (B.1.617.2) | After full vaccination (4-26) | 1961 | 343396 | 5.711 |
| Rosenberg ES [115] 2022 | USA | Cohort study | 6394228 | mRNA­1273 | 18-49 | Symptomatic COVID-19 | Delta (B.1.617.2) | After full vaccination (4-26) | 1430 | 174833 | 8.179 |
| Rosenberg ES [115] 2022 | USA | Cohort study | 6394228 | mRNA­1273 | 50-64 | Symptomatic COVID-19 | Delta (B.1.617.2) | After full vaccination (4-26) | 656 | 120666 | 5.436 |
| Rosenberg ES [115] 2022 | USA | Cohort study | 6394228 | mRNA­1273 | ≥65 | Symptomatic COVID-19 | Delta (B.1.617.2) | After full vaccination (4-26) | 1439 | 426802 | 3.372 |
| Rosenberg ES [115] 2022 | USA | Cohort study | 6394228 | Ad26.COV2.S | 18-49 | Symptomatic COVID-19 | Delta (B.1.617.2) | After full vaccination (4-26) | 818 | 50350 | 16.25 |
| Rosenberg ES [115] 2022 | USA | Cohort study | 6394228 | Ad26.COV2.S | 50-64 | Symptomatic COVID-19 | Delta (B.1.617.2) | After full vaccination (4-26) | 489 | 54060 | 9.046 |
| Rosenberg ES [115] 2022 | USA | Cohort study | 6394228 | Ad26.COV2.S | ≥65 | Symptomatic COVID-19 | Delta (B.1.617.2) | After full vaccination (4-26) | 342 | 49109 | 6.964 |
| Rosenberg ES [115] 2022 | USA | Cohort study | 6394228 | BNT162b2 | 18-49 | Symptomatic COVID-19 | Delta (B.1.617.2) | After full vaccination (7-34) | 2924 | 217159 | 13.46 |
| Rosenberg ES [115] 2022 | USA | Cohort study | 6394228 | BNT162b2 | 50-64 | Symptomatic COVID-19 | Delta (B.1.617.2) | After full vaccination (7-34) | 1107 | 131153 | 8.441 |
| Rosenberg ES [115] 2022 | USA | Cohort study | 6394228 | BNT162b2 | ≥65 | Symptomatic COVID-19 | Delta (B.1.617.2) | After full vaccination (7-34) | 1411 | 196823 | 7.169 |
| Rosenberg ES [115] 2022 | USA | Cohort study | 6394228 | mRNA­1273 | 18-49 | Symptomatic COVID-19 | Delta (B.1.617.2) | After full vaccination (7-34) | 2183 | 245546 | 8.89 |
| Rosenberg ES [115] 2022 | USA | Cohort study | 6394228 | mRNA­1273 | 50-64 | Symptomatic COVID-19 | Delta (B.1.617.2) | After full vaccination (7-34) | 900 | 149357 | 6.026 |
| Rosenberg ES [115] 2022 | USA | Cohort study | 6394228 | mRNA­1273 | ≥65 | Symptomatic COVID-19 | Delta (B.1.617.2) | After full vaccination (7-34) | 644 | 141769 | 4.543 |
| Starrfelt J [109] 2022 | Norway | Cohort study | 128982 | Multiple vaccines | ≥18 | SARS-CoV-2 infection | Delta (B.1.617.2) | After dose 2 (2-10) | 5528 | 377421 | 14.65 |
| Starrfelt J [109] 2022 | Norway | Cohort study | 128982 | Multiple vaccines | ≥18 | SARS-CoV-2 infection | Delta (B.1.617.2) | After dose 2 (10-18) | 17189 | 389106 | 44.18 |
| Starrfelt J [109] 2022 | Norway | Cohort study | 128982 | Multiple vaccines | ≥18 | SARS-CoV-2 infection | Delta (B.1.617.2) | After dose 2 (18-26) | 9985 | 218114 | 45.78 |
| Starrfelt J [109] 2022 | Norway | Cohort study | 128982 | Multiple vaccines | ≥18 | SARS-CoV-2 infection | Delta (B.1.617.2) | After dose 2 (26-33) | 5233 | 80587 | 64.94 |
| Starrfelt J [109] 2022 | Norway | Cohort study | 128982 | Multiple vaccines | ≥18 | SARS-CoV-2 infection | Delta (B.1.617.2) | After dose 2 (≥ 33) | 2174 | 23851 | 91.15 |
| Starrfelt J [109] 2022 | Norway | Cohort study | 165757 | Multiple vaccines | ≥18 | SARS-CoV-2 infection | Delta (B.1.617.2) | After dose 2 (2-10) | 5003 | 327953 | 15.26 |
| Starrfelt J [109] 2022 | Norway | Cohort study | 165757 | Multiple vaccines | ≥18 | SARS-CoV-2 infection | Delta (B.1.617.2) | After dose 2 (10-18) | 15094 | 293973 | 51.34 |
| Starrfelt J [109] 2022 | Norway | Cohort study | 165757 | Multiple vaccines | ≥18 | SARS-CoV-2 infection | Delta (B.1.617.2) | After dose 2 (18-26) | 6512 | 129799 | 50.17 |
| Starrfelt J [109] 2022 | Norway | Cohort study | 165757 | Multiple vaccines | ≥18 | SARS-CoV-2 infection | Delta (B.1.617.2) | After dose 2 (26-33) | 3396 | 45235 | 75.07 |
| Starrfelt J [109] 2022 | Norway | Cohort study | 165757 | Multiple vaccines | ≥18 | SARS-CoV-2 infection | Delta (B.1.617.2) | After dose 2 (≥ 33) | 1478 | 13724 | 107.7 |
| Goldberg Y [106] 2021 | Israel | Cohort study | 936 711 | BNT162b2 | 16-39 | SARS-CoV-2 infection | Delta (B.1.617.2) | After dose 2 (6–17) | 401 | 284983 | 1.407 |
| Goldberg Y [106] 2021 | Israel | Cohort study | 936 711 | BNT162b2 | 16-39 | SARS-CoV-2 infection | Delta (B.1.617.2) | After dose 2 (15-20) | 988 | 496779 | 1.989 |
| Goldberg Y [106] 2021 | Israel | Cohort study | 936 711 | BNT162b2 | 16-39 | SARS-CoV-2 infection | Delta (B.1.617.2) | After dose 2 (18–22) | 1474 | 549090 | 2.684 |
| Goldberg Y [106] 2021 | Israel | Cohort study | 936 711 | BNT162b2 | 16-39 | SARS-CoV-2 infection | Delta (B.1.617.2) | After dose 2 (19–24) | 1060 | 352722 | 3.005 |
| Goldberg Y [106] 2021 | Israel | Cohort study | 936 711 | BNT162b2 | 16-39 | SARS-CoV-2 infection | Delta (B.1.617.2) | After dose 2 (21–26) | 751 | 195961 | 3.832 |
| Goldberg Y [106] 2021 | Israel | Cohort study | 936 711 | BNT162b2 | 16-39 | SARS-CoV-2 infection | Delta (B.1.617.2) | After dose 2 (23–28) | 464 | 125977 | 3.683 |
| Goldberg Y [106] 2021 | Israel | Cohort study | 936 711 | BNT162b2 | 40-59 | SARS-CoV-2 infection | Delta (B.1.617.2) | After dose 2 (6–17) | 149 | 100511 | 1.482 |
| Goldberg Y [106] 2021 | Israel | Cohort study | 936 711 | BNT162b2 | 40-59 | SARS-CoV-2 infection | Delta (B.1.617.2) | After dose 2 (15-20) | 366 | 190326 | 1.923 |
| Goldberg Y [106] 2021 | Israel | Cohort study | 936 711 | BNT162b2 | 40-59 | SARS-CoV-2 infection | Delta (B.1.617.2) | After dose 2 (18–22) | 565 | 208064 | 2.716 |
| Goldberg Y [106] 2021 | Israel | Cohort study | 936 711 | BNT162b2 | 40-59 | SARS-CoV-2 infection | Delta (B.1.617.2) | After dose 2 (19–24) | 1051 | 328038 | 3.204 |
| Goldberg Y [106] 2021 | Israel | Cohort study | 936 711 | BNT162b2 | 40-59 | SARS-CoV-2 infection | Delta (B.1.617.2) | After dose 2 (21–26) | 1458 | 418282 | 3.486 |
| Goldberg Y [106] 2021 | Israel | Cohort study | 936 711 | BNT162b2 | 40-59 | SARS-CoV-2 infection | Delta (B.1.617.2) | After dose 2 (23–28) | 967 | 243741 | 3.967 |
| Goldberg Y [106] 2021 | Israel | Cohort study | 936 711 | BNT162b2 | ≥60 | SARS-CoV-2 infection | Delta (B.1.617.2) | After dose 2 (6–17) | 51 | 40111 | 1.271 |
| Goldberg Y [106] 2021 | Israel | Cohort study | 936 711 | BNT162b2 | ≥60 | SARS-CoV-2 infection | Delta (B.1.617.2) | After dose 2 (15-20) | 105 | 62317 | 1.685 |
| Goldberg Y [106] 2021 | Israel | Cohort study | 936 711 | BNT162b2 | ≥60 | SARS-CoV-2 infection | Delta (B.1.617.2) | After dose 2 (18–22) | 107 | 61886 | 1.729 |
| Goldberg Y [106] 2021 | Israel | Cohort study | 936 711 | BNT162b2 | ≥60 | SARS-CoV-2 infection | Delta (B.1.617.2) | After dose 2 (19–24) | 148 | 67028 | 2.208 |
| Goldberg Y [106] 2021 | Israel | Cohort study | 936 711 | BNT162b2 | ≥60 | SARS-CoV-2 infection | Delta (B.1.617.2) | After dose 2 (21–26) | 973 | 358592 | 2.713 |
| Goldberg Y [106] 2021 | Israel | Cohort study | 936 711 | BNT162b2 | ≥60 | SARS-CoV-2 infection | Delta (B.1.617.2) | After dose 2 (23–28) | 2348 | 706990 | 3.321 |
| Israel A [114] 2021 | Israel | Cohort study | 33 993 | BNT162b2 | ≥18 | SARS-CoV-2 infection | Delta (B.1.617.2) | After dose 2 (3-13) | 6320 | 37290 | 169.5 |
| Mizrahi B [113] 2021 | Israel | Cohort study | 1352444 | BNT162b2 | ≥16 | SARS-CoV-2 infection | Delta (B.1.617.2) | After dose 2 (9-13) | 76 | 44734 | 1.699 |
| Mizrahi B [113] 2021 | Israel | Cohort study | 1352444 | BNT162b2 | ≥16 | SARS-CoV-2 infection | Delta (B.1.617.2) | After dose 2 (13-17) | 858 | 371929 | 2.307 |
| Mizrahi B [113] 2021 | Israel | Cohort study | 1352444 | BNT162b2 | ≥16 | SARS-CoV-2 infection | Delta (B.1.617.2) | After dose 2 (17-21) | 1550 | 460500 | 3.366 |
| Mizrahi B [113] 2021 | Israel | Cohort study | 1352444 | BNT162b2 | ≥16 | SARS-CoV-2 infection | Delta (B.1.617.2) | After dose 2 (21-26) | 1736 | 475281 | 3.653 |
| Starrfelt J [109] 2022 | Norway | Cohort study | 128982 | Multiple vaccines | 18-44 | SARS-CoV-2 infection | Delta (B.1.617.2) | After dose 2 (2-10) | 3629 | 202928 | 17.88 |
| Starrfelt J [109] 2022 | Norway | Cohort study | 128982 | Multiple vaccines | 18-44 | SARS-CoV-2 infection | Delta (B.1.617.2) | After dose 2 (10-18) | 9623 | 133290 | 72.2 |
| Starrfelt J [109] 2022 | Norway | Cohort study | 128982 | Multiple vaccines | 18-44 | SARS-CoV-2 infection | Delta (B.1.617.2) | After dose 2 (18-26) | 2878 | 32526 | 88.48 |
| Starrfelt J [109] 2022 | Norway | Cohort study | 128982 | Multiple vaccines | 18-44 | SARS-CoV-2 infection | Delta (B.1.617.2) | After dose 2 (26-33) | 1434 | 11001 | 130.4 |
| Starrfelt J [109] 2022 | Norway | Cohort study | 128982 | Multiple vaccines | 18-44 | SARS-CoV-2 infection | Delta (B.1.617.2) | After dose 2 (≥ 33) | 828 | 4309 | 192.2 |
| Starrfelt J [109] 2022 | Norway | Cohort study | 165757 | Multiple vaccines | 45-64 | SARS-CoV-2 infection | Delta (B.1.617.2) | After dose 2 (2-10) | 1827 | 147044 | 12.42 |
| Starrfelt J [109] 2022 | Norway | Cohort study | 165757 | Multiple vaccines | 45-64 | SARS-CoV-2 infection | Delta (B.1.617.2) | After dose 2 (10-18) | 6897 | 151677 | 45.47 |
| Starrfelt J [109] 2022 | Norway | Cohort study | 165757 | Multiple vaccines | 45-64 | SARS-CoV-2 infection | Delta (B.1.617.2) | After dose 2 (18-26) | 4444 | 61704 | 72.02 |
| Starrfelt J [109] 2022 | Norway | Cohort study | 165757 | Multiple vaccines | 45-64 | SARS-CoV-2 infection | Delta (B.1.617.2) | After dose 2 (26-33) | 1454 | 13546 | 107.3 |
| Starrfelt J [109] 2022 | Norway | Cohort study | 165757 | Multiple vaccines | 45-64 | SARS-CoV-2 infection | Delta (B.1.617.2) | After dose 2 (≥ 33) | 514 | 4175 | 123.1 |
| Starrfelt J [109] 2022 | Norway | Cohort study | 165757 | Multiple vaccines | ≥65 | SARS-CoV-2 infection | Delta (B.1.617.2) | After dose 2 (2-10) | 72 | 27448 | 2.623 |
| Starrfelt J [109] 2022 | Norway | Cohort study | 165757 | Multiple vaccines | ≥65 | SARS-CoV-2 infection | Delta (B.1.617.2) | After dose 2 (10-18) | 669 | 104140 | 6.424 |
| Starrfelt J [109] 2022 | Norway | Cohort study | 165757 | Multiple vaccines | ≥65 | SARS-CoV-2 infection | Delta (B.1.617.2) | After dose 2 (18-26) | 2663 | 123884 | 21.5 |
| Starrfelt J [109] 2022 | Norway | Cohort study | 165757 | Multiple vaccines | ≥65 | SARS-CoV-2 infection | Delta (B.1.617.2) | After dose 2 (26-33) | 2345 | 56040 | 41.85 |
| Starrfelt J [109] 2022 | Norway | Cohort study | 165757 | Multiple vaccines | ≥65 | SARS-CoV-2 infection | Delta (B.1.617.2) | After dose 2 (≥ 33) | 832 | 15367 | 54.14 |
| Starrfelt J [109] 2022 | Norway | Cohort study | 128982 | BNT162b2 | ≥18 | SARS-CoV-2 infection | Delta (B.1.617.2) | After dose 2 (2-10) | 3352 | 231500 | 14.48 |
| Starrfelt J [109] 2022 | Norway | Cohort study | 128982 | BNT162b2 | ≥18 | SARS-CoV-2 infection | Delta (B.1.617.2) | After dose 2 (10-18) | 10998 | 278007 | 39.56 |
| Starrfelt J [109] 2022 | Norway | Cohort study | 128982 | BNT162b2 | ≥18 | SARS-CoV-2 infection | Delta (B.1.617.2) | After dose 2 (18-26) | 7509 | 176006 | 42.66 |
| Starrfelt J [109] 2022 | Norway | Cohort study | 128982 | BNT162b2 | ≥18 | SARS-CoV-2 infection | Delta (B.1.617.2) | After dose 2 (26-33) | 3429 | 67758 | 50.61 |
| Starrfelt J [109] 2022 | Norway | Cohort study | 128982 | BNT162b2 | ≥18 | SARS-CoV-2 infection | Delta (B.1.617.2) | After dose 2 (≥ 33) | 2100 | 23227 | 90.41 |
| Starrfelt J [109] 2022 | Norway | Cohort study | 128982 | mRNA-1273 | ≥18 | SARS-CoV-2 infection | Delta (B.1.617.2) | After dose 2 (2-10) | 753 | 46456 | 16.21 |
| Starrfelt J [109] 2022 | Norway | Cohort study | 128982 | mRNA-1273 | ≥18 | SARS-CoV-2 infection | Delta (B.1.617.2) | After dose 2 (10-18) | 2170 | 43097 | 50.35 |
| Starrfelt J [109] 2022 | Norway | Cohort study | 128982 | mRNA-1273 | ≥18 | SARS-CoV-2 infection | Delta (B.1.617.2) | After dose 2 (18-26) | 1193 | 21826 | 54.66 |
| Starrfelt J [109] 2022 | Norway | Cohort study | 128982 | mRNA-1273 | ≥18 | SARS-CoV-2 infection | Delta (B.1.617.2) | After dose 2 (26-33) | 835 | 7588 | 110 |
| Starrfelt J [109] 2022 | Norway | Cohort study | 128982 | mRNA-1273 | ≥18 | SARS-CoV-2 infection | Delta (B.1.617.2) | After dose 2 (≥ 33) | 72 | 603 | 119.4 |
| Starrfelt J [109] 2022 | Norway | Cohort study | 128982 | mRNA combined | ≥18 | SARS-CoV-2 infection | Delta (B.1.617.2) | After dose 2 (2-10) | 1386 | 95094 | 14.58 |
| Starrfelt J [109] 2022 | Norway | Cohort study | 128982 | mRNA combined | ≥18 | SARS-CoV-2 infection | Delta (B.1.617.2) | After dose 2 (10-18) | 3495 | 47688 | 73.29 |
| Starrfelt J [109] 2022 | Norway | Cohort study | 128982 | mRNA combined | ≥18 | SARS-CoV-2 infection | Delta (B.1.617.2) | After dose 2 (18-26) | 64 | 451 | 141.9 |
| Starrfelt J [109] 2022 | Norway | Cohort study | 128982 | mRNA combined | ≥18 | SARS-CoV-2 infection | Delta (B.1.617.2) | After dose 2 (26-33) | 3 | 36 | 83.33 |
| Starrfelt J [109] 2022 | Norway | Cohort study | 128982 | mRNA combined | ≥18 | SARS-CoV-2 infection | Delta (B.1.617.2) | After dose 2 (≥ 33) | 1 | 9 | 111.1 |
| Baden LR [51] 2021 | USA | RCT | 14746 | mRNA-1273 | 18-64 | Severe COVID-19 | Delta (B.1.617.2) | After dose 2 (47-56) | 7 | 1558 | 4.493 |
| Baden LR [51] 2021 | USA | RCT | 11431 | mRNA-1273 | ≥65 | Severe COVID-19 | Delta (B.1.617.2) | After dose 2 (47-56) | 6 | 544 | 11.03 |
| Baden LR [51] 2021 | USA | RCT | 14746 | mRNA-1273 | 18-64 | Severe COVID-19 | Delta (B.1.617.2) | After dose 2 (26-34) | 4 | 1289 | 3.103 |
| Baden LR [51] 2021 | USA | RCT | 11431 | mRNA-1273 | ≥65 | Severe COVID-19 | Delta (B.1.617.2) | After dose 2 (26-34) | 2 | 507 | 3.945 |
| Goldberg Y [106] 2021 | Israel | Cohort study | 936 711 | BNT162b2 | 16-39 | Severe COVID-19 | Delta (B.1.617.2) | After dose 2 (6–20) | 0 | 585762 | 0 |
| Goldberg Y [106] 2021 | Israel | Cohort study | 936 711 | BNT162b2 | 16-39 | Severe COVID-19 | Delta (B.1.617.2) | After dose 2 (20-24) | 0 | 901812 | 0 |
| Goldberg Y [106] 2021 | Israel | Cohort study | 936 711 | BNT162b2 | 16-39 | Severe COVID-19 | Delta (B.1.617.2) | After dose 2 (21-28) | 2 | 321938 | 0.006 |
| Goldberg Y [106] 2021 | Israel | Cohort study | 936 711 | BNT162b2 | 40-59 | Severe COVID-19 | Delta (B.1.617.2) | After dose 2 (6–20) | 1 | 290837 | 0.003 |
| Goldberg Y [106] 2021 | Israel | Cohort study | 936 711 | BNT162b2 | 40-59 | Severe COVID-19 | Delta (B.1.617.2) | After dose 2 (20-24) | 7 | 536102 | 0.013 |
| Goldberg Y [106] 2021 | Israel | Cohort study | 936 711 | BNT162b2 | 40-59 | Severe COVID-19 | Delta (B.1.617.2) | After dose 2 (21-28) | 19 | 662023 | 0.029 |
| Goldberg Y [106] 2021 | Israel | Cohort study | 936 711 | BNT162b2 | ≥60 | Severe COVID-19 | Delta (B.1.617.2) | After dose 2 (6–20) | 10 | 102428 | 0.098 |
| Goldberg Y [106] 2021 | Israel | Cohort study | 936 711 | BNT162b2 | ≥60 | Severe COVID-19 | Delta (B.1.617.2) | After dose 2 (20-24) | 26 | 128914 | 0.202 |
| Goldberg Y [106] 2021 | Israel | Cohort study | 936 711 | BNT162b2 | ≥60 | Severe COVID-19 | Delta (B.1.617.2) | After dose 2 (21-28) | 338 | 1065582 | 0.317 |
| Rosenberg ES [115] 2022 | USA | Cohort study | 6394228 | BNT162b2 | 18-49 | Severe COVID-19 | Delta (B.1.617.2) | After full vaccination (1-21) | 59 | 571755 | 0.103 |
| Rosenberg ES [115] 2022 | USA | Cohort study | 6394228 | BNT162b2 | 50-64 | Severe COVID-19 | Delta (B.1.617.2) | After full vaccination (1-21) | 144 | 559209 | 0.258 |
| Rosenberg ES [115] 2022 | USA | Cohort study | 6394228 | BNT162b2 | ≥65 | Severe COVID-19 | Delta (B.1.617.2) | After full vaccination (1-21) | 391 | 427979 | 0.914 |
| Rosenberg ES [115] 2022 | USA | Cohort study | 6394228 | mRNA­1273 | 18-49 | Severe COVID-19 | Delta (B.1.617.2) | After full vaccination (1-21) | 31 | 306215 | 0.101 |
| Rosenberg ES [115] 2022 | USA | Cohort study | 6394228 | mRNA­1273 | 50-64 | Severe COVID-19 | Delta (B.1.617.2) | After full vaccination (1-21) | 74 | 338352 | 0.219 |
| Rosenberg ES [115] 2022 | USA | Cohort study | 6394228 | mRNA­1273 | ≥65 | Severe COVID-19 | Delta (B.1.617.2) | After full vaccination (1-21) | 238 | 437431 | 0.544 |
| Rosenberg ES [115] 2022 | USA | Cohort study | 6394228 | Ad26.COV2.S | 18-49 | Severe COVID-19 | Delta (B.1.617.2) | After full vaccination (1-21) | 24 | 217706 | 0.11 |
| Rosenberg ES [115] 2022 | USA | Cohort study | 6394228 | Ad26.COV2.S | 50-64 | Severe COVID-19 | Delta (B.1.617.2) | After full vaccination (1-21) | 65 | 126627 | 0.513 |
| Rosenberg ES [115] 2022 | USA | Cohort study | 6394228 | Ad26.COV2.S | ≥65 | Severe COVID-19 | Delta (B.1.617.2) | After full vaccination (1-21) | 141 | 65182 | 2.163 |
| Rosenberg ES [115] 2022 | USA | Cohort study | 6394228 | BNT162b2 | 18-49 | Severe COVID-19 | Delta (B.1.617.2) | After full vaccination (4-26) | 12 | 151525 | 0.079 |
| Rosenberg ES [115] 2022 | USA | Cohort study | 6394228 | BNT162b2 | 50-64 | Severe COVID-19 | Delta (B.1.617.2) | After full vaccination (4-26) | 40 | 135138 | 0.296 |
| Rosenberg ES [115] 2022 | USA | Cohort study | 6394228 | BNT162b2 | ≥65 | Severe COVID-19 | Delta (B.1.617.2) | After full vaccination (4-26) | 329 | 343396 | 0.958 |
| Rosenberg ES [115] 2022 | USA | Cohort study | 6394228 | mRNA­1273 | 18-49 | Severe COVID-19 | Delta (B.1.617.2) | After full vaccination (4-26) | 15 | 174833 | 0.086 |
| Rosenberg ES [115] 2022 | USA | Cohort study | 6394228 | mRNA­1273 | 50-64 | Severe COVID-19 | Delta (B.1.617.2) | After full vaccination (4-26) | 10 | 120666 | 0.083 |
| Rosenberg ES [115] 2022 | USA | Cohort study | 6394228 | mRNA­1273 | ≥65 | Severe COVID-19 | Delta (B.1.617.2) | After full vaccination (4-26) | 216 | 426802 | 0.506 |
| Rosenberg ES [115] 2022 | USA | Cohort study | 6394228 | Ad26.COV2.S | 18-49 | Severe COVID-19 | Delta (B.1.617.2) | After full vaccination (4-26) | 14 | 50350 | 0.278 |
| Rosenberg ES [115] 2022 | USA | Cohort study | 6394228 | Ad26.COV2.S | 50-64 | Severe COVID-19 | Delta (B.1.617.2) | After full vaccination (4-26) | 27 | 54060 | 0.499 |
| Rosenberg ES [115] 2022 | USA | Cohort study | 6394228 | Ad26.COV2.S | ≥65 | Severe COVID-19 | Delta (B.1.617.2) | After full vaccination (4-26) | 87 | 49109 | 1.772 |
| Rosenberg ES [115] 2022 | USA | Cohort study | 6394228 | BNT162b2 | 18-49 | Severe COVID-19 | Delta (B.1.617.2) | After full vaccination (7-34) | 24 | 217519 | 0.11 |
| Rosenberg ES [115] 2022 | USA | Cohort study | 6394228 | BNT162b2 | 50-64 | Severe COVID-19 | Delta (B.1.617.2) | After full vaccination (7-34) | 43 | 131153 | 0.328 |
| Rosenberg ES [115] 2022 | USA | Cohort study | 6394228 | BNT162b2 | ≥65 | Severe COVID-19 | Delta (B.1.617.2) | After full vaccination (7-34) | 248 | 196823 | 1.26 |
| Rosenberg ES [115] 2022 | USA | Cohort study | 6394228 | mRNA­1273 | 18-49 | Severe COVID-19 | Delta (B.1.617.2) | After full vaccination (7-34) | 14 | 245546 | 0.057 |
| Rosenberg ES [115] 2022 | USA | Cohort study | 6394228 | mRNA­1273 | 50-64 | Severe COVID-19 | Delta (B.1.617.2) | After full vaccination (7-34) | 19 | 149357 | 0.127 |
| Rosenberg ES [115] 2022 | USA | Cohort study | 6394228 | mRNA­1273 | ≥65 | Severe COVID-19 | Delta (B.1.617.2) | After full vaccination (7-34) | 89 | 141769 | 0.628 |
| Starrfelt J [109] 2022 | Norway | Cohort study | 128982 | Multiple vaccines | ≥18 | Severe COVID-19 | Delta (B.1.617.2) | After dose 2 (2-10) | 13 | 378284 | 0.034 |
| Starrfelt J [109] 2022 | Norway | Cohort study | 128982 | Multiple vaccines | ≥18 | Severe COVID-19 | Delta (B.1.617.2) | After dose 2 (10-18) | 71 | 390477 | 0.182 |
| Starrfelt J [109] 2022 | Norway | Cohort study | 128982 | Multiple vaccines | ≥18 | Severe COVID-19 | Delta (B.1.617.2) | After dose 2 (18-26) | 207 | 219068 | 0.945 |
| Starrfelt J [109] 2022 | Norway | Cohort study | 128982 | Multiple vaccines | ≥18 | Severe COVID-19 | Delta (B.1.617.2) | After dose 2 (26-33) | 189 | 81074 | 2.331 |
| Starrfelt J [109] 2022 | Norway | Cohort study | 128982 | Multiple vaccines | ≥18 | Severe COVID-19 | Delta (B.1.617.2) | After dose 2 (≥ 33) | 143 | 24095 | 5.935 |
| Starrfelt J [109] 2022 | Norway | Cohort study | 165757 | Multiple vaccines | ≥18 | Severe COVID-19 | Delta (B.1.617.2) | After dose 2 (2-10) | 7 | 328570 | 0.021 |
| Starrfelt J [109] 2022 | Norway | Cohort study | 165757 | Multiple vaccines | ≥18 | Severe COVID-19 | Delta (B.1.617.2) | After dose 2 (10-18) | 25 | 295163 | 0.085 |
| Starrfelt J [109] 2022 | Norway | Cohort study | 165757 | Multiple vaccines | ≥18 | Severe COVID-19 | Delta (B.1.617.2) | After dose 2 (18-26) | 49 | 130431 | 0.376 |
| Starrfelt J [109] 2022 | Norway | Cohort study | 165757 | Multiple vaccines | ≥18 | Severe COVID-19 | Delta (B.1.617.2) | After dose 2 (26-33) | 37 | 45559 | 0.812 |
| Starrfelt J [109] 2022 | Norway | Cohort study | 165757 | Multiple vaccines | ≥18 | Severe COVID-19 | Delta (B.1.617.2) | After dose 2 (≥ 33) | 36 | 13892 | 2.591 |
| Starrfelt J [109] 2022 | Norway | Cohort study | 128982 | Multiple vaccines | 18-44 | Severe COVID-19 | Delta (B.1.617.2) | After dose 2 (2-10) | 6 | 203405 | 0.029 |
| Starrfelt J [109] 2022 | Norway | Cohort study | 128982 | Multiple vaccines | 18-44 | Severe COVID-19 | Delta (B.1.617.2) | After dose 2 (10-18) | 7 | 134065 | 0.052 |
| Starrfelt J [109] 2022 | Norway | Cohort study | 128982 | Multiple vaccines | 18-44 | Severe COVID-19 | Delta (B.1.617.2) | After dose 2 (18-26) | 13 | 32833 | 0.396 |
| Starrfelt J [109] 2022 | Norway | Cohort study | 128982 | Multiple vaccines | 18-44 | Severe COVID-19 | Delta (B.1.617.2) | After dose 2 (26-33) | 7 | 11146 | 0.628 |
| Starrfelt J [109] 2022 | Norway | Cohort study | 128982 | Multiple vaccines | 18-44 | Severe COVID-19 | Delta (B.1.617.2) | After dose 2 (≥ 33) | 3 | 4398 | 0.682 |
| Starrfelt J [109] 2022 | Norway | Cohort study | 165757 | Multiple vaccines | 45-64 | Severe COVID-19 | Delta (B.1.617.2) | After dose 2 (2-10) | 5 | 147227 | 0.034 |
| Starrfelt J [109] 2022 | Norway | Cohort study | 165757 | Multiple vaccines | 45-64 | Severe COVID-19 | Delta (B.1.617.2) | After dose 2 (10-18) | 35 | 152227 | 0.23 |
| Starrfelt J [109] 2022 | Norway | Cohort study | 165757 | Multiple vaccines | 45-64 | Severe COVID-19 | Delta (B.1.617.2) | After dose 2 (18-26) | 57 | 62141 | 0.917 |
| Starrfelt J [109] 2022 | Norway | Cohort study | 165757 | Multiple vaccines | 45-64 | Severe COVID-19 | Delta (B.1.617.2) | After dose 2 (26-33) | 14 | 13687 | 1.023 |
| Starrfelt J [109] 2022 | Norway | Cohort study | 165757 | Multiple vaccines | 45-64 | Severe COVID-19 | Delta (B.1.617.2) | After dose 2 (≥ 33) | 10 | 4232 | 2.363 |
| Starrfelt J [109] 2022 | Norway | Cohort study | 128982 | Multiple vaccines | ≥65 | Severe COVID-19 | Delta (B.1.617.2) | After dose 2 (2-10) | 2 | 27452 | 0.073 |
| Starrfelt J [109] 2022 | Norway | Cohort study | 128982 | Multiple vaccines | ≥65 | Severe COVID-19 | Delta (B.1.617.2) | After dose 2 (10-18) | 29 | 104185 | 0.278 |
| Starrfelt J [109] 2022 | Norway | Cohort study | 128982 | Multiple vaccines | ≥65 | Severe COVID-19 | Delta (B.1.617.2) | After dose 2 (18-26) | 137 | 124093 | 1.104 |
| Starrfelt J [109] 2022 | Norway | Cohort study | 128982 | Multiple vaccines | ≥65 | Severe COVID-19 | Delta (B.1.617.2) | After dose 2 (26-33) | 168 | 56240 | 2.987 |
| Starrfelt J [109] 2022 | Norway | Cohort study | 128982 | Multiple vaccines | ≥65 | Severe COVID-19 | Delta (B.1.617.2) | After dose 2 (≥ 33) | 130 | 15465 | 8.406 |
| Starrfelt J [109] 2022 | Norway | Cohort study | 128982 | BNT162b2 | ≥18 | Severe COVID-19 | Delta (B.1.617.2) | After dose 2 (2-10) | 13 | 231883 | 0.056 |
| Starrfelt J [109] 2022 | Norway | Cohort study | 128982 | BNT162b2 | ≥18 | Severe COVID-19 | Delta (B.1.617.2) | After dose 2 (10-18) | 62 | 278910 | 0.222 |
| Starrfelt J [109] 2022 | Norway | Cohort study | 128982 | BNT162b2 | ≥18 | Severe COVID-19 | Delta (B.1.617.2) | After dose 2 (18-26) | 189 | 176680 | 1.07 |
| Starrfelt J [109] 2022 | Norway | Cohort study | 128982 | BNT162b2 | ≥18 | Severe COVID-19 | Delta (B.1.617.2) | After dose 2 (26-33) | 172 | 68071 | 2.527 |
| Starrfelt J [109] 2022 | Norway | Cohort study | 128982 | BNT162b2 | ≥18 | Severe COVID-19 | Delta (B.1.617.2) | After dose 2 (≥ 33) | 141 | 23461 | 6.01 |
| Starrfelt J [109] 2022 | Norway | Cohort study | 128982 | mRNA-1273 | ≥18 | Severe COVID-19 | Delta (B.1.617.2) | After dose 2 (2-10) | 0 | 46540 | 0 |
| Starrfelt J [109] 2022 | Norway | Cohort study | 128982 | mRNA-1273 | ≥18 | Severe COVID-19 | Delta (B.1.617.2) | After dose 2 (10-18) | 4 | 43268 | 0.092 |
| Starrfelt J [109] 2022 | Norway | Cohort study | 128982 | mRNA-1273 | ≥18 | Severe COVID-19 | Delta (B.1.617.2) | After dose 2 (18-26) | 12 | 21962 | 0.546 |
| Starrfelt J [109] 2022 | Norway | Cohort study | 128982 | mRNA-1273 | ≥18 | Severe COVID-19 | Delta (B.1.617.2) | After dose 2 (26-33) | 15 | 7665 | 1.957 |
| Starrfelt J [109] 2022 | Norway | Cohort study | 128982 | mRNA-1273 | ≥18 | Severe COVID-19 | Delta (B.1.617.2) | After dose 2 (≥ 33) | 1 | 613 | 1.631 |
| Starrfelt J [109] 2022 | Norway | Cohort study | 128982 | mRNA combined | ≥18 | Severe COVID-19 | Delta (B.1.617.2) | After dose 2 (2-10) | 0 | 95288 | 0 |
| Starrfelt J [109] 2022 | Norway | Cohort study | 128982 | mRNA combined | ≥18 | Severe COVID-19 | Delta (B.1.617.2) | After dose 2 (10-18) | 4 | 47950 | 0.083 |
| Starrfelt J [109] 2022 | Norway | Cohort study | 128982 | mRNA combined | ≥18 | Severe COVID-19 | Delta (B.1.617.2) | After dose 2 (18-26) | 0 | 454 | 0 |
| Starrfelt J [109] 2022 | Norway | Cohort study | 128982 | mRNA combined | ≥18 | Severe COVID-19 | Delta (B.1.617.2) | After dose 2 (26-33) | 0 | 36 | 0 |
| Starrfelt J [109] 2022 | Norway | Cohort study | 128982 | mRNA combined | ≥18 | Severe COVID-19 | Delta (B.1.617.2) | After dose 2 (≥ 33) | 0 | 10 | 0 |
