# Supplementary material for: A comprehensive analysis of the efficacy and effectiveness of COVID-19 vaccines
Source: Front Immunol. 2022 Aug 26;13:945930. doi: 10.3389/fimmu.2022.945930 (PMC9459021; doi:10.3389/fimmu.2022.945930)
Supplement: Supplementary file 7 [file Table_6.docx]

**Supplementary Table 6** Duration of effectiveness of included studies after full vaccination during the SARS-CoV-2 Omicron variant period

| **First author/year** | **Type of study** | **Vaccine name** | **Age group (year)** | **Type of cases** | **Country** | **During variant Period/Variant** | **Time interval of after dose 2 (week)** | **Adjusted VE (95% CI)** | **Adjusted RR/OR (95% CI)** |
| --- | --- | --- | --- | --- | --- | --- | --- | --- | --- |
| Andrews N [97] 2022 | Case–control | BNT162b2 | ≥18 | Symptomatic COVID-19 | UK | Omicron (B.1.1.529) | 2-5 | 65.5 (63.9, 67.0) | 0.345 (0.330, 0.361) |
| Andrews N [97] 2022 | Case–control | BNT162b2 | ≥18 | Symptomatic COVID-19 | UK | Omicron (B.1.1.529) | 5-10 | 48.7 (47.1, 50.2) | 0.513 (0.498, 0.529) |
| Andrews N [97] 2022 | Case–control | BNT162b2 | ≥18 | Symptomatic COVID-19 | UK | Omicron (B.1.1.529) | 10-15 | 30.1 (28.7, 31.5) | 0.699 (0.685, 0.713) |
| Andrews N [97] 2022 | Case–control | BNT162b2 | ≥18 | Symptomatic COVID-19 | UK | Omicron (B.1.1.529) | 15-20 | 15.4 (14.2, 16.6) | 0.846 (0.836, 0.858) |
| Andrews N [97] 2022 | Case–control | BNT162b2 | ≥18 | Symptomatic COVID-19 | UK | Omicron (B.1.1.529) | 20-25 | 11.5 (10.1, 12.9) | 0.885 (0.871, 0.899) |
| Andrews N [97] 2022 | Case–control | BNT162b2 | ≥18 | Symptomatic COVID-19 | UK | Omicron (B.1.1.529) | ≥25 | 8.8 (7.0, 10.5) | 0.912 (0.895, 0.930) |
| Andrews N [97] 2022 | Case–control | ChAdOx1 nCoV-19 | ≥18 | Symptomatic COVID-19 | UK | Omicron (B.1.1.529) | 2-5 | 48.9 (39.2, 57.1) | 0.511 (0.429, 0.608) |
| Andrews N [97] 2022 | Case–control | ChAdOx1 nCoV-19 | ≥18 | Symptomatic COVID-19 | UK | Omicron (B.1.1.529) | 5-10 | 33.7 (25.0, 41.5) | 0.663 (0.585, 0.750) |
| Andrews N [97] 2022 | Case–control | ChAdOx1 nCoV-19 | ≥18 | Symptomatic COVID-19 | UK | Omicron (B.1.1.529) | 10-15 | 28.6 (20.9, 35.6) | 0.714 (0.644, 0.791) |
| Andrews N [97] 2022 | Case–control | ChAdOx1 nCoV-19 | ≥18 | Symptomatic COVID-19 | UK | Omicron (B.1.1.529) | 15-20 | 17.8 (13.4, 21.9) | 0.822 (0.781, 0.866) |
| Andrews N [97] 2022 | Case–control | ChAdOx1 nCoV-19 | ≥18 | Symptomatic COVID-19 | UK | Omicron (B.1.1.529) | 20-25 | 4.0 (1.9, 6.1) | 0.96 (0.939, 0.981) |
| Andrews N [97] 2022 | Case–control | ChAdOx1 nCoV-19 | ≥18 | Symptomatic COVID-19 | UK | Omicron (B.1.1.529) | ≥25 | −2.7 (−4.2, −1.2) | 1.027 (1.012, 1.042) |
| Andrews N [97] 2022 | Case–control | mRNA-1273 | ≥18 | Symptomatic COVID-19 | UK | Omicron (B.1.1.529) | 2-5 | 75.1 (70.8, 78.7) | 0.249 (0.213, 0.292) |
| Andrews N [97] 2022 | Case–control | mRNA-1273 | ≥18 | Symptomatic COVID-19 | UK | Omicron (B.1.1.529) | 5-10 | 52.8 (48.2, 57.1) | 0.472 (0.429, 0.518) |
| Andrews N [97] 2022 | Case–control | mRNA-1273 | ≥18 | Symptomatic COVID-19 | UK | Omicron (B.1.1.529) | 10-15 | 35.6 (32.7, 38.4) | 0.644 (0.616, 0.673) |
| Andrews N [97] 2022 | Case–control | mRNA-1273 | ≥18 | Symptomatic COVID-19 | UK | Omicron (B.1.1.529) | 15-20 | 25.3 (23.2, 27.4) | 0.747 (0.726, 0.768) |
| Andrews N [97] 2022 | Case–control | mRNA-1273 | ≥18 | Symptomatic COVID-19 | UK | Omicron (B.1.1.529) | 20-25 | 15.0 (11.6, 18.2) | 0.850 (0.818, 0.884) |
| Andrews N [97] 2022 | Case–control | mRNA-1273 | ≥18 | Symptomatic COVID-19 | UK | Omicron (B.1.1.529) | ≥25 | 14.9 (3.9, 24.7) | 0.851 (0.753, 0.961) |
| Baum U [123] 2022 | Cohort | BNT162b2 | ≥70 | Severe COVID-19 | Finland | Omicron (B.1.1.529) | 2-13 | 91 (79, 96) | 0.09 (0.04, 0.21) |
| Baum U [123] 2022 | Cohort | BNT162b2 | ≥70 | Severe COVID-19 | Finland | Omicron (B.1.1.529) | 13-26 | 76 (56, 86) | 0.24 (0.14, 0.44) |
| Baum U [123] 2022 | Cohort | BNT162b2 | ≥70 | Severe COVID-19 | Finland | Omicron (B.1.1.529) | ≥26 | 61 (48, 71) | 0.39 (0.29, 0.52) |
| Baum U [123] 2022 | Cohort | mRNA-1273 | ≥70 | Severe COVID-19 | Finland | Omicron (B.1.1.529) | 2-13 | 92 (43, 99) | 0.08 (0.01, 0.57) |
| Baum U [123] 2022 | Cohort | mRNA-1273 | ≥70 | Severe COVID-19 | Finland | Omicron (B.1.1.529) | 13-26 | 90 (28, 99) | 0.10 (0.01, 0.72) |
| Baum U [123] 2022 | Cohort | mRNA-1273 | ≥70 | Severe COVID-19 | Finland | Omicron (B.1.1.529) | ≥26 | 72 (43, 86) | 0.28 (0.14, 0.57) |
| Baum U [123] 2022 | Cohort | ChAdOx1 nCoV-19 | ≥70 | Severe COVID-19 | Finland | Omicron (B.1.1.529) | 2-13 | 99 (98, 99) | 0.01 (0.01, 0.02) |
| Baum U [123] 2022 | Cohort | ChAdOx1 nCoV-19 | ≥70 | Severe COVID-19 | Finland | Omicron (B.1.1.529) | 13-26 | 41 (-140, 86) | 0.59 (0.14, 2.14) |
| Baum U [123] 2022 | Cohort | ChAdOx1 nCoV-19 | ≥70 | Severe COVID-19 | Finland | Omicron (B.1.1.529) | ≥26 | 43 (-10, 70) | 0.57 (0.30, 1.10) |
| Buchan SA [120] 2022 | Case–control | BNT162b2 | 12-17 | Symptomatic COVID-19 | Canada | Omicron (B.1.1.529) | 1-8 | 51 (38, 61) | 0.49 (0.39, 0.62) |
| Buchan SA [120] 2022 | Case–control | BNT162b2 | 12-17 | Symptomatic COVID-19 | Canada | Omicron (B.1.1.529) | 8-17 | 31 (20, 41) | 0.69 (0.59, 0.80) |
| Buchan SA [120] 2022 | Case–control | BNT162b2 | 12-17 | Symptomatic COVID-19 | Canada | Omicron (B.1.1.529) | 17-26 | 29 (19, 38) | 0.71 (0.62, 0.81) |
| Buchan SA [120] 2022 | Case–control | BNT162b2 | 12-17 | Symptomatic COVID-19 | Canada | Omicron (B.1.1.529) | ≥26 | 29 (17, 38) | 0.71 (0.62, 0.83) |
| Buchan SA [120] 2022 | Case–control | BNT162b2 | 12-17 | Severe COVID-19 | Canada | Omicron (B.1.1.529) | 1-8 | 76 (-10, 95) | 0.24 (0.05, 1.10) |
| Buchan SA [120] 2022 | Case–control | BNT162b2 | 12-17 | Severe COVID-19 | Canada | Omicron (B.1.1.529) | 8-17 | 83 (55, 93) | 0.17 (0.07, 0.45) |
| Buchan SA [120] 2022 | Case–control | BNT162b2 | 12-17 | Severe COVID-19 | Canada | Omicron (B.1.1.529) | 17-26 | 82 (64, 91) | 0.18 (0.09, 0.36) |
| Buchan SA [120] 2022 | Case–control | BNT162b2 | 12-17 | Severe COVID-19 | Canada | Omicron (B.1.1.529) | ≥34 | 88 (77, 94) | 0.12 (0.06, 0.23) |
| Chemaitelly H [121] 2022 | Case–control | BNT162b2 | > 0 | Symptomatic COVID-19 | Qatar | Omicron (B.1.1.529) | 4-8 | 61.9 (49.9, 71.1) | 0.381 (0.289, 0.511) |
| Chemaitelly H [121] 2022 | Case–control | BNT162b2 | > 0 | Symptomatic COVID-19 | Qatar | Omicron (B.1.1.529) | 8-13 | 45.9 (33.8, 55.8) | 0.541 (0.442, 0.662) |
| Chemaitelly H [121] 2022 | Case–control | BNT162b2 | > 0 | Symptomatic COVID-19 | Qatar | Omicron (B.1.1.529) | 13-17 | 36.3 (25.1, 45.8) | 0.637 (0.542, 0.749) |
| Chemaitelly H [121] 2022 | Case–control | BNT162b2 | > 0 | Symptomatic COVID-19 | Qatar | Omicron (B.1.1.529) | 17-21 | 28.5 (18.0, 37.8) | 0.715 (0.622, 0.820) |
| Chemaitelly H [121] 2022 | Case–control | BNT162b2 | > 0 | Symptomatic COVID-19 | Qatar | Omicron (B.1.1.529) | 21-26 | 10.6 (−2.3, 21.9) | 0.894 (0.781, 1.023) |
| Chemaitelly H [121] 2022 | Case–control | BNT162b2 | > 0 | Symptomatic COVID-19 | Qatar | Omicron (B.1.1.529) | 26-30 | 14.3 (6.2, 21.8) | 0.857 (0.782, 0.938) |
| Chemaitelly H [121] 2022 | Case–control | BNT162b2 | > 0 | Symptomatic COVID-19 | Qatar | Omicron (B.1.1.529) | 30-34 | 9.6 (2.4, 16.3) | 0.904 (0.837, 0.976) |
| Chemaitelly H [121] 2022 | Case–control | BNT162b2 | > 0 | Symptomatic COVID-19 | Qatar | Omicron (B.1.1.529) | 34-38 | −7.5 (−15.3, −0.2) | 1.075 (1.002, 1.153) |
| Chemaitelly H [121] 2022 | Case–control | BNT162b2 | > 0 | Symptomatic COVID-19 | Qatar | Omicron (B.1.1.529) | 38-43 | 1.5 (-6.2, 8.7) | 0.985 (0.913, 1.062) |
| Chemaitelly H [121] 2022 | Case–control | BNT162b2 | > 0 | Symptomatic COVID-19 | Qatar | Omicron (B.1.1.529) | 43-47 | −17.7 (−25.6, −10.3) | 1.177 (1.103, 1.256) |
| Chemaitelly H [121] 2022 | Case–control | BNT162b2 | > 0 | Symptomatic COVID-19 | Qatar | Omicron (B.1.1.529) | 47-51 | −0.3 (−10.2, 8.6) | 1.003 (0.914, 1.102) |
| Chemaitelly H [121] 2022 | Case–control | BNT162b2 | > 0 | Symptomatic COVID-19 | Qatar | Omicron (B.1.1.529) | > 51 | 16.5 (3.1, 28.1) | 0.835 (0.719, 0.969) |
| Chemaitelly H [121] 2022 | Case–control | BNT162b2 | > 0 | Symptomatic COVID-19 | Qatar | Omicron (B.1.1.529) | ≥12 | 37.6 (28.8, 45.4) | 0.624 (0.546, 0.712) |
| Chemaitelly H [121] 2022 | Case–control | mRNA-1273 | > 0 | Symptomatic COVID-19 | Qatar | Omicron (B.1.1.529) | 4-17 | 44.8 (16.0, 63.8) | 0.552 (0.362, 0.840) |
| Chemaitelly H [121] 2022 | Case–control | mRNA-1273 | > 0 | Symptomatic COVID-19 | Qatar | Omicron (B.1.1.529) | 17-30 | 20.8 (13.7, 27.4) | 0.792 (0.726, 0.863) |
| Chemaitelly H [121] 2022 | Case–control | mRNA-1273 | > 0 | Symptomatic COVID-19 | Qatar | Omicron (B.1.1.529) | ≥30 | −9.3 (−16.3, −2.8) | 1.093 (1.028, 1.163) |
| Chemaitelly H [121] 2022 | Case–control | BNT162b2 | > 0 | Severe COVID-19 | Qatar | Omicron (B.1.1.529) | 4-30 | 73.7 (46.8, 87.0) | 0.263 (0.130, 0.532) |
| Chemaitelly H [121] 2022 | Case–control | BNT162b2 | > 0 | Severe COVID-19 | Qatar | Omicron (B.1.1.529) | ≥30 | 80.7 (71.3, 87.0) | 0.193 (0.130, 0.287) |
| Chemaitelly H [121] 2022 | Case–control | mRNA-1273 | > 0 | Severe COVID-19 | Qatar | Omicron (B.1.1.529) | 4-30 | 76.9 (19.2, 93.4) | 0.231 (0.066, 0.808) |
| Chemaitelly H [121] 2022 | Case–control | mRNA-1273 | > 0 | Severe COVID-19 | Qatar | Omicron (B.1.1.529) | ≥30 | 64.0 (39.1, 78.7) | 0.36 (0.213, 0.609) |
| Dorabawila V [68] 2022 | Cohort | BNT162b2 | 5-11 | SARS-CoV-2 infection | USA | Omicron (B.1.1.529) | 2-3 | 68.0 (63.0, 72.0) | 0.320 (0.280, 0.370) |
| Dorabawila V [68] 2022 | Cohort | BNT162b2 | 5-11 | SARS-CoV-2 infection | USA | Omicron (B.1.1.529) | 3-4 | 57.0 (55.0, 60.0) | 0.430 (0.400, 0.450) |
| Dorabawila V [68] 2022 | Cohort | BNT162b2 | 5-11 | SARS-CoV-2 infection | USA | Omicron (B.1.1.529) | 4-5 | 50.0 (48.0, 52.0) | 0.500 (0.480. 0.520) |
| Dorabawila V [68] 2022 | Cohort | BNT162b2 | 5-11 | SARS-CoV-2 infection | USA | Omicron (B.1.1.529) | 5-6 | 48.0 (47.0, 50.0) | 0.520 (0.500, 0.530) |
| Dorabawila V [68] 2022 | Cohort | BNT162b2 | 5-11 | SARS-CoV-2 infection | USA | Omicron (B.1.1.529) | 6-7 | 34.0 (31.0, 36.0) | 0.660 (0.640, 0.690) |
| Dorabawila V [68] 2022 | Cohort | BNT162b2 | 5-11 | SARS-CoV-2 infection | USA | Omicron (B.1.1.529) | 7-8 | 20.0 (16.0, 23.0) | 0.80 (0.77, 0.84) |
| Dorabawila V [68] 2022 | Cohort | BNT162b2 | 5-11 | SARS-CoV-2 infection | USA | Omicron (B.1.1.529) | 8-9 | 12.0 (6.0, 16.0) | 0.88 (0.84, 0.94) |
| Dorabawila V [68] 2022 | Cohort | BNT162b2 | 12-17 | SARS-CoV-2 infection | USA | Omicron (B.1.1.529) | 2-3 | 85.0 (84.0, 86.0) | 0.15 (0.14, 0.16) |
| Dorabawila V [68] 2022 | Cohort | BNT162b2 | 12-17 | SARS-CoV-2 infection | USA | Omicron (B.1.1.529) | 3-4 | 82.0 (81.0, 83.0) | 0.18 (0.17, 0.19) |
| Dorabawila V [68] 2022 | Cohort | BNT162b2 | 12-17 | SARS-CoV-2 infection | USA | Omicron (B.1.1.529) | 4-5 | 66.0 (64.0, 67.0) | 0.34 (0.33, 0.36) |
| Dorabawila V [68] 2022 | Cohort | BNT162b2 | 12-17 | SARS-CoV-2 infection | USA | Omicron (B.1.1.529) | 5-6 | 57.0 (56.0, 58.0) | 0.43 (0.42, 0.44) |
| Dorabawila V [68] 2022 | Cohort | BNT162b2 | 12-17 | SARS-CoV-2 infection | USA | Omicron (B.1.1.529) | 6-7 | 55.0 (54.0, 56.0) | 0.45 (0.44, 0.46) |
| Dorabawila V [68] 2022 | Cohort | BNT162b2 | 12-17 | SARS-CoV-2 infection | USA | Omicron (B.1.1.529) | 7-8 | 53.0 (52.0, 54.0) | 0.47 (0.46, 0.48) |
| Dorabawila V [68] 2022 | Cohort | BNT162b2 | 12-17 | SARS-CoV-2 infection | USA | Omicron (B.1.1.529) | 8-9 | 50.0 (48.0, 51.0) | 0.50 (0.49, 0.52) |
| Dorabawila V [68] 2022 | Cohort | BNT162b2 | 12-17 | SARS-CoV-2 infection | USA | Omicron (B.1.1.529) | 9-10 | 50.0 (48.0, 52.0) | 0.50 (0.48, 0.52) |
| Dorabawila V [68] 2022 | Cohort | BNT162b2 | 12-17 | SARS-CoV-2 infection | USA | Omicron (B.1.1.529) | 10-11 | 51.0 (48.0, 54.0) | 0.49 (0.46, 0.52) |
| Dorabawila V [68] 2022 | Cohort | BNT162b2 | 5-11 | Severe COVID-19 | USA | Omicron (B.1.1.529) | 2-3 | 100 (-189, 100) | 0.0 (0.0, 2.89) |
| Dorabawila V [68] 2022 | Cohort | BNT162b2 | 5-11 | Severe COVID-19 | USA | Omicron (B.1.1.529) | 3-4 | 73 (-7, 97) | 0.27 (0.03, 1.07) |
| Dorabawila V [68] 2022 | Cohort | BNT162b2 | 5-11 | Severe COVID-19 | USA | Omicron (B.1.1.529) | 4-5 | 82 (45, 96) | 0.18 (0.04, 0.55) |
| Dorabawila V [68] 2022 | Cohort | BNT162b2 | 5-11 | Severe COVID-19 | USA | Omicron (B.1.1.529) | 4-6 | 74 (36, 96) | 0.26 (0.04, 0.64) |
| Dorabawila V [68] 2022 | Cohort | BNT162b2 | 5-11 | Severe COVID-19 | USA | Omicron (B.1.1.529) | 6-7 | 68 (28, 91) | 0.32 (0.09, 0.72) |
| Dorabawila V [68] 2022 | Cohort | BNT162b2 | 5-11 | Severe COVID-19 | USA | Omicron (B.1.1.529) | 7-8 | 46 (-15, 77) | 0.54 (0.23, 1.15) |
| Dorabawila V [68] 2022 | Cohort | BNT162b2 | 5-11 | Severe COVID-19 | USA | Omicron (B.1.1.529) | 8-9 | 48 (-12, 75) | 0.52 (0.25, 1.12) |
| Dorabawila V [68] 2022 | Cohort | BNT162b2 | 12-17 | Severe COVID-19 | USA | Omicron (B.1.1.529) | 2-3 | 94 (76, 99) | 0.06 (0.01, 0.24) |
| Dorabawila V [68] 2022 | Cohort | BNT162b2 | 12-17 | Severe COVID-19 | USA | Omicron (B.1.1.529) | 3-4 | 95 (64, 100) | 0.05 (0.0, 0.36) |
| Dorabawila V [68] 2022 | Cohort | BNT162b2 | 12-17 | Severe COVID-19 | USA | Omicron (B.1.1.529) | 4-5 | 85 (63, 95) | 0.15 (0.05, 0.37) |
| Dorabawila V [68] 2022 | Cohort | BNT162b2 | 12-17 | Severe COVID-19 | USA | Omicron (B.1.1.529) | 4-6 | 78 (63, 88) | 0.22 (0.12, 0.37) |
| Dorabawila V [68] 2022 | Cohort | BNT162b2 | 12-17 | Severe COVID-19 | USA | Omicron (B.1.1.529) | 6-7 | 74 (61, 84) | 0.26 (0.39, 0.16) |
| Dorabawila V [68] 2022 | Cohort | BNT162b2 | 12-17 | Severe COVID-19 | USA | Omicron (B.1.1.529) | 7-8 | 74 (63, 82) | 0.26 (0.18, 0.37) |
| Dorabawila V [68] 2022 | Cohort | BNT162b2 | 12-17 | Severe COVID-19 | USA | Omicron (B.1.1.529) | 8-9 | 75 (64, 86) | 0.25 (0.14, 0.36) |
| Dorabawila V [68] 2022 | Cohort | BNT162b2 | 12-17 | Severe COVID-19 | USA | Omicron (B.1.1.529) | 9-10 | 75 (61, 83) | 0.25 (0.17, 0.39) |
| Dorabawila V [68] 2022 | Cohort | BNT162b2 | 12-17 | Severe COVID-19 | USA | Omicron (B.1.1.529) | 10-11 | 73 (53, 87) | 0.27 (0.13, 0.47) |
| Fowlkes AL [119] 2022 | Cohort | BNT162b2 | 12-15 | SARS-CoV-2 infection | USA | Omicron (B.1.1.529) | 2-21 | 59 (22, 79) | 0.41 (0.21, 0.78) |
| Fowlkes AL [119] 2022 | Cohort | BNT162b2 | 12-15 | SARS-CoV-2 infection | USA | Omicron (B.1.1.529) | > 21 | 62 (−28, 89) | 0.38 (0.11, 1.28) |
| Hansen CH [64] 2021 | Cohort | BNT162b2 | > 0 | SARS-CoV-2 infection | Danish | Omicron (B.1.1.529) | 2-6 | 55.2 (23.5, 73.7) | 0.448 (0.263, 0.765) |
| Hansen CH [64] 2021 | Cohort | BNT162b2 | > 0 | SARS-CoV-2 infection | Danish | Omicron (B.1.1.529) | 6-11 | 16.1 (−20.8, 41.7) | 0.839 (0.583, 1.208) |
| Hansen CH [64] 2021 | Cohort | BNT162b2 | > 0 | SARS-CoV-2 infection | Danish | Omicron (B.1.1.529) | 11-15 | 9.8 (−10.0, 26.1) | 0.902 (0.739, 1.10) |
| Hansen CH [64] 2021 | Cohort | BNT162b2 | > 0 | SARS-CoV-2 infection | Danish | Omicron (B.1.1.529) | 15-24 | −76.5 (−95.3, −59.5) | 1.765 (1.595, 1.953) |
| Hansen CH [64] 2021 | Cohort | mRNA-1273 | > 0 | SARS-CoV-2 infection | Danish | Omicron (B.1.1.529) | 2-6 | 36.7 (-69.9, 76.4) | 0.633 (0.236, 1.699) |
| Hansen CH [64] 2021 | Cohort | mRNA-1273 | > 0 | SARS-CoV-2 infection | Danish | Omicron (B.1.1.529) | 6-11 | 30.0 (-41.3, 65.4) | 0.700 (0.346, 1.413) |
| Hansen CH [64] 2021 | Cohort | mRNA-1273 | > 0 | SARS-CoV-2 infection | Danish | Omicron (B.1.1.529) | 11-15 | 4.2 (-30.8, 29.8) | 0.958 (0.702, 1.308) |
| Hansen CH [64] 2021 | Cohort | mRNA-1273 | > 0 | SARS-CoV-2 infection | Danish | Omicron (B.1.1.529) | 15-24 | −39.3 (−61.6, −20.0) | 1.393 (1.200, 1.616) |
| Powell AA [62] 2021 | Case–control | BNT162b2 | 16-17 | Symptomatic COVID-19 | UK | Omicron (B.1.1.529) | 2-5 | 71.3 (69.3, 73.1) | 0.287 (0.269, 0.307) |
| Powell AA [62] 2021 | Case–control | BNT162b2 | 16-17 | Symptomatic COVID-19 | UK | Omicron (B.1.1.529) | 5-10 | 49.5 (45.7, 53.0) | 0.505 (0.470, 0.543) |
| Powell AA [62] 2021 | Case–control | BNT162b2 | 16-17 | Symptomatic COVID-19 | UK | Omicron (B.1.1.529) | ≥10 | 22.6 (14.5, 29.9) | 0.774 (0.701, 0.855) |
| Ranzani OT [122] 2022 | Case–control | CoronaVac | ≥18 | Symptomatic COVID-19 | Brazil | Omicron (B.1.1.529) | 2-8 | 26.9 (25.1, 28.6) | 0.731 (0.714, 0.749) |
| Ranzani OT [122] 2022 | Case–control | CoronaVac | ≥18 | Symptomatic COVID-19 | Brazil | Omicron (B.1.1.529) | 8-26 | 5.0 (4.2, 5.9) | 0.950 (0.941, 0.958) |
| Ranzani OT [122] 2022 | Case–control | CoronaVac | ≥18 | Symptomatic COVID-19 | Brazil | Omicron (B.1.1.529) | ≥26 | 8.1 (7.0, 9.1) | 0.919 (0.909, 0.93) |
| Ranzani OT [122] 2022 | Case–control | CoronaVac | 18-60 | Symptomatic COVID-19 | Brazil | Omicron (B.1.1.529) | ≥26 | 3.7 (2.5, 5.0) | 0.963 (0.950, 0.975) |
| Ranzani OT [122] 2022 | Case–control | CoronaVac | 60-74 | Symptomatic COVID-19 | Brazil | Omicron (B.1.1.529) | ≥26 | 21.8 (19.3, 24.2) | 0.782 (0.758, 0.817) |
| Ranzani OT [122] 2022 | Case–control | CoronaVac | ≥ 75 | Symptomatic COVID-19 | Brazil | Omicron (B.1.1.529) | ≥26 | 27.2 (23.5, 30.7) | 0.728 (0.693, 0.765) |
| Ranzani OT [122] 2022 | Case–control | CoronaVac | 18-60 | Severe COVID-19 | Brazil | Omicron (B.1.1.529) | ≥26 | 68.8 (63.2, 73.6) | 0.312 (0.264, 0.368) |
| Ranzani OT [122] 2022 | Case–control | CoronaVac | 60-74 | Severe COVID-19 | Brazil | Omicron (B.1.1.529) | ≥26 | 58.9 (52.9, 64.1) | 0.411 (0.471, 0.359) |
| Ranzani OT [122] 2022 | Case–control | CoronaVac | ≥ 75 | Severe COVID-19 | Brazil | Omicron (B.1.1.529) | ≥26 | 46.2 (39.4, 52.2) | 0.538 (0.478, 0.606) |
| Ranzani OT [122] 2022 | Case–control | CoronaVac | ≥18 | Severe COVID-19 | Brazil | Omicron (B.1.1.529) | 2-8 | 49.9 (30.7, 63.7) | 0.501 (0.363, 0.693) |
| Ranzani OT [122] 2022 | Case–control | CoronaVac | ≥18 | Severe COVID-19 | Brazil | Omicron (B.1.1.529) | 8-26 | 62.6 (58.5, 66.3) | 0.374 (0.337, 0.415) |
| Ranzani OT [122] 2022 | Case–control | CoronaVac | ≥18 | Severe COVID-19 | Brazil | Omicron (B.1.1.529) | ≥26 | 57.0 (53.5, 60.2) | 0.43 (0.398, 0.465) |
| Šmíd M [63] 2022 | Cohort | BNT162b2 | > 0 | Severe COVID-19 | Czech | Omicron (B.1.1.529) | 2-11 | 45 (29, 57) | 0.55 (0.43, 0.71) |
| Šmíd M [63] 2022 | Cohort | BNT162b2 | > 0 | Severe COVID-19 | Czech | Omicron (B.1.1.529) | ≥11 | 29 (21, 37) | 0.71 (0.63, 0.79) |
| Tartof SY [124] 2022 | Case–control | BNT162b2 | ≥18 | Severe COVID-19 | USA | Omicron (B.1.1.529) | 2-14 | 68 (48, 80) | 0.32 (0.20, 0.52) |
| Tartof SY [124] 2022 | Case–control | BNT162b2 | ≥18 | Severe COVID-19 | USA | Omicron (B.1.1.529) | 14-27 | 68 (53, 78) | 0.32 (0.22, 0.47) |
| Tartof SY [124] 2022 | Case–control | BNT162b2 | ≥18 | Severe COVID-19 | USA | Omicron (B.1.1.529) | 27-40 | 72 (63, 79) | 0.28 (0.21, 0.37) |
| Tartof SY [124] 2022 | Case–control | BNT162b2 | ≥18 | Severe COVID-19 | USA | Omicron (B.1.1.529) | ≥40 | 41 (21, 55) | 0.59 (0.45, 0.79) |
| Tartof SY [124] 2022 | Case–control | BNT162b2 | 18-64 | Severe COVID-19 | USA | Omicron (B.1.1.529) | 2-14 | 60 (27, 78) | 0.40 (0.22, 0.73) |
| Tartof SY [124] 2022 | Case–control | BNT162b2 | 18-64 | Severe COVID-19 | USA | Omicron (B.1.1.529) | 14-27 | 62 (40, 76) | 0.38 (0.24, 0.60) |
| Tartof SY [124] 2022 | Case–control | BNT162b2 | 18-64 | Severe COVID-19 | USA | Omicron (B.1.1.529) | 27-40 | 69 (55, 79) | 0.31 (0.21, 0.45) |
| Tartof SY [124] 2022 | Case–control | BNT162b2 | 18-64 | Severe COVID-19 | USA | Omicron (B.1.1.529) | ≥40 | 27 (-25, 58) | 0.73 (0.42, 1.25) |
| Tartof SY [124] 2022 | Case–control | BNT162b2 | ≥ 65 | Severe COVID-19 | USA | Omicron (B.1.1.529) | 2-14 | 81 (53, 92) | 0.19 (0.08, 0.47) |
| Tartof SY [124] 2022 | Case–control | BNT162b2 | ≥ 65 | Severe COVID-19 | USA | Omicron (B.1.1.529) | 14-27 | 76 (54, 88) | 0.24 (0.12, 0.46) |
| Tartof SY [124] 2022 | Case–control | BNT162b2 | ≥ 65 | Severe COVID-19 | USA | Omicron (B.1.1.529) | 27-40 | 76 (62, 85) | 0.24 (0.15, 0.38) |
| Tartof SY [124] 2022 | Case–control | BNT162b2 | ≥ 65 | Severe COVID-19 | USA | Omicron (B.1.1.529) | ≥40 | 50 (30, 64) | 0.50 (0.36, 0.70) |
| Tseng HF [88] 2022 | Case–control | mRNA-1273 | ≥ 18 | SARS-CoV-2 infection | USA | Omicron (B.1.1.529) | 2-12 | 44.0 (35.1, 51.6) | 0.56 (0.484, 0.649) |
| Tseng HF [88] 2022 | Case–control | mRNA-1273 | ≥ 18 | SARS-CoV-2 infection | USA | Omicron (B.1.1.529) | 12-26 | 23.5 (16.4, 30.0) | 0.765 (0.700, 0.836) |
| Tseng HF [88] 2022 | Case–control | mRNA-1273 | ≥ 18 | SARS-CoV-2 infection | USA | Omicron (B.1.1.529) | 26-39 | 13.8 (10.2, 17.3) | 0.862 (0.827, 0.898) |
| Tseng HF [88] 2022 | Case–control | mRNA-1273 | ≥ 18 | SARS-CoV-2 infection | USA | Omicron (B.1.1.529) | ≥39 | 5.9 (0.4, 11.0) | 0.941 (0.890, 0.996) |
| Veneti L [100] 2022 | Cohort | BNT162b2 | 16-17 | SARS-CoV-2 infection | Norway | Omicron (B.1.1.529) | 1-5 | 53·1 (42·6, 61·7) | 0.469 (0.383, 0.574) |
| Veneti L [100] 2022 | Cohort | BNT162b2 | 16-17 | SARS-CoV-2 infection | Norway | Omicron (B.1.1.529) | 5-9 | 45·7 (34·8, 54·7) | 0.443 (0.453, 0.652) |
| Veneti L [100] 2022 | Cohort | BNT162b2 | 16-17 | SARS-CoV-2 infection | Norway | Omicron (B.1.1.529) | ≥9 | 23·3 (2·7, 39·5) | 0.667 (0.605, 0.973) |
