# Supplementary material for: A comprehensive analysis of the efficacy and effectiveness of COVID-19 vaccines
Source: Front Immunol. 2022 Aug 26;13:945930. doi: 10.3389/fimmu.2022.945930 (PMC9459021; doi:10.3389/fimmu.2022.945930)
Supplement: Supplementary file 8 [file Table_7.docx]

**Supplementary Table 7** Duration of effectiveness of included studies for the COVID-19 vaccine booster immunization compared between booster vaccinees and unvaccinated group during the Omicron variant period

| **First author/year** | **Type of study** | **Vaccine name** | **Age group (Year)** | **Type of cases** | **Country** | **During variant Period/Variant** | **Time interval of since final dose, weeks** | **Adjusted VE (95% CI)** | **Adjusted RR/OR (95% CI)** |
| --- | --- | --- | --- | --- | --- | --- | --- | --- | --- |
| Andrews N [97] 2022 | Case–control | ChAdOx1 nCoV-19/ChAdOx1 nCoV-19/BNT162b2 | ≥18 | Symptomatic COVID-19 | UK | Omicron (B.1.1.529) | 1-2 | 58.8 (57.8, 59.7) | 0.412 (0.403, 0.422) |
| Andrews N [97] 2022 | Case–control | ChAdOx1 nCoV-19/ChAdOx1 nCoV-19/BNT162b2 | ≥18 | Symptomatic COVID-19 | UK | Omicron (B.1.1.529) | 2-5 | 62.4 (61.8, 63.0) | 0.376 (0.37, 0.382) |
| Andrews N [97] 2022 | Case–control | ChAdOx1 nCoV-19/ChAdOx1 nCoV-19/BNT162b2 | ≥18 | Symptomatic COVID-19 | UK | Omicron (B.1.1.529) | 5-10 | 52.9 (52.1, 53.7) | 0.471 (0.463, 0.479) |
| Andrews N [97] 2022 | Case–control | ChAdOx1 nCoV-19/ChAdOx1 nCoV-19/BNT162b2 | ≥18 | Symptomatic COVID-19 | UK | Omicron (B.1.1.529) | ≥10 | 39.6 (38.0, 41.1) | 0.604 (0.589, 0.620) |
| Andrews N [97] 2022 | Case–control | ChAdOx1 nCoV-19/ChAdOx1 nCoV-19/mRNA-1273 | ≥18 | Symptomatic COVID-19 | UK | Omicron (B.1.1.529) | 1-2 | 68.0 (67.0, 68.9) | 0.32 (0.311, 0.33) |
| Andrews N [97] 2022 | Case–control | ChAdOx1 nCoV-19/ChAdOx1 nCoV-19/mRNA-1273 | ≥18 | Symptomatic COVID-19 | UK | Omicron (B.1.1.529) | 2-5 | 70.1 (69.5, 70.7) | 0.299 (0.293, 0.305) |
| Andrews N [97] 2022 | Case–control | ChAdOx1 nCoV-19/ChAdOx1 nCoV-19/mRNA-1273 | ≥18 | Symptomatic COVID-19 | UK | Omicron (B.1.1.529) | 5-10 | 60.9 (59.7, 62.1) | 0.391 (0.379, 0.403) |
| Andrews N [97] 2022 | Case–control | ChAdOx1 nCoV-19/ChAdOx1 nCoV-19/ChAdOx1 nCoV-19 | ≥18 | Symptomatic COVID-19 | UK | Omicron (B.1.1.529) | 1-2 | 57.7 (37.6, 71.3) | 0.423 (0.287, 0.624) |
| Andrews N [97] 2022 | Case–control | ChAdOx1 nCoV-19/ChAdOx1 nCoV-19/ChAdOx1 nCoV-19 | ≥18 | Symptomatic COVID-19 | UK | Omicron (B.1.1.529) | 2-5 | 55.6 (44.4, 64.6) | 0.444 (0.354, 0.556) |
| Andrews N [97] 2022 | Case–control | ChAdOx1 nCoV-19/ChAdOx1 nCoV-19/ChAdOx1 nCoV-19 | ≥18 | Symptomatic COVID-19 | UK | Omicron (B.1.1.529) | 5-10 | 46.7 (34.3, 56.7) | 0.533 (0.433, 0.657) |
| Andrews N [97] 2022 | Case–control | BNT162b2/BNT162b2/BNT162b2 | ≥18 | Symptomatic COVID-19 | UK | Omicron (B.1.1.529) | 1-2 | 66.9 (66.1, 67.6) | 0.331 (0.324, 0.339) |
| Andrews N [97] 2022 | Case–control | BNT162b2/BNT162b2/BNT162b2 | ≥18 | Symptomatic COVID-19 | UK | Omicron (B.1.1.529) | 2-5 | 67.2 (66.5, 67.8) | 0.328 (0.322, 0.335) |
| Andrews N [97] 2022 | Case–control | BNT162b2/BNT162b2/BNT162b2 | ≥18 | Symptomatic COVID-19 | UK | Omicron (B.1.1.529) | 5-10 | 55.0 (54.2, 55.8) | 0.45 (0.442, 0.458) |
| Andrews N [97] 2022 | Case–control | BNT162b2/BNT162b2/BNT162b2 | ≥18 | Symptomatic COVID-19 | UK | Omicron (B.1.1.529) | ≥10 | 45.7 (44.7, 46.7) | 0.543 (0.533, 0.553) |
| Andrews N [97] 2022 | Case–control | BNT162b2/BNT162b2/BNT162b2/mRNA-1273 | ≥18 | Symptomatic COVID-19 | UK | Omicron (B.1.1.529) | 1-2 | 74.0 (73.1, 74.9) | 0.26 (0.251, 0.269) |
| Andrews N [97] 2022 | Case–control | BNT162b2/BNT162b2/BNT162b2/mRNA-1273 | ≥18 | Symptomatic COVID-19 | UK | Omicron (B.1.1.529) | 2-5 | 73.9 (73.1, 74.6) | 0.261 (0.254, 0.269) |
| Andrews N [97] 2022 | Case–control | BNT162b2/BNT162b2/BNT162b2/mRNA-1273 | ≥18 | Symptomatic COVID-19 | UK | Omicron (B.1.1.529) | 5-10 | 64.4 (62.6, 66.1) | 0.356 (0.339, 0.374) |
| Andrews N [97] 2022 | Case–control | mRNA-1273/mRNA-1273/BNT162b2 | ≥18 | Symptomatic COVID-19 | UK | Omicron (B.1.1.529) | 1-2 | 64.3 (61.7, 66.8) | 0.357 (0.332, 0.383) |
| Andrews N [97] 2022 | Case–control | mRNA-1273/mRNA-1273/BNT162b2 | ≥18 | Symptomatic COVID-19 | UK | Omicron (B.1.1.529) | 2-5 | 64.9 (62.3, 67.3) | 0.351 (0.327, 0.377) |
| Andrews N [97] 2022 | Case–control | mRNA-1273/mRNA-1273/mRNA-1273 | ≥18 | Symptomatic COVID-19 | UK | Omicron (B.1.1.529) | 1-2 | 68.1 (65.6, 70.5) | 0.319 (0.295, 0.344) |
| Andrews N [97] 2022 | Case–control | mRNA-1273/mRNA-1273/mRNA-1273 | ≥18 | Symptomatic COVID-19 | UK | Omicron (B.1.1.529) | 2-5 | 66.3 (63.7, 68.8) | 0.337 (0.363, 0.312) |
| Baum U [123] 2022 | Cohort study | BNT162b2/BNT162b2/BNT162b2 | ≥70 | Severe COVID-19 | Finland | Omicron (B.1.1.529) | 2-8 | 95 (94, 97) | 0.05 (0.03, 0.06) |
| Baum U [123] 2022 | Cohort study | BNT162b2/BNT162b2/BNT162b2 | ≥70 | Severe COVID-19 | Finland | Omicron (B.1.1.529) | > 8 | 90 (87, 93) | 0.10 (0.07, 0.13) |
| Baum U [123] 2022 | Cohort study | BNT162b2/BNT162b2/BNT162b2/mRNA-1273 | ≥70 | Severe COVID-19 | Finland | Omicron (B.1.1.529) | 2-8 | 94 (89, 97) | 0.06 (0.03, 0.11) |
| Baum U [123] 2022 | Cohort study | BNT162b2/BNT162b2/BNT162b2/mRNA-1273 | ≥70 | Severe COVID-19 | Finland | Omicron (B.1.1.529) | > 8 | 48 (-13, 76) | 0.52 (0.24,1.13) |
| Baum U [123] 2022 | Cohort study | mRNA-1273/mRNA-1273/BNT162b2 | ≥70 | Severe COVID-19 | Finland | Omicron (B.1.1.529) | 2-8 | 96 (82, 99) | 0.04 (0.01, 0.18) |
| Baum U [123] 2022 | Cohort study | mRNA-1273/mRNA-1273/BNT162b2 | ≥70 | Severe COVID-19 | Finland | Omicron (B.1.1.529) | > 8 | 100 | 0 |
| Baum U [123] 2022 | Cohort study | mRNA-1273/mRNA-1273/mRNA-1273 | ≥70 | Severe COVID-19 | Finland | Omicron (B.1.1.529) | 2-8 | 97 (92, 99) | 0.03 (0.01, 0.08) |
| Baum U [123] 2022 | Cohort study | mRNA-1273/mRNA-1273/mRNA-1273 | ≥70 | Severe COVID-19 | Finland | Omicron (B.1.1.529) | > 8 | 92 (79, 97) | 0.08 (0.03, 0.21) |
| Baum U [123] 2022 | Cohort study | ChAdOx1 nCoV-19/ChAdOx1 nCoV-19/BNT162b2 | ≥70 | Severe COVID-19 | Finland | Omicron (B.1.1.529) | 2-8 | 98 (89, 100) | 0.02 (0.0, 0.11) |
| Baum U [123] 2022 | Cohort study | ChAdOx1 nCoV-19/ChAdOx1 nCoV-19/BNT162b2 | ≥70 | Severe COVID-19 | Finland | Omicron (B.1.1.529) | > 8 | 90 (27, 99) | 0.10 (0.01, 0.73) |
| Baum U [123] 2022 | Cohort study | ChAdOx1 nCoV-19/ChAdOx1 nCoV-19/mRNA-1273 | ≥70 | Severe COVID-19 | Finland | Omicron (B.1.1.529) | 2-8 | 100 | 0 |
| Baum U [123] 2022 | Cohort study | ChAdOx1 nCoV-19/ChAdOx1 nCoV-19/mRNA-1273 | ≥70 | Severe COVID-19 | Finland | Omicron (B.1.1.529) | > 8 | 40 (-336, 92) | 0.60 (0.08, 4.36) |
| Chemaitelly H [121] 2022 | Case–control | BNT162b2/BNT162b2/BNT162b2 | > 0 | Symptomatic COVID-19 | Qatar | Omicron (B.1.1.529) | 1-2 | 15.8 (0.9, 28.4) | 0.842 (0.716, 0.991) |
| Chemaitelly H [121] 2022 | Case–control | BNT162b2/BNT162b2/BNT162b2 | > 0 | Symptomatic COVID-19 | Qatar | Omicron (B.1.1.529) | 2-4 | 53.6 (47.4, 59.1) | 0.464 (0.409, 0.526) |
| Chemaitelly H [121] 2022 | Case–control | BNT162b2/BNT162b2/BNT162b2 | > 0 | Symptomatic COVID-19 | Qatar | Omicron (B.1.1.529) | 4-6 | 56.6 (50.8, 61.7) | 0.434 (0.383, 0.492) |
| Chemaitelly H [121] 2022 | Case–control | BNT162b2/BNT162b2/BNT162b2 | > 0 | Symptomatic COVID-19 | Qatar | Omicron (B.1.1.529) | 6-8 | 46.2 (39.7, 52.0) | 0.538 (0.48, 0.603) |
| Chemaitelly H [121] 2022 | Case–control | BNT162b2/BNT162b2/BNT162b2 | > 0 | Symptomatic COVID-19 | Qatar | Omicron (B.1.1.529) | 8-10 | 38.0 (28.1, 46.5) | 0.62 (0.535, 0.719) |
| Chemaitelly H [121] 2022 | Case–control | BNT162b2/BNT162b2/BNT162b2 | > 0 | Symptomatic COVID-19 | Qatar | Omicron (B.1.1.529) | 10-12 | 43.7 (32.9, 52.7) | 0.563 (0.473, 0.671) |
| Chemaitelly H [121] 2022 | Case–control | mRNA-1273/mRNA-1273/mRNA-1273 | > 0 | Symptomatic COVID-19 | Qatar | Omicron (B.1.1.529) | 1-2 | 3.6 (-31.0, 29.1) | 0.964 (0.709, 1.31) |
| Chemaitelly H [121] 2022 | Case–control | mRNA-1273/mRNA-1273/mRNA-1273 | > 0 | Symptomatic COVID-19 | Qatar | Omicron (B.1.1.529) | 2-4 | 53.1 (40.7, 62.8) | 0.469 (0.372, 0.593) |
| Chemaitelly H [121] 2022 | Case–control | mRNA-1273/mRNA-1273/mRNA-1273 | > 0 | Symptomatic COVID-19 | Qatar | Omicron (B.1.1.529) | 4-6 | 54.6 (41.1, 65.0) | 0.454 (0.35, 0.589) |
| Chemaitelly H [121] 2022 | Case–control | mRNA-1273/mRNA-1273/mRNA-1273 | > 0 | Symptomatic COVID-19 | Qatar | Omicron (B.1.1.529) | 6-8 | 38.6 (19.4, 53.1) | 0.614 (0.469, 0.806) |
| Chemaitelly H [121] 2022 | Case–control | BNT162b2/BNT162b2/BNT162b2 | > 0 | Severe COVID-19 | Qatar | Omicron (B.1.1.529) | 1-6 | 90.6 (77.8, 96.0) | 0.094 (0.04,0.222) |
| Chemaitelly H [121] 2022 | Case–control | BNT162b2/BNT162b2/BNT162b2 | > 0 | Severe COVID-19 | Qatar | Omicron (B.1.1.529) | ≥6 | 90.8 (81.5, 95.5) | 0.092 (0.045, 0.185) |
| Chemaitelly H [121] 2022 | Case–control | mRNA-1273/mRNA-1273/mRNA-1273 | > 0 | Severe COVID-19 | Qatar | Omicron (B.1.1.529) | 1-6 | 80.8 (-51.9, 97.6) | 0.192 (0.024, 1.519) |
| Chemaitelly H [121] 2022 | Case–control | mRNA-1273/mRNA-1273/mRNA-1273 | > 0 | Severe COVID-19 | Qatar | Omicron (B.1.1.529) | ≥6 | 100 | 0 |
| Ferdinands JM [85] 2022 | Cohort study | BNT162b2 or mRNA-1273 | ≥ 18 | Severe COVID-19 | USA | Omicron (B.1.1.529) | 2-8 | 91 (88, 93) | 0.09 (0.07, 0.12) |
| Ferdinands JM [85] 2022 | Cohort study | BNT162b2 or mRNA-1273 | ≥ 18 | Severe COVID-19 | USA | Omicron (B.1.1.529) | 8-17 | 88 (85, 90) | 0.12 (0.10, 0.15) |
| Ferdinands JM [85] 2022 | Cohort study | BNT162b2 or mRNA-1273 | ≥ 18 | Severe COVID-19 | USA | Omicron (B.1.1.529) | ≥17 | 78 (67, 85) | 0.22 (0.15, 0.33) |
| Kirsebom FCM [111] 2022 | Case–control | BNT162b2 or mRNA-1273 | ≥18 | Symptomatic COVID-19 | UK | Omicron (B.1.1.529) | 2-5 | 70.2 (69.5, 71.0) | 0.298 (0.29, 0.305) |
| Kirsebom FCM [111] 2022 | Case–control | BNT162b2 or mRNA-1273 | ≥18 | Symptomatic COVID-19 | UK | Omicron (B.1.1.529) | 5-10 | 66.2 (65.5, 66.9) | 0.338 (0.331, 0.345) |
| Kirsebom FCM [111] 2022 | Case–control | BNT162b2 or mRNA-1273 | ≥18 | Symptomatic COVID-19 | UK | Omicron (B.1.1.529) | 10-15 | 54.3 (53.1, 55.5) | 0.457 (0.445, 0.469) |
| Kirsebom FCM [111] 2022 | Case–control | BNT162b2 or mRNA-1273 | ≥18 | Symptomatic COVID-19 | UK | Omicron (B.1.1.529) | ≥15 | 45.5 (43.8, 47.2) | 0.545 (0.528, 0.562) |
| Kirsebom FCM [111] 2022 | Case–control | BNT162b2 or mRNA-1273 | ≥18 | Symptomatic COVID-19 | UK | Omicron BA.2 | 2-5 | 74.2 (72.4, 75.8) | 0.258 (0.242, 0.276) |
| Kirsebom FCM [111] 2022 | Case–control | BNT162b2 or mRNA-1273 | ≥18 | Symptomatic COVID-19 | UK | Omicron BA.2 | 5-10 | 68.1 (66.7, 69.5) | 0.319 (0.305, 0.333) |
| Kirsebom FCM [111] 2022 | Case–control | BNT162b2 or mRNA-1273 | ≥18 | Symptomatic COVID-19 | UK | Omicron BA.2 | 10-15 | 58.6 (56.4, 60.7) | 0.414 (0.393, 0.436) |
| Kirsebom FCM [111] 2022 | Case–control | BNT162b2 or mRNA-1273 | ≥18 | Symptomatic COVID-19 | UK | Omicron BA.2 | ≥15 | 48.4 (45.2, 51.4) | 0.516 (0.486, 0.548) |
| Ranzani OT [122] 2022 | Case–control | CoronaVac/CoronaVac/CoronaVac | ≥18 | Symptomatic COVID-19 | Brazil | Omicron (B.1.1.529) | 1-8 | 15.0 (12.0, 18.0) | 0.85 (0.82, 0.88) |
| Ranzani OT [122] 2022 | Case–control | CoronaVac/CoronaVac/CoronaVac | ≥18 | Symptomatic COVID-19 | Brazil | Omicron (B.1.1.529) | >8 | 0.4 (-2.2, 2.9) | 0.996 (0.971, 1.022) |
| Ranzani OT [122] 2022 | Case–control | CoronaVac/CoronaVac/BNT162b2 | ≥18 | Symptomatic COVID-19 | Brazil | Omicron (B.1.1.529) | 1-8 | 56.8 (56.3, 57.4) | 0.432 (0.426, 0.437) |
| Ranzani OT [122] 2022 | Case–control | CoronaVac/CoronaVac/BNT162b2 | ≥18 | Symptomatic COVID-19 | Brazil | Omicron (B.1.1.529) | >8 | 34.9 (34.3, 35.6) | 0.651 (0.644, 0.657) |
| Ranzani OT [122] 2022 | Case–control | CoronaVac/CoronaVac/CoronaVac | 18-60 | Symptomatic COVID-19 | Brazil | Omicron (B.1.1.529) | 1-8 | 13.8 (10.3, 17.1) | 0.862 (0.829, 0.897) |
| Ranzani OT [122] 2022 | Case–control | CoronaVac/CoronaVac/CoronaVac | 18-60 | Symptomatic COVID-19 | Brazil | Omicron (B.1.1.529) | >8 | −10.4 (−15.3, −5.2) | 1.104 (1.052, 1.153) |
| Ranzani OT [122] 2022 | Case–control | CoronaVac/CoronaVac/BNT162b2 | 18-60 | Symptomatic COVID-19 | Brazil | Omicron (B.1.1.529) | 1-8 | 57.2 (56.6, 57.8) | 0.428 (0.422, 0.434) |
| Ranzani OT [122] 2022 | Case–control | CoronaVac/CoronaVac/BNT162b2 | 18-60 | Symptomatic COVID-19 | Brazil | Omicron (B.1.1.529) | >8 | 33.3 (32.5, 34.0) | 0.667 (0.66, 0.675) |
| Ranzani OT [122] 2022 | Case–control | CoronaVac/CoronaVac/CoronaVac | 18-60 | Symptomatic COVID-19 | Brazil | Omicron (B.1.1.529) | 1-8 | 22.1 (15.0, 28.5) | 0.779 (0.715, 0.850) |
| Ranzani OT [122] 2022 | Case–control | CoronaVac/CoronaVac/CoronaVac | 18-60 | Symptomatic COVID-19 | Brazil | Omicron (B.1.1.529) | >8 | 8.8 (4.5, 12.9) | 0.912 (0.871, 0.955) |
| Ranzani OT [122] 2022 | Case–control | CoronaVac/CoronaVac/BNT162b2 | 18-60 | Symptomatic COVID-19 | Brazil | Omicron (B.1.1.529) | 1-8 | 57.2 (55.7, 58.7) | 0.428 (0.413, 0.443) |
| Ranzani OT [122] 2022 | Case–control | CoronaVac/CoronaVac/BNT162b2 | 18-60 | Symptomatic COVID-19 | Brazil | Omicron (B.1.1.529) | >8 | 41.9 (40.2, 43.5) | 0.581 (0.565, 0.598) |
| Ranzani OT [122] 2022 | Case–control | CoronaVac/CoronaVac/CoronaVac | ≥ 75 | Symptomatic COVID-19 | Brazil | Omicron (B.1.1.529) | 1-8 | 38.7 (27.5, 48.2) | 0.613 (0.518, 0.725) |
| Ranzani OT [122] 2022 | Case–control | CoronaVac/CoronaVac/CoronaVac | ≥ 75 | Symptomatic COVID-19 | Brazil | Omicron (B.1.1.529) | >8 | 16.9 (12.2, 21.3) | 0.831 (0.787, 0.878) |
| Ranzani OT [122] 2022 | Case–control | CoronaVac/CoronaVac/BNT162b2 | ≥ 75 | Symptomatic COVID-19 | Brazil | Omicron (B.1.1.529) | 1-8 | 63.7 (61.2, 66.0) | 0.363 (0.34, 0.388) |
| Ranzani OT [122] 2022 | Case–control | CoronaVac/CoronaVac/BNT162b2 | ≥ 75 | Symptomatic COVID-19 | Brazil | Omicron (B.1.1.529) | >8 | 45.9 (43.4, 48.3) | 0.541 (0.517, 0.566) |
| Ranzani OT [122] 2022 | Case–control | CoronaVac/CoronaVac/CoronaVac | 18-60 | Severe COVID-19 | Brazil | Omicron (B.1.1.529) | 1-8 | 71.9 (41.9, 86.4) | 0.281 (0.136, 0.581) |
| Ranzani OT [122] 2022 | Case–control | CoronaVac/CoronaVac/CoronaVac | 18-60 | Severe COVID-19 | Brazil | Omicron (B.1.1.529) | >8 | 57.6 (15.1, 78.8) | 0.424 (0.212, 0.849) |
| Ranzani OT [122] 2022 | Case–control | CoronaVac/CoronaVac/BNT162b2 | 18-60 | Severe COVID-19 | Brazil | Omicron (B.1.1.529) | 1-8 | 90.8 (88.5, 92.7) | 0.092 (0.073, 0.115) |
| Ranzani OT [122] 2022 | Case–control | CoronaVac/CoronaVac/BNT162b2 | 18-60 | Severe COVID-19 | Brazil | Omicron (B.1.1.529) | >8 | 89.5 (87.5, 91.2) | 0.105 (0.088, 0.125) |
| Ranzani OT [122] 2022 | Case–control | CoronaVac/CoronaVac/CoronaVac | 60-74 | Severe COVID-19 | Brazil | Omicron (B.1.1.529) | 1-8 | 80.9 (69.4, 88.0) | 0.191 (0.12, 0.306) |
| Ranzani OT [122] 2022 | Case–control | CoronaVac/CoronaVac/CoronaVac | 60-74 | Severe COVID-19 | Brazil | Omicron (B.1.1.529) | >8 | 69.9 (62.9, 75.6) | 0.301 (0.244, 0.331) |
| Ranzani OT [122] 2022 | Case–control | CoronaVac/CoronaVac/BNT162b2 | 60-74 | Severe COVID-19 | Brazil | Omicron (B.1.1.529) | 1-8 | 86.1 (83.4, 88.4) | 0.139 (0.116, 0.166) |
| Ranzani OT [122] 2022 | Case–control | CoronaVac/CoronaVac/BNT162b2 | 60-74 | Severe COVID-19 | Brazil | Omicron (B.1.1.529) | >8 | 89.6 (88.2, 90.9) | 0.104 (0.091, 0.118) |
| Ranzani OT [122] 2022 | Case–control | CoronaVac/CoronaVac/CoronaVac | ≥ 75 | Severe COVID-19 | Brazil | Omicron (B.1.1.529) | 1-8 | 46.2 (3.5, 70.1) | 0.538 (0.299, 0.965) |
| Ranzani OT [122] 2022 | Case–control | CoronaVac/CoronaVac/CoronaVac | ≥ 75 | Severe COVID-19 | Brazil | Omicron (B.1.1.529) | >8 | 54.6 (47.9, 60.3) | 0.454 (0.397, 0.521) |
| Ranzani OT [122] 2022 | Case–control | CoronaVac/CoronaVac/BNT162b2 | ≥ 75 | Severe COVID-19 | Brazil | Omicron (B.1.1.529) | 1-8 | 80.2 (76.3, 83.5) | 0.198 (0.165, 0.237) |
| Ranzani OT [122] 2022 | Case–control | CoronaVac/CoronaVac/BNT162b2 | ≥ 75 | Severe COVID-19 | Brazil | Omicron (B.1.1.529) | >8 | 79.9 (77.5, 82.0) | 0.201 (0.180, 0.225) |
| Ranzani OT [122] 2022 | Case–control | CoronaVac/CoronaVac/CoronaVac | ≥ 18 | Severe COVID-19 | Brazil | Omicron (B.1.1.529) | 1-8 | 71.3 (60.3, 79.2) | 0.287 (0.208, 0.397) |
| Ranzani OT [122] 2022 | Case–control | CoronaVac/CoronaVac/CoronaVac | ≥ 18 | Severe COVID-19 | Brazil | Omicron (B.1.1.529) | >8 | 65.4 (61.5, 68.8) | 0.346 (0.312, 0.385) |
| Ranzani OT [122] 2022 | Case–control | CoronaVac/CoronaVac/BNT162b2 | ≥ 18 | Severe COVID-19 | Brazil | Omicron (B.1.1.529) | 1-8 | 85.5 (83.8, 87.0) | 0.145 (0.130, 0.162) |
| Ranzani OT [122] 2022 | Case–control | CoronaVac/CoronaVac/BNT162b2 | ≥ 18 | Severe COVID-19 | Brazil | Omicron (B.1.1.529) | >8 | 86.1 (85.0, 87.1) | 0.139 (0.129, 0.150) |
| Šmíd M [63] 2022 | Cohort study | BNT162b2 or mRNA-1273 | > 0 | Severe COVID-19 | Czech | Omicron (B.1.1.529) | 2-11 | 87 (84, 88) | 0.13 (0.12, 0.16) |
| Šmíd M [63] 2022 | Cohort study | BNT162b2 or mRNA-1273 | > 0 | Severe COVID-19 | Czech | Omicron (B.1.1.529) | ≥ 11 | 79 (75, 83) | 0.21 (0.17, 0.25) |
| Stowe j [125] 2022 | Case–control | BNT162b2 or mRNA-1273 | 18-64 | Severe COVID-19 | UK | Omicron (B.1.1.529) | 1-2 | 80.1 (73.5, 85.1) | 0.199 (0.149, 0.265) |
| Stowe j [125] 2022 | Case–control | BNT162b2 or mRNA-1273 | 18-64 | Severe COVID-19 | UK | Omicron (B.1.1.529) | 2-5 | 82.4 (78.6, 85.6) | 0.176 (0.144, 0.214) |
| Stowe j [125] 2022 | Case–control | BNT162b2 or mRNA-1273 | 18-64 | Severe COVID-19 | UK | Omicron (B.1.1.529) | 5-10 | 72.7 (67.2, 77.2) | 0.273 (0.228, 0.328) |
| Stowe j [125] 2022 | Case–control | BNT162b2 or mRNA-1273 | 18-64 | Severe COVID-19 | UK | Omicron (B.1.1.529) | 10-15 | 66.9 (59.1, 73.3) | 0.331 (0.267, 0.409) |
| Stowe j [125] 2022 | Case–control | BNT162b2 or mRNA-1273 | 18-64 | Severe COVID-19 | UK | Omicron (B.1.1.529) | ≥15 | 53.6 (36.9, 65.9) | 0.464 (0.341, 0.631) |
| Stowe j [125] 2022 | Case–control | BNT162b2 or mRNA-1273 | 18-64 | Severe COVID-19 | UK | Omicron (B.1.1.529) | 1-2 | 75.3 (61.1, 84.3) | 0.247 (0.157, 0.389) |
| Stowe j [125] 2022 | Case–control | BNT162b2 or mRNA-1273 | 18-64 | Severe COVID-19 | UK | Omicron (B.1.1.529) | 2-5 | 72.7 (63.9, 79.3) | 0.273 (0.207, 0.361) |
| Stowe j [125] 2022 | Case–control | BNT162b2 or mRNA-1273 | 18-64 | Severe COVID-19 | UK | Omicron (B.1.1.529) | 5-10 | 62.6 (52, 70.9) | 0.374 (0.291, 0.480) |
| Stowe j [125] 2022 | Case–control | BNT162b2 or mRNA-1273 | 18-64 | Severe COVID-19 | UK | Omicron (B.1.1.529) | 10-15 | 44.8 (26.1, 58.8) | 0.552 (0.412, 0.739) |
| Stowe j [125] 2022 | Case–control | BNT162b2 or mRNA-1273 | 18-64 | Severe COVID-19 | UK | Omicron (B.1.1.529) | ≥15 | 11.7 (-36.5, 42.9) | 0.883 (0.571, 1.365) |
| Stowe j [125] 2022 | Case–control | BNT162b2 or mRNA-1273 | 18-64 | Severe COVID-19 | UK | Omicron (B.1.1.529) | 1-2 | 58.5 (39.3, 71.6) | 0.415 (0.284, 0.607) |
| Stowe j [125] 2022 | Case–control | BNT162b2 or mRNA-1273 | 18-64 | Severe COVID-19 | UK | Omicron (B.1.1.529) | 2-5 | 56.2 (47.3, 63.7) | 0.438 (0.363, 0.527) |
| Stowe j [125] 2022 | Case–control | BNT162b2 or mRNA-1273 | 18-64 | Severe COVID-19 | UK | Omicron (B.1.1.529) | 5-10 | 56.6 (49.6, 62.7) | 0.434 (0.373, 0.504) |
| Stowe j [125] 2022 | Case–control | BNT162b2 or mRNA-1273 | 18-64 | Severe COVID-19 | UK | Omicron (B.1.1.529) | 10-15 | 50.2 (40.0, 58.7) | 0.498 (0.413, 0.600) |
| Stowe j [125] 2022 | Case–control | BNT162b2 or mRNA-1273 | 18-64 | Severe COVID-19 | UK | Omicron (B.1.1.529) | ≥15 | 50.9 (34.3, 63.3) | 0.491 (0.367, 0.657) |
| Stowe j [125] 2022 | Case–control | BNT162b2 or mRNA-1273 | 18-64 | Severe COVID-19 | UK | Omicron (B.1.1.529) | 1-2 | 87.7 (79.9, 92.5) | 0.123 (0.075, 0.201) |
| Stowe j [125] 2022 | Case–control | BNT162b2 or mRNA-1273 | 18-64 | Severe COVID-19 | UK | Omicron (B.1.1.529) | 2-5 | 87.8 (84.3, 90.5) | 0.122 (0.095, 0.157) |
| Stowe j [125] 2022 | Case–control | BNT162b2 or mRNA-1273 | 18-64 | Severe COVID-19 | UK | Omicron (B.1.1.529) | 5-10 | 83.4 (80.0, 86.2) | 0.166 (0.138, 0.200) |
| Stowe j [125] 2022 | Case–control | BNT162b2 or mRNA-1273 | 18-64 | Severe COVID-19 | UK | Omicron (B.1.1.529) | 10-15 | 76.3 (70.8, 80.7) | 0.237 (0.193, 0.292) |
| Stowe j [125] 2022 | Case–control | BNT162b2 or mRNA-1273 | 18-64 | Severe COVID-19 | UK | Omicron (B.1.1.529) | ≥15 | 66.3 (53.6, 75.5) | 0.337 (0.245, 0.464) |
| Stowe j [125] 2022 | Case–control | BNT162b2 or mRNA-1273 | 18-64 | Severe COVID-19 | UK | Omicron (B.1.1.529) | 1-2 | 90.9 (83.2, 95.1) | 0.091 (0.049, 0.168) |
| Stowe j [125] 2022 | Case–control | BNT162b2 or mRNA-1273 | 18-64 | Severe COVID-19 | UK | Omicron (B.1.1.529) | 2-5 | 88.6 (84.9, 91.5) | 0.114 (0.085, 0.151) |
| Stowe j [125] 2022 | Case–control | BNT162b2 or mRNA-1273 | 18-64 | Severe COVID-19 | UK | Omicron (B.1.1.529) | 5-10 | 85.8 (82.4, 88.5) | 0.142 (0.115, 0.176) |
| Stowe j [125] 2022 | Case–control | BNT162b2 or mRNA-1273 | 18-64 | Severe COVID-19 | UK | Omicron (B.1.1.529) | 10-15 | 80.2 (74.9, 84.4) | 0.198 (0.156, 0.251) |
| Stowe j [125] 2022 | Case–control | BNT162b2 or mRNA-1273 | 18-64 | Severe COVID-19 | UK | Omicron (B.1.1.529) | ≥15 | 67.4 (53.1, 77.4) | 0.326 (0.226, 0.469) |
| Stowe j [125] 2022 | Case–control | BNT162b2 or mRNA-1273 | 18-64 | Severe COVID-19 | UK | Omicron (B.1.1.529) | 1-2 | 95.0 (89.1, 97.7) | 0.05 (0.023, 0.109) |
| Stowe j [125] 2022 | Case–control | BNT162b2 or mRNA-1273 | 18-64 | Severe COVID-19 | UK | Omicron (B.1.1.529) | 2-5 | 89.8 (85.9, 92.6) | 0.102 (0.074, 0.141) |
| Stowe j [125] 2022 | Case–control | BNT162b2 or mRNA-1273 | 18-64 | Severe COVID-19 | UK | Omicron (B.1.1.529) | 5-10 | 87.8 (84.3, 90.4) | 0.122 (0.096, 0.157) |
| Stowe j [125] 2022 | Case–control | BNT162b2 or mRNA-1273 | 18-64 | Severe COVID-19 | UK | Omicron (B.1.1.529) | 10-15 | 80.4 (74.5, 85.0) | 0.196 (0.150, 0.255) |
| Stowe j [125] 2022 | Case–control | BNT162b2 or mRNA-1273 | 18-64 | Severe COVID-19 | UK | Omicron (B.1.1.529) | ≥15 | 68.6 (52.3, 79.4) | 0.314 (0.206, 0.477) |
| Stowe j [125] 2022 | Case–control | BNT162b2 or mRNA-1273 | 18-64 | Severe COVID-19 | UK | Omicron (B.1.1.529) | 2-5 | 94.2 (76.6, 98.6) | 0.058 (0.014, 0.234) |
| Stowe j [125] 2022 | Case–control | BNT162b2 or mRNA-1273 | 18-64 | Severe COVID-19 | UK | Omicron (B.1.1.529) | 5-10 | 93.9 (81.6, 97.9) | 0.061 (0.021, 0.184) |
| Stowe j [125] 2022 | Case–control | BNT162b2 or mRNA-1273 | 18-64 | Severe COVID-19 | UK | Omicron (B.1.1.529) | 10-15 | 94.0 (78.8, 98.3) | 0.06 (0.017, 0.212) |
| Stowe j [125] 2022 | Case–control | BNT162b2 or mRNA-1273 | 18-64 | Severe COVID-19 | UK | Omicron (B.1.1.529) | ≥15 | 80.4 (-36.3, 97.2) | 0.196 (0.028, 1.363) |
| Stowe j [125] 2022 | Case–control | BNT162b2 or mRNA-1273 | 18-64 | Severe COVID-19 | UK | Omicron (B.1.1.529) | 2-5 | 97.1 (92.2, 98.9) | 0.029 (0.011, 0.078) |
| Stowe j [125] 2022 | Case–control | BNT162b2 or mRNA-1273 | 18-64 | Severe COVID-19 | UK | Omicron (B.1.1.529) | 5-10 | 94.3 (88.9, 97.1) | 0.057 (0.029, 0.111) |
| Stowe j [125] 2022 | Case–control | BNT162b2 or mRNA-1273 | 18-64 | Severe COVID-19 | UK | Omicron (B.1.1.529) | 10-15 | 89.9 (78.3, 95.3) | 0.101 (0.047, 0.217) |
| Stowe j [125] 2022 | Case–control | BNT162b2 or mRNA-1273 | 18-64 | Severe COVID-19 | UK | Omicron (B.1.1.529) | ≥15 | 75.9 (15.8, 93.1) | 0.241 (0.069, 0.842) |
| Stowe j [125] 2022 | Case–control | BNT162b2 or mRNA-1273 | ≥ 65 | Severe COVID-19 | UK | Omicron (B.1.1.529) | 1-2 | 92.3 (76.3, 97.5) | 0.073 (0.025, 0.237) |
| Stowe j [125] 2022 | Case–control | BNT162b2 or mRNA-1273 | ≥ 65 | Severe COVID-19 | UK | Omicron (B.1.1.529) | 2-5 | 92.4 (86.0, 95.8) | 0.076 (0.042, 0.14) |
| Stowe j [125] 2022 | Case–control | BNT162b2 or mRNA-1273 | ≥ 65 | Severe COVID-19 | UK | Omicron (B.1.1.529) | 5-10 | 87.0 (79.2, 91.8) | 0.13 (0.082, 0.208) |
| Stowe j [125] 2022 | Case–control | BNT162b2 or mRNA-1273 | ≥ 65 | Severe COVID-19 | UK | Omicron (B.1.1.529) | 10-15 | 84.0 (74.6, 89.9) | 0.16 (0.101, 0.254) |
| Stowe j [125] 2022 | Case–control | BNT162b2 or mRNA-1273 | ≥ 65 | Severe COVID-19 | UK | Omicron (B.1.1.529) | ≥15 | 76.9 (60.6, 86.4) | 0.231 (0.136, 0.394) |
| Stowe j [125] 2022 | Case–control | BNT162b2 or mRNA-1273 | ≥ 65 | Severe COVID-19 | UK | Omicron (B.1.1.529) | 1-2 | 69.2 (-18.7, 92.0) | 0.308 (0.08, 1.187) |
| Stowe j [125] 2022 | Case–control | BNT162b2 or mRNA-1273 | ≥ 65 | Severe COVID-19 | UK | Omicron (B.1.1.529) | 2-5 | 87.4 (72.5, 94.2) | 0.126 (0.058, 0.275) |
| Stowe j [125] 2022 | Case–control | BNT162b2 or mRNA-1273 | ≥ 65 | Severe COVID-19 | UK | Omicron (B.1.1.529) | 5-10 | 79.3 (65.7, 87.5) | 0.207 (0.125, 0.343) |
| Stowe j [125] 2022 | Case–control | BNT162b2 or mRNA-1273 | ≥ 65 | Severe COVID-19 | UK | Omicron (B.1.1.529) | 10-15 | 67.2 (46.6, 79.8) | 0.228 (0.202, 0.534) |
| Stowe j [125] 2022 | Case–control | BNT162b2 or mRNA-1273 | ≥ 65 | Severe COVID-19 | UK | Omicron (B.1.1.529) | ≥15 | 59.0 (30.5, 75.8) | 0.41 (0.242, 0.695) |
| Stowe j [125] 2022 | Case–control | BNT162b2 or mRNA-1273 | ≥ 65 | Severe COVID-19 | UK | Omicron (B.1.1.529) | 1-2 | 62.5 (40.7, 76.4) | 0.375 (0.236, 0.593) |
| Stowe j [125] 2022 | Case–control | BNT162b2 or mRNA-1273 | ≥ 65 | Severe COVID-19 | UK | Omicron (B.1.1.529) | 2-5 | 69.3 (60.8, 76.0) | 0.307 (0.240, 0.392) |
| Stowe j [125] 2022 | Case–control | BNT162b2 or mRNA-1273 | ≥ 65 | Severe COVID-19 | UK | Omicron (B.1.1.529) | 5-10 | 67.2 (60.5, 72.8) | 0.228 (0.272, 0.395) |
| Stowe j [125] 2022 | Case–control | BNT162b2 or mRNA-1273 | ≥ 65 | Severe COVID-19 | UK | Omicron (B.1.1.529) | 10-15 | 59.4 (51.5, 66.0) | 0.406 (0.34, 0.485) |
| Stowe j [125] 2022 | Case–control | BNT162b2 or mRNA-1273 | ≥ 65 | Severe COVID-19 | UK | Omicron (B.1.1.529) | ≥15 | 56.3 (46.9, 64.0) | 0.437 (0.36, 0.531) |
| Stowe j [125] 2022 | Case–control | BNT162b2 or mRNA-1273 | ≥ 65 | Severe COVID-19 | UK | Omicron (B.1.1.529) | 1-2 | 82.2 (73.0, 88.3) | 0.178 (0.117, 0.27) |
| Stowe j [125] 2022 | Case–control | BNT162b2 or mRNA-1273 | ≥ 65 | Severe COVID-19 | UK | Omicron (B.1.1.529) | 2-5 | 91.3 (89.1, 93.0) | 0.087 (0.07, 0.109) |
| Stowe j [125] 2022 | Case–control | BNT162b2 or mRNA-1273 | ≥ 65 | Severe COVID-19 | UK | Omicron (B.1.1.529) | 5-10 | 88.9 (87.1, 90.6) | 0.111 (0.094, 0.129) |
| Stowe j [125] 2022 | Case–control | BNT162b2 or mRNA-1273 | ≥ 65 | Severe COVID-19 | UK | Omicron (B.1.1.529) | 10-15 | 87.6 (85.6, 89.3) | 0.124 (0.107, 0.144) |
| Stowe j [125] 2022 | Case–control | BNT162b2 or mRNA-1273 | ≥ 65 | Severe COVID-19 | UK | Omicron (B.1.1.529) | ≥15 | 84.1 (81.2, 86.5) | 0.159 (0.135, 0.188) |
| Stowe j [125] 2022 | Case–control | BNT162b2 or mRNA-1273 | ≥ 65 | Severe COVID-19 | UK | Omicron (B.1.1.529) | 1-2 | 84.7 (76.0, 90.2) | 0.153 (0.098, 0.24) |
| Stowe j [125] 2022 | Case–control | BNT162b2 or mRNA-1273 | ≥ 65 | Severe COVID-19 | UK | Omicron (B.1.1.529) | 2-5 | 91.3 (89.1, 93.1) | 0.087 (0.069, 0.109) |
| Stowe j [125] 2022 | Case–control | BNT162b2 or mRNA-1273 | ≥ 65 | Severe COVID-19 | UK | Omicron (B.1.1.529) | 5-10 | 89.3 (87.3, 90.9) | 0.107 (0.091, 0.127) |
| Stowe j [125] 2022 | Case–control | BNT162b2 or mRNA-1273 | ≥ 65 | Severe COVID-19 | UK | Omicron (B.1.1.529) | 10-15 | 88.1 (86.1, 89.9) | 0.119 (0.101, 0.139) |
| Stowe j [125] 2022 | Case–control | BNT162b2 or mRNA-1273 | ≥ 65 | Severe COVID-19 | UK | Omicron (B.1.1.529) | ≥15 | 85.3 (82.4, 87.6) | 0.147 (0.124, 0.176) |
| Stowe j [125] 2022 | Case–control | BNT162b2 or mRNA-1273 | ≥ 65 | Severe COVID-19 | UK | Omicron (B.1.1.529) | 1-2 | 84.5 (75.5, 90.2) | 0.155 (0.098, 0.245) |
| Stowe j [125] 2022 | Case–control | BNT162b2 or mRNA-1273 | ≥ 65 | Severe COVID-19 | UK | Omicron (B.1.1.529) | 2-5 | 91.4 (89.0, 93.2) | 0.086 (0.068, 0.11) |
| Stowe j [125] 2022 | Case–control | BNT162b2 or mRNA-1273 | ≥ 65 | Severe COVID-19 | UK | Omicron (B.1.1.529) | 5-10 | 89.5 (87.5, 91.2) | 0.105 (0.088, 0.125) |
| Stowe j [125] 2022 | Case–control | BNT162b2 or mRNA-1273 | ≥ 65 | Severe COVID-19 | UK | Omicron (B.1.1.529) | 10-15 | 88.6 (86.5, 90.3) | 0.114 (0.097, 0.135) |
| Stowe j [125] 2022 | Case–control | BNT162b2 or mRNA-1273 | ≥ 65 | Severe COVID-19 | UK | Omicron (B.1.1.529) | ≥15 | 86.4 (83.6, 88.7) | 0.136 (0.113, 0.164) |
| Stowe j [125] 2022 | Case–control | BNT162b2 or mRNA-1273 | ≥ 65 | Severe COVID-19 | UK | Omicron (B.1.1.529) | 1-2 | 86.7 (-13.9, 98.5) | 0.133 (0.015, 1.139) |
| Stowe j [125] 2022 | Case–control | BNT162b2 or mRNA-1273 | ≥ 65 | Severe COVID-19 | UK | Omicron (B.1.1.529) | 2-5 | 95.9 (89.0, 98.4) | 0.041 (0.016, 0.110) |
| Stowe j [125] 2022 | Case–control | BNT162b2 or mRNA-1273 | ≥ 65 | Severe COVID-19 | UK | Omicron (B.1.1.529) | 5-10 | 93.9 (88.4, 96.8) | 0.061 (0.032, 0.116) |
| Stowe j [125] 2022 | Case–control | BNT162b2 or mRNA-1273 | ≥ 65 | Severe COVID-19 | UK | Omicron (B.1.1.529) | 10-15 | 93.2 (87.5, 96.2) | 0.068 (0.038, 0.125) |
| Stowe j [125] 2022 | Case–control | BNT162b2 or mRNA-1273 | ≥ 65 | Severe COVID-19 | UK | Omicron (B.1.1.529) | ≥15 | 90.1 (79.7, 95.2) | 0.091 (0.048, 0.203) |
| Stowe j [125] 2022 | Case–control | BNT162b2 or mRNA-1273 | ≥ 65 | Severe COVID-19 | UK | Omicron (B.1.1.529) | 1-2 | 94.7 (71.6, 99.0) | 0.053 (0.01, 0.284) |
| Stowe j [125] 2022 | Case–control | BNT162b2 or mRNA-1273 | ≥ 65 | Severe COVID-19 | UK | Omicron (B.1.1.529) | 2-5 | 95.8 (91.3, 97.9) | 0.042 (0.021, 0.087) |
| Stowe j [125] 2022 | Case–control | BNT162b2 or mRNA-1273 | ≥ 65 | Severe COVID-19 | UK | Omicron (B.1.1.529) | 5-10 | 92.8 (88.4, 95.6) | 0.072 (0.044, 0.116) |
| Stowe j [125] 2022 | Case–control | BNT162b2 or mRNA-1273 | ≥ 65 | Severe COVID-19 | UK | Omicron (B.1.1.529) | 10-15 | 92.5 (88.1, 95.2) | 0.075 (0.048, 0.119) |
| Stowe j [125] 2022 | Case–control | BNT162b2 or mRNA-1273 | ≥ 65 | Severe COVID-19 | UK | Omicron (B.1.1.529) | ≥15 | 86.8 (77.1, 92.3) | 0.132 (0.077, 0.229) |
| Tartof SY [124] 2022 | Case–control | BNT162b2/BNT162b2/BNT162b2 | ≥18 | Severe COVID-19 | USA | Omicron (B.1.1.529) | 2-15 | 85 (80, 89) | 0.15 (0.11, 0.20) |
| Tartof SY [124] 2022 | Case–control | BNT162b2/BNT162b2/BNT162b2 | ≥18 | Severe COVID-19 | USA | Omicron (B.1.1.529) | ≥15 | 55 (28, 71) | 0.45 (0.29, 0.72) |
| Tseng HF [88] 2022 | Case–control | mRNA-1273/mRNA-1273/mRNA-1273 | ≥18 | SARS-CoV-2 infection | USA | Omicron (B.1.1.529) | 2-8 | 71.6 (69.7, 73.4) | 0.284 (0.266, 0.303) |
| Tseng HF [88] 2022 | Case–control | mRNA-1273/mRNA-1273/mRNA-1273 | ≥18 | SARS-CoV-2 infection | USA | Omicron (B.1.1.529) | > 8 | 47.4 (40.5, 53.5) | 0.526 (0.465, 0.595) |
