# Supplementary material for: A comprehensive analysis of the efficacy and effectiveness of COVID-19 vaccines
Source: Front Immunol. 2022 Aug 26;13:945930. doi: 10.3389/fimmu.2022.945930 (PMC9459021; doi:10.3389/fimmu.2022.945930)
Supplement: Supplementary file 9 [file Table_8.docx]

**Supplementary Table 8** Risk of bias assessment using the ROBINS-2 tool for randomized control study

| **First author/year** | **Risk of bias arising from randomization process** | **Risk of bias due to deviations from intended interventions** | **Missing outcome data** | **Risk of bias in measurement of outcome** | **Risk of bias in selection of reported result** | **Overall bias** |
| --- | --- | --- | --- | --- | --- | --- |
| Al Kaabi N [31] 2021 | Low | Low | Low | Low | Low | Low |
| Ali K [33] 2021 | Low | Low | Low | Low | Low | Low |
| Baden LR [116] 2020 | Low | Low | Low | Low | Low | Low |
| Baden LR [51] 2021 | Low | Low | Low | Low | Low | Low |
| Bravo L [52] 2022 | Low | Low | Low | Low | Low | Low |
| Clemens SAC [43] 2021 | Low | Low | Low | Low | Low | Low |
| Dunkle LM [49] 2022 | Low | Low | Low | Low | Low | Low |
| El Sahly HM [41] 2021 | Low | Low | Low | Low | Low | Low |
| Ella R [32] 2021 | Low | Low | Low | Low | Low | Low |
| Fadlyana E [42] 2021 | Low | Low | Low | Low | Low | Low |
| Falsey AR [34] 2021 | Low | Low | Low | Low | Low | Low |
| Frenck RW Jr [35] 2021 | Low | Low | Low | Low | Low | Low |
| Halperin SA [50] 2022 | Low | Low | Low | Low | Low | Low |
| Heath PT [36] 2021 | Low | Low | Low | Low | Low | Low |
| Kremsner PG [45] 2022 | Low | Low | Low | Low | Low | Low |
| Logunov DY [30] 2021 | Low | Low | Low | Low | Low | Low |
| Madhi SA [40] 2021 | Low | Low | Low | Low | Low | Low |
| Palacios R [44] 2021 | Low | Low | Low | Low | Low | Low |
| Polack FP [45] 2020 | Low | Low | Low | Low | Low | Low |
| Sadoff J [37] 2021 | Low | Low | Low | Low | Low | Low |
| Sadoff J [53] 2022 | Low | Low | Low | Low | Low | Low |
| Shinde V [54] 2021 | Low | Low | Low | Low | Low | Low |
| Tanriover MD [38] 2021 | Low | Low | Low | Low | Low | Low |
| Thomas SJ [39] 2021 | Low | Low | Low | Low | Low | Low |
| Thomas SJ [56] 2022 | Low | Low | Low | Low | Low | Low |
| US FDA [48] 2022 | Low | Low | Low | Low | Low | Low |
| Voysey M [29] 2021 | Low | Low | Low | Low | Low | Low |
| Voysey M [47] 2021 | Low | Low | Low | Low | Low | Low |
| Walter EB [46] 2022 | Low | Low | Low | Low | Low | Low |
