# Supplementary material for: A comprehensive analysis of the efficacy and effectiveness of COVID-19 vaccines
Source: Front Immunol. 2022 Aug 26;13:945930. doi: 10.3389/fimmu.2022.945930 (PMC9459021; doi:10.3389/fimmu.2022.945930)
Supplement: Supplementary file 10 [file Table_9.docx]

**Supplementary Table 9** Risk of bias assessment using the ROBINS-I tool for observational studies

| **First author/year** | **Bias in confounding** | **Bias in selection of participants into the study at intervention** | **Bias in classification of interventions** | **Bias in deviation from intended interventions** | **Bias in missing data** | **Bias in measurement of outcomes** | **Bias in selection of reported results** | **Overall bias** |
| --- | --- | --- | --- | --- | --- | --- | --- | --- |
| Accorsi EK [89] 2022 | Moderate | Low | Low | Low | Low | Low | Low | Moderate |
| Andeweg SP [75] 2022 | Serious | Low | Low | Low | Low | Moderate | Moderate | **Serious** |
| Andrews N [105] 2021 | Moderate | Low | Low | Low | Low | Moderate | Moderate | Moderate |
| Andrews N [97] 2022 | Moderate | Low | Low | Low | Low | Moderate | Moderate | Moderate |
| Barda N [93] 2021 | Moderate | Low | Low | Low | Low | Moderate | Low | Moderate |
| Bar-On YM [72] 2022 | Moderate | Low | Low | Low | Low | Moderate | Low | Moderate |
| Baum U [123] 2022 | Moderate | Low | Low | Low | Low | Low | Moderate | Moderate |
| Bruxvoort KJ [66] 2021 | Moderate | Moderate | Low | Low | Low | Moderate | Moderate | Moderate |
| Buchan SA [120] 2022 | Moderate | Moderate | Low | Low | Low | Moderate | Moderate | Moderate |
| Buchan SA [98] 2021 | Moderate | Moderate | Low | Low | Low | Moderate | Moderate | Moderate |
| Chemaitelly H [121] 2022 | Moderate | Low | Low | Low | Low | Low | Moderate | Moderate |
| Chin ET [69] 2021 | Moderate | Low | Low | Low | Low | Moderate | Moderate | Moderate |
| Cohen MJ [118] 2022 | Moderate | Low | Low | Low | Low | Moderate | Moderate | Moderate |
| Collie S [59] 2022 | Moderate | Low | Low | Low | Low | Moderate | Moderate | Moderate |
| Dorabawila V [68] 2022 | Serious | Low | Low | Low | Low | Low | Moderate | **Serious** |
| Fabiani M [78] 2022 | Moderate | Low | Low | Low | Low | Low | Low | Moderate |
| Ferdinands JM [85] 2022 | Moderate | Low | Low | Low | Low | Low | Moderate | Moderate |
| Fowlkes A [79] 2021 | Moderate | Low | Low | Low | Low | Low | Moderate | Moderate |
| Fowlkes AL [119] 2022 | Moderate | Low | Low | Low | Low | Low | Moderate | Moderate |
| Goldberg Y [106] 2021 | Serious | Low | Low | Low | Low | Low | Moderate | **Serious** |
| Gray GE [76] 2021 | Moderate | Low | Low | Low | Low | Low | Moderate | Moderate |
| Hansen CH [64] 2021 | Moderate | Low | Low | Low | Low | Low | Moderate | Moderate |
| Israel A [114] 2021 | Moderate | Low | Low | Low | Low | Low | Moderate | Moderate |
| Kang M [83] 2022 | Moderate | Low | Low | Low | Low | Low | Moderate | Moderate |
| Kirsebom FCM [111] 2022 | Moderate | Low | Low | Low | Low | Low | Moderate | Moderate |
| Kiss Z [108] 2022 | Moderate | Low | Low | Low | Low | Low | Moderate | Moderate |
| Klein NP [57] 2022 | Moderate | Low | Low | Low | Low | Low | Moderate | Moderate |
| Lauring AS [71] 2022 | Moderate | Low | Low | Low | Low | Low | Moderate | Moderate |
| Li XN [61] 2021 | Moderate | Low | Low | Low | Low | Low | Moderate | Moderate |
| Lopez Bernal J [65] 2021 | Moderate | Low | Low | Low | Low | Low | Moderate | Moderate |
| Martínez-Baz I [101] 2021 | Serious | Serious | Low | Low | Low | Low | Moderate | **Serious** |
| McMenamin ME [55] 2022 | Moderate | Low | Low | Low | Low | Low | Moderate | Moderate |
| Mizrahi B [113] 2021 | Serious | Low | Low | Low | Low | Low | Moderate | **Serious** |
| Nanduri S [87] 2021 | Moderate | Low | Low | Low | Low | Low | Moderate | Moderate |
| Nasreen S [74] 2021 | Moderate | Low | Low | Low | Low | Low | Low | Moderate |
| Norddahl GL [70] 2022 | Moderate | Low | Low | Low | Low | Low | Moderate | Moderate |
| Nordström P [94] 2021 | Moderate | Low | Low | Low | Low | Moderate | Moderate | Moderate |
| Nunes MC [77] 2022 | Moderate | Low | Low | Low | Low | Low | Moderate | Moderate |
| Patalon T [126] 2022 | Moderate | Low | Low | Low | Low | Low | Low | Moderate |
| Poukka E [102] 2021 | Serious | Low | Low | Low | Low | Low | Moderate | **Serious** |
| Pouwels KB [86] 2021 | Moderate | Low | Low | Low | Low | Low | Moderate | Moderate |
| Powell AA [62] 2021 | Moderate | Low | Low | Low | Low | Low | Low | Moderate |
| Price AM [107] 2022 | Moderate | Low | Low | Low | Low | Low | Moderate | Moderate |
| Ranzani OT [122] 2022 | Moderate | Low | Low | Low | Low | Low | Low | Moderate |
| Regev-Yochay G [67] 2021 | Moderate | Low | Low | Low | Low | Low | Low | Moderate |
| Reis BY [104] 2021 | Moderate | Low | Low | Low | Low | Low | Moderate | Moderate |
| Rosenberg ES [115] 2022 | Serious | Low | Low | Low | Moderate | Low | Moderate | **Serious** |
| Saciuk Y [92] 2022 | Moderate | Low | Low | Low | Low | Low | Low | Moderate |
| Seppälä E [84] 2021 | Moderate | Low | Low | Low | Low | Low | Moderate | Moderate |
| Sheikh A [82] 2021 | Moderate | Low | Low | Low | Low | Low | Moderate | Moderate |
| Skowronski DM [103] 2021 | Serious | Low | Low | Low | Low | Low | Moderate | **Serious** |
| Šmíd M [63] 2022 | Moderate | Low | Low | Low | Low | Low | Moderate | Moderate |
| Spensley K [99] 2022 | Moderate | Low | Low | Low | Low | Low | Moderate | Moderate |
| Spitzer A [90] 2022 | Moderate | Low | Low | Low | Low | Low | Moderate | Moderate |
| Sritipsukho P [81] 2022 | Moderate | Low | Low | Low | Low | Low | Moderate | Moderate |
| Starrfelt J [109] 2022 | Moderate | Low | Low | Low | Low | Low | Moderate | Moderate |
| Stowe j [125] 2022 | Moderate | Low | Low | Low | Low | Low | Moderate | Moderate |
| Tai CG [100] 2022 | Moderate | Low | Low | Low | Low | Low | Low | Moderate |
| Tan SHX [96] 2022 | Moderate | Low | Low | Low | Low | Low | Moderate | Moderate |
| Tang P [60] 2021 | Moderate | Low | Low | Low | Low | Low | Low | Moderate |
| Tartof SY [124] 2022 | Moderate | Low | Low | Low | Low | Moderate | Low | Moderate |
| Tartof SY [80] 2021 | Moderate | Low | Low | Low | Low | Moderate | Low | Moderate |
| Tenforde MW [95] 2022 | Moderate | Low | Low | Low | Low | Low | Low | Moderate |
| Thiruvengadam R [58] 2022 | Moderate | Low | Low | Low | Low | Low | Moderate | Moderate |
| Thompson MG [91] 2022 | Moderate | Low | Low | Low | Low | Low | Low | Moderate |
| Tseng HF [88] 2022 | Moderate | Low | Low | Low | Low | Low | Low | Moderate |
| Veneti L [100] 2022 | Moderate | Low | Low | Low | Low | Low | Moderate | Moderate |
| Yinong [73] 2022 | Moderate | Low | Low | Low | Low | Low | Moderate | Moderate |
| Yoon SK [112] 2022 | Moderate | Low | Low | Low | Low | Low | Moderate | Moderate |
