# Supplementary material for: A comprehensive analysis of the efficacy and effectiveness of COVID-19 vaccines
Source: Front Immunol. 2022 Aug 26;13:945930. doi: 10.3389/fimmu.2022.945930 (PMC9459021; doi:10.3389/fimmu.2022.945930)
Supplement: Supplementary file 11 [file Table_10.docx]

**Supplementary Table 10** The duration of effectiveness of COVID-19 vaccines against SARS-CoV-2 Omicron (B.1.1.529) variant infection

| **Variant** | **No. of studies** | **Adjust RR/OR (95% CI)** | ***P*_h_/*I*^2^ (%)** ^&^ | **Time interval of after full vaccination (week)** | **VE (%) (95% CI) ^#^** | **Vaccine name** | **Types of vaccine** |
| --- | --- | --- | --- | --- | --- | --- | --- |
| **Case-control studies** | | | | | | | |
| Overall | 1 | 0.560 (0.484, 0.649) | NA | 2-12 | 44.0 (35.1, 51.6) | mRNA-1273 | RNA-based vaccine |
|  | 1 | 0.765 (0.700, 0.836) | NA | 12-26 | 23.5 (16.4, 30.0) |  |  |
|  | 1 | 0.862 (0.827, 0.898) | NA | 26-39 | 13.8 (10.2, 17.3) |  |  |
|  | 1 | 0.941 (0.890, 0.996) | NA | ≥39 | 5.90 (0.40, 11.0) |  |  |
| **Cohort studies** | | | | | | | |
| Overall | 9 | 0.343 (0.268, 0.439) | <0.001/99.6 | 1-6 | 65.7 (56.1, 73.2) | BNT162b2 | RNA-based vaccine |
|  | 12 | 0.585 (0.514, 0.666) | <0.001/99.0 | 6-11 | 41.5 (33.4, 48.6) |  |  |
|  | 1 | 0.902 (0.739, 1.10) | NA | 11-15 | 9.8 (−10.0, 26.1) |  |  |
|  | 1 | 1.765 (1.595, 1.953) | NA | 15-24 | −76.5 (−95.3, −59.5) |  |  |
| 5-11 years | 4 | 0.444 (0.393, 0.501) | <0.001/95.8 | 1-6 | 55.6 (49.9, 60.7) | BNT162b2 | RNA-based vaccine |
|  | 3 | 0.774 (0.654, 0.916) | <0.001/97.6 | 6-11 | 22.6 (8.40, 34.6) |  |  |
| 12-17 years | 5 | 0.283 (0.185, 0.423) | <0.001/99.7 | 1-6 | 71.7 (57.7, 81.5) |  |  |
|  | 7 | 0.488 (0.467, 0.511) | <0.001/88.5 | 6-11 | 51.2 (48.9, 53.3) |  |  |
| Overall | 1 | 0.633 (0.236, 1.699) | NA | 2-6 | 36.7 (−69.9, 76.4) | mRNA-1273 | RNA-based vaccine |
|  | 1 | 0.700 (0.346, 1.413) | NA | 6-11 | 30.0 (−41.3, 65.4) |  |  |
|  | 1 | 0.958 (0.702, 1.308) | NA | 11-15 | 4.2 (−30.8, 29.8) |  |  |
|  | 1 | 1.393 (1.200, 1.616) | NA | 15-24 | −39.3 (−61.6, −20.0) |  |  |

^#^ Vaccine effectiveness = 100*(1–RR/OR) %

^&^ NA = not available
