# Supplementary material for: A comprehensive analysis of the efficacy and effectiveness of COVID-19 vaccines
Source: Front Immunol. 2022 Aug 26;13:945930. doi: 10.3389/fimmu.2022.945930 (PMC9459021; doi:10.3389/fimmu.2022.945930)
Supplement: Supplementary file 12 [file Table_11.docx]

**Supplementary Table 11** The duration of effectiveness of COVID-19 vaccines against symptomatic COVID-19 caused by Omicron (B.1.1.529) variant

| **Variant** | **No. of studies** | **Adjust OR (95% CI)** | ***P*_h_/*I*^2^ (%)** ^&^ | **Time interval of after full vaccination (week)** | **VE (%) (95% CI) ^#^** | **Vaccine name** | **Types of vaccine** |
| --- | --- | --- | --- | --- | --- | --- | --- |
| Overall | 6 | 0.410 (0.326, 0.516) | <0.001/98.7 | 1-10 | 59.0 (48.4, 67.4) | BNT162b2 | RNA-based vaccine |
|  | 5 | 0.690 (0.605, 0.788) | <0.001/98.5 | 8-20 | 31.0 (21.2, 39.5) |  |  |
|  | 4 | 0.803 (0.708, 0.911) | <0.001/84.3 | 17-26 | 19.7 (8.9, 29.2) |  |  |
|  | 3 | 0.943 (0.821, 1.082) | <0.001/89.1 | 26-38 | 5.7 (−8.2, 17.9) |  |  |
|  | 3 | 1.054 (0.934, 1.190) | <0.001/86.4 | 38-51 | −5.4 (−19.0, 6.6) |  |  |
| Overall | 5 | 0.502 (0.393, 0.641) | <0.001/98.5 | 2-20 | 49.8 (35.9, 60.7) | mRNA-1273 | RNA-based vaccine |
|  | 1 | 0.850 (0.818, 0.884) | NA | 20-25 | 15.0 (11.6, 18.2) |  |  |
|  | 2 | 0.970 (0.759, 1.239) | <0.001/92.2 | ≥25 | 3.0 (−23.9, 24.1) |  |  |
| Overall | 1 | 0.511 (0.429, 0.608) | NA | 2-5 | 48.9 (39.2, 57.1) | ChAdOx1 nCoV-19 | Viral vector (non-replicating) |
|  | 1 | 0.663 (0.585, 0.750) | NA | 5-10 | 33.7 (25.0, 41.5) |  |  |
|  | 1 | 0.714 (0.644, 0.791) | NA | 10-15 | 28.6 (20.9, 35.6) |  |  |
|  | 1 | 0.822 (0.781, 0.866) | NA | 15-20 | 17.8 (13.4, 21.9) |  |  |
|  | 1 | 0.96 (0.939, 0.981) | NA | 20-25 | 4.0 (1.9, 6.1) |  |  |
|  | 1 | 1.027 (1.012, 1.042) | NA | ≥25 | −2.7 (−4.2, −1.2) |  |  |
| Overall | 1 | 0.731 (0.714, 0.749) | NA | 2-8 | 26.9 (25.1, 28.6) | CoronaVac | Inactivated virus |
|  | 1 | 0.950 (0.941, 0.958) | NA | 8-26 | 5.0 (4.2, 5.9) |  |  |
|  | 1 | 0.919 (0.909, 0.93) | NA | ≥26 | 8.1 (7.0, 9.1) |  |  |
| 18-60 year | 1 | 0.963 (0.950, 0.975) | NA | ≥26 | 3.7 (2.5, 5.0) |  |  |
| 60-74 year | 1 | 0.782 (0.758, 0.817) | NA | ≥26 | 21.8 (19.3, 24.2) |  |  |
| ≥75 year | 1 | 0.728 (0.693, 0.765) | NA | ≥26 | 27.2 (23.5, 30.7) |  |  |

^#^ Vaccine effectiveness = 100*(1–RR/OR) %

^&^ NA = not available
