# Supplementary material for: A comprehensive analysis of the efficacy and effectiveness of COVID-19 vaccines
Source: Front Immunol. 2022 Aug 26;13:945930. doi: 10.3389/fimmu.2022.945930 (PMC9459021; doi:10.3389/fimmu.2022.945930)
Supplement: Supplementary file 13 [file Table_12.docx]

**Supplementary Table 12** The duration of effectiveness of COVID-19 vaccine booster immunization against symptomatic COVID-19 caused by Omicron variant

| **Variant** | **No. of studies** | **Adjust OR (95% CI)** | ***P*_h_/*I*^2^ (%)** ^&^ | **Time interval (week)** | **VE (%) (95% CI) ^#^** | **Vaccine name** | **Types of booster vaccine** |
| --- | --- | --- | --- | --- | --- | --- | --- |
| **Omicron (B.1.1.529)** | | | | | | | |
| Overall | 31 | 0.397 (0.372, 0.424) | <0.001/99.4 | 1-10 | 60.3 (57.6, 62.8) | Any BNT162b2 or mRNA-1273 | RNA-based vaccine |
|  | 2 | 0.498 (0.407, 0.609) | 0.021/81.3 | 10-15 | 50.2 (39.1, 59.3) |  |  |
|  | 1 | 0.545 (0.528, 0.562) | NA | ≥15 | 45.5 (43.8, 47.2) |  |  |
|  | 4 | 0.611 (0.577, 0.647) | <0.001/97.9 | >8 | 38.9 (35.3, 42.3) |  |  |
|  | 2 | 0.572 (0.516, 0.635) | <0.001/97.7 | ≥10 | 42.8 (36.5, 48.4) |  |  |
| Overall | 4 | 0.431 (0.376, 0.493) | <0.001/98.1 | 1-5 | 56.9 (50.7, 62.4) | BNT162b2/BNT162b2/BNT162b2 |  |
|  | 4 | 0.501 (0.435, 0.576) | <0.001/88.9 | 4-10 | 49.9 (42.4, 56.5) |  |  |
|  | 2 | 0.543 (0.533, 0.553) | 0.687/0.0 | ≥10 | 45.7 (44.7, 46.7) |  |  |
| Overall | 1 | 0.260 (0.251, 0.269) | NA | 1-2 | 74.0 (73.1, 74.9) | BNT162b2/BNT162b2/mRNA-1273 |  |
|  | 1 | 0.261 (0.254, 0.269) | NA | 2-5 | 73.9 (73.1, 74.6) |  |  |
|  | 1 | 0.356 (0.339, 0.374) | NA | 5-10 | 64.4 (62.6, 66.1) |  |  |
| Overall | 5 | 0.446 (0.353, 0.564) | <0.001/93.1 | 1-6 | 55.4 (43.6, 64.7) | mRNA-1273/mRNA-1273/mRNA-1273 |  |
|  | 1 | 0.614 (0.469, 0.806) | NA | 6-8 | 38.6 (19.4, 53.1) |  |  |
| Overall | 1 | 0.357 (0.332, 0.383) | NA | 1-2 | 64.3 (61.7, 66.8) | mRNA-1273/mRNA-1273/BNT162b2 |  |
|  | 1 | 0.351 (0.327, 0.377) | NA | 2-5 | 64.9 (62.3, 67.3) |  |  |
| Overall | 1 | 0.412 (0.403, 0.422) | NA | 1-2 | 58.8 (57.8, 59.7) | ChAdOx1 nCoV-19/ChAdOx1 nCoV-19/BNT162b2 |  |
|  | 1 | 0.376 (0.370, 0.382) | NA | 2-5 | 62.4 (61.8, 63.0) |  |  |
|  | 1 | 0.471 (0.463, 0.479) | NA | 5-10 | 52.9 (52.1, 53.7) |  |  |
|  | 1 | 0.604 (0.589, 0.620) | NA | ≥10 | 39.6 (38.0, 41.1) |  |  |
| Overall | 1 | 0.320 (0.311, 0.330) | NA | 1-2 | 68.0 (67.0, 68.9) | ChAdOx1 nCoV-19/ChAdOx1 nCoV-19/mRNA-1273 |  |
|  | 1 | 0.299 (0.293, 0.305) | NA | 2-5 | 70.1 (69.5, 70.7) |  |  |
|  | 1 | 0.391 (0.379, 0.403) | NA | 5-10 | 60.9 (59.7, 62.1) |  |  |
| Overall | 1 | 0.432 (0.426, 0.437) | NA | 1-8 | 56.8 (56.3, 57.4) | CoronaVac/CoronaVac/BNT162b2 |  |
|  | 1 | 0.651 (0.644, 0.657) | NA | >8 | 34.9 (34.3, 35.6) |  |  |
| Overall | 1 | 0.423 (0.287, 0.624) | NA | 1-2 | 57.7 (37.6, 71.3) | ChAdOx1 nCoV-19/ChAdOx1 nCoV-19/ChAdOx1 nCoV-19 | Viral vector (non-replicating) |
|  | 1 | 0.444 (0.354, 0.556) | NA | 2-5 | 55.6 (44.4, 64.6) |  |  |
|  | 1 | 0.533 (0.433, 0.657) | NA | 5-10 | 46.7 (34.3, 56.7) |  |  |
| Overall | 1 | 0.850 (0.820, 0.880) | NA | 1-8 | 15.0 (12.0, 18.0) | CoronaVac/CoronaVac/CoronaVac | Inactivated virus |
|  | 1 | 0.996 (0.971, 1.022) | NA | >8 | 0.4 (-2.2, 2.9) |  |  |
| **Omicron** **BA.2** | | | | | | | |
| Overall | 3 | 0.324 (0.254, 0.415) | <0.001/98.5 | 2-15 | 67.6 (58.5, 74.6) | Any BNT162b2 or mRNA-1273 | RNA-based vaccine |
|  | 1 | 0.516 (0.486, 0.548) | NA | ≥15 | 48.4 (45.2, 51.4) |  |  |

^#^ Vaccine effectiveness = 100*(1–RR/OR) %; ^&^ NA = not available
