# Supplementary material for: A comprehensive analysis of the efficacy and effectiveness of COVID-19 vaccines
Source: Front Immunol. 2022 Aug 26;13:945930. doi: 10.3389/fimmu.2022.945930 (PMC9459021; doi:10.3389/fimmu.2022.945930)
Supplement: Supplementary file 14 [file Table_13.docx]

**Supplementary Table 13** The duration of effectiveness of COVID-19 vaccine booster immunization against severe COVID-19 caused by Omicron (B.1.1.529) variant

| **Variant** | **No. of studies** | **Adjust RR/OR (95% CI)** | ***P*_h_/*I*^2^ (%)** ^&^ | **Time interval (week)** | **VE (%) (95% CI) ^#^** | **Vaccine name** | **Types of booster vaccine** |
| --- | --- | --- | --- | --- | --- | --- | --- |
| Cohort studies | | | | | | | |
| Overall | 6 | 0.052 (0.034, 0.080) | 0.015/64.6 | 2-8 | 94.8 (92.0, 96.6) | Any BNT162b2 or mRNA-1273 | RNA-based vaccine |
|  | 5 | 0.176 (0.073, 0.426) | 0.002/77.2 | > 8 | 82.4 (57.4, 92.7) |  |  |
|  | 1 | 0.130 (0.120, 0.160) | NA | 2-11 | 87.0 (84.0, 88.0) |  |  |
|  | 1 | 0.210 (0.170, 0.250) | NA | ≥ 11 | 79.0 (75.0, 83.0) |  |  |
|  | 1 | 0.120 (0.100, 0.150) | NA | 8-17 | 88.0 (85.0, 90.0) |  |  |
|  | 1 | 0.220 (0.150, 0.330) | NA | ≥17 | 78.0 (67.0, 85.0) |  |  |
| Case-control studies | | | | | | | |
| Overall | 14 | 0.168 (0.123, 0.227) | <0.001/75.5 | 1-2 | 83.2 (77.3, 87.7) | Any BNT162b2 or mRNA-1273 | RNA-based vaccine |
|  | 16 | 0.113 (0.080, 0.160) | <0.001/94.9 | 2-5 | 88.7 (84.0, 92.0) |  |  |
|  | 16 | 0.148 (0.109, 0.200) | <0.001/96.2 | 5-10 | 85.2 (80.0, 90.1) |  |  |
|  | 16 | 0.184 (0.134, 0.252) | <0.001/96.1 | 10-15 | 81.6 (74.8, 86.6) |  |  |
|  | 17 | 0.171 (0.148, 0.197) | <0.001/93.1 | ≥15 | 82.9 (80.3, 85.2) |  |  |
| Overall | 1 | 0.145 (0.130, 0.162) | NA | 1-8 | 85.5 (83.8, 87.0) | CoronaVac/CoronaVac/BNT162b2 |  |
|  | 1 | 0.139 (0.129, 0.150) | NA | >8 | 86.1 (85.0, 87.1) |  |  |
| Overall | 1 | 0.287 (0.208, 0.397) | NA | 1-8 | 71.3 (60.3, 79.2) | CoronaVac/CoronaVac/CoronaVac | Inactivated virus |
|  | 1 | 0.346 (0.312, 0.385) | NA | >8 | 65.4 (61.5, 68.8) |  |  |

^#^ Vaccine effectiveness = 100*(1–RR/OR) %; ^&^ NA = not available
